# Systemic changes induced by autologous stem cell ovarian transplant in plasma proteome of women with impaired ovarian reserves

**DOI:** 10.18632/aging.205400

**Published:** 2023-12-26

**Authors:** Anna Buigues, Noelia Ramírez-Martin, Jessica Martínez, Nuria Pellicer, Marcos Meseguer, Antonio Pellicer, Sonia Herraiz

**Affiliations:** 1 IVIRMA Global Research Alliance, IVI Foundation - Instituto de Investigación Sanitaria La Fe (IIS La Fe), Valencia 46026, Spain; 2 IVIRMA Global Research Alliance, IVIRMA Valencia, Valencia 46015, Spain; 3 IVIRMA Global Research Alliance, IVIRMA Rome, Rome 00197, Italy

**Keywords:** plasma proteomic profile, autologous stem cell ovarian transplantation, poor ovarian response, premature ovarian insufficiency, aging

## Abstract

Patients with poor ovarian response (POR) and premature ovarian insufficiency (POI) are challenging to treat, with oocyte donation remaining as the only feasible option to achieve pregnancy in some cases. The Autologous stem cell ovarian transplantation (ASCOT) technique allows follicle development, enabling pregnancies and births of healthy babies in these patients. Previous results suggest that growth factors and cytokines secreted by stem cells are partially responsible for their regenerative properties. Indeed, ASCOT beneficial effects associate with the presence of different bone marrow derived stem cell- secreted factors in plasma. Therefore, the aim of this study was to assess whether ASCOT induce any modifications in the plasma proteomic profile of patients with impaired ovarian reserves.

Discriminant analysis highlighted clear distinctions between the plasma proteome before (PRE), during stem cell mobilization and collection (APHERESIS) and three months after ASCOT (POST) in patients with POR and POI. Both the stem cell mobilization and ASCOT technique induced statistically significant modifications in the plasma composition, reversing some age-related protein expression changes. In the POR group, functional analysis revealed an enrichment in processes related to the complement cascade, immune system, and platelet degranulation, while in the POI group, enriched processes were also associated with responses to oxygen-containing compounds and growth hormones, and blood vessel maturation. In conclusion, our findings highlight the potential proteins and biological processes that may promote the follicle activation and growth observed after ASCOT. Identifying plasma proteins that regenerate aged or damaged ovaries could lead to more effective, targeted and/or preventive therapies for patients.

## INTRODUCTION

Patients with poor ovarian response (POR) or premature ovarian insufficiency (POI) are challenging to treat, with oocyte donation remaining as the only feasible option to achieve pregnancy. Previous attempts to overcome the fertility problems of these patients have mainly been based on ovarian stimulation, and were likely unsuccessful [1] due to the lack of stimulable antral follicles remaining in the ovaries [2]. However, emerging strategies based on platelet-rich plasma, ovarian fragmentation, and stem cell injection have reactivated residual dormant follicles to regain ovarian function [3, 4]. One of these strategies is the autologous stem cell ovarian transplantation (ASCOT), consisting of bone marrow derived stem cell (BMDSC) mobilization to peripheral blood by granulocyte-colony stimulating factor (G-CSF) treatment, collection by apheresis and infusion into the ovarian artery [5]. Our group described that the ASCOT procedure improved anti-müllerian hormone (AMH) levels and antral follicular count (AFC), as well as the number of recovered oocytes after ovarian stimulation. Ultimately, this technique enabled pregnancies and births of healthy babies in women with POR and long-lasting infertility, that were previously limited to oocyte donation [5]. Similar evidence of ovarian rescue was obtained in POI patients, in which the reactivation of ovarian function led to more efficient ovarian stimulation, and ultimately, the generation of viable embryos to transfer [6]. Interestingly, follicular growth waves and spontaneous pregnancies were observed up to six months after the ASCOT procedure [5–7], suggesting that this technique might restore folliculogenesis and produce sustained changes in the ovary through the regeneration of the ovarian stroma, as confirmed by our previous studies in mice [8].

Once mobilized, the BMDSCs circulate in peripheral blood, where they secrete paracrine growth factors that are partially responsible for their regenerative properties. Indeed, the levels of growth factors and cytokines in blood change with age. Blood from younger individuals is characterized by a myriad of growth factors and low levels of pro-inflammatory cytokines, while blood from older individuals contains fewer growth factors and an abundance of pro-inflammatory cytokines [9, 10]. Thus, the human plasma proteome may dually reflect aspects of cellular and tissue aging [11] and have an active role in the process. In this study, we aimed to assess if the ASCOT technique modifies the signature of the human plasma proteome, reveal the mechanisms underlying its beneficial effects on the ovary, and identify key regulators of ovarian aging.

## RESULTS

### Proteomic changes induced by ASCOT in patients with POR

Firstly, we assessed the proteomic changes induced by the ASCOT technique in women with POR defined according to the European Society of Human Reproduction and Embryology (ESHRE) criteria [12]. These POR patients (mean age: 36 ± 1 years, 3.0 ± 0.0 years of infertility) showed serum AMH levels of 2.2 ± 0.5 pmol/L, and an AFC of 2.3 ± 0.6 follicles at recruitment. Three months after ASCOT treatment, AMH increased to 2.6 ± 0.9 pmol/L and AFC to 5.0 ± 1.7 follicles. The impact of ASCOT on reproductive outcomes was previously published [5].

**Figure 1:**
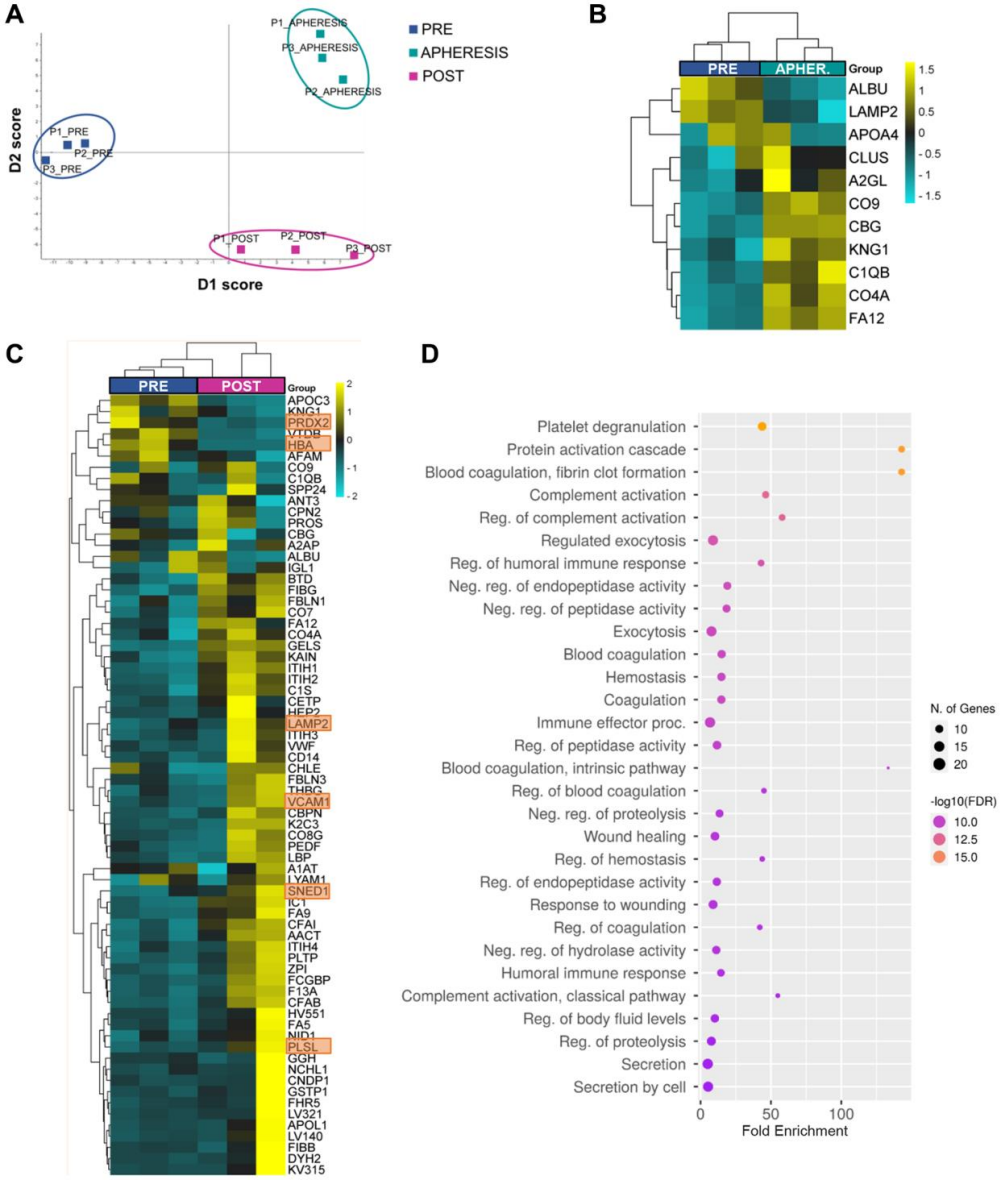
**Plasma proteomic changes following autologous stem cell ovarian transplantation in patients with poor ovarian response.** (**A**) Discriminant analysis plot considering the expression of the 296 quantified proteins, showing a clear separation between the PRE, APHERESIS, and POST samples. (**B**) Heatmaps depicting the hierarchical clustering of the 11 differentially expressed proteins (DEPs) between PRE and APHERESIS samples and (**C**) 70 DEPs between PRE and POST samples. The most prominent changes after ASCOT, considering the estimated fold change, highlighted in orange. Dot plot showing the top 30 significantly enriched (FDR < 0.05) GO biological processes three months after autologous stem cell ovarian transplantation (**D**). The heatmaps are color coded according to protein expression determined by SWATH^™^ analysis, where yellow and turquoise indicate an increase or decrease in expression, respectively.

To elucidate whether ASCOT technique changes the plasma composition of patients with POR undergoing this reactivation technique, the proteomic profile of peripheral blood plasma was assessed before (PRE), during stem cell mobilization and collection by apheresis (APHERESIS), and three months after stem cell infusion into the ovary (POST; Supplementary Table 1). Considering the expression of the total 296 proteins quantified, the analyses of dimensionality reduction (discriminant analysis and principal component analysis) showed a clear distinction between PRE, APHERESIS, and POST samples (Figure 1A and Supplementary Figure 1A; D1: 50%, D2: 50%, PC1: 38.9%, PC2: 16.8%), confirming changes in plasma composition associated to the stem cell mobilization procedure and the ASCOT treatment.

Among the 296 proteins, 11 (3.7%) were found differentially expressed between PRE and APHERESIS conditions (Figure 1B and Supplementary Table 2), while 70 (23.6%) were differentially expressed between PRE and POST samples (Supplementary Table 3). Interestingly, eight differentially expressed proteins (DEPs) were shared between both comparisons (Supplementary Figure 1B), suggesting that the BMDSCs induced changes in plasma composition that remained for several months. Of these, the corticosteroid-binding globulin (CBG), coagulation factor XII (FA12), kininogen-1 (KNG1), along with the complement C1q B chain (C1QB), C4-A (CO4A), and component C9 (CO9) were upregulated in both the APHERESIS and POST samples, while serum albumin (ALBU) and lysosome-associated membrane glycoprotein 2 (LAMP2) were repressed.

Focusing on those effects observed three months after ASCOT, in general the heatmap analysis showed a clear separation between PRE and POST samples, with the exception of one patient in which the ASCOT technique seemed to have a lesser effect (Figure 1C). The most prominent changes after ASCOT, considering the estimated fold change, included the sushi, nidogen, and EGF like domains 1 (SNED1), plastin-2 (PLSL), vascular cell adhesion molecule 1 (VCAM1), hemoglobin subunit alpha (HBA) and peroxiredoxin-2 (PRDX2) (Supplementary Table 2).

To establish the biological relevance of the plasma changes induced by ASCOT, we queried the GO Biological Process database and measured protein enrichment. This analysis showed none significantly associated GO biological process in APHERESIS but more than 500 pathways in POST samples (False Discovery Rate, FDR < 0.05), being the top 30 related to protein activation, fibrin clot cascades, complement cascades, immune response, hemostasis, wound healing and exocytosis (Figure 1D).

Moreover, to understand if stem cell mobilization and infusion has the ability to restore the plasma composition, we sought to determine whether the 70 proteins whose expression significantly changed after ASCOT were linked to aging. Thus, we compared these DEPs between POST and PRE samples with proteins that change in blood with age [10, 11], and 9 were associated with aging. Among them, the expression of seven was decreased with age, but rescued with ASCOT (i.e., VCAM1, nidogen-1 (NID1), inter-alpha-trypsin inhibitor heavy chain H1 (ITIH1), thyroxine-binding globulin (THBG), pigment epithelium-derived factor (PEDF), apolipoprotein L1 (APOL1), and alpha-2-antiplasmin (A2AP)), while the remaining two were overexpressed with age and repressed following treatment (i.e., Apolipoprotein C-III (APOC3) and Vitamin D-binding protein (VTDB)).

### Proteomic changes induced by ASCOT in patients with POI

The systemic effects of stem cell mobilization and injection in patients with POI were also assessed. This second sub-study included 6 women (mean age 34 ± 2 years, 4.0 ± 5.4 years of infertility) with POI according to the ESHRE criteria [13]. AMH and follicle-stimulating hormone (FSH) basal levels in these patients were 0.09 ± 0.08 pmol/L and 99.9 ± 23.9 IU/ml, respectively, while AFC was 1.5 ± 1.2 follicles. Prior to the reactivation technique and due to the POI diagnose, these women underwent only a total of 2 IVF attempts consisting in 1 natural cycle without mature oocytes recovered and 1 cancellation due to the absence of response. Once recruited, these patients were randomized into two trial arms: 1) ASCOT group, in which the stem cells were mobilized and infused into the ovary, as described for POR women; 2) Mobilization (MOB) group, where the stem cells were mobilized but remained circulating in peripheral blood (without collection and direct ovarian infusion). The proteomic profile was assessed at the three time points previously described: at recruitment (PRE), during stem cell mobilization and collection (APHERESIS) and three months after stem cell mobilization or injection (POST). Three months after the ASCOT technique, AMH and AFC increased, and FSH decreased (Supplementary Table 4). Moreover, a total of 11 cycles of ovarian hyperstimulation were performed in these 6 patients after ASCOT (Supplementary Table 5), with a total of 9 punctured follicles, 3 collected MII oocytes and 2 embryos.

#### Systemic effects of stem cell mobilization

**Figure 2:**
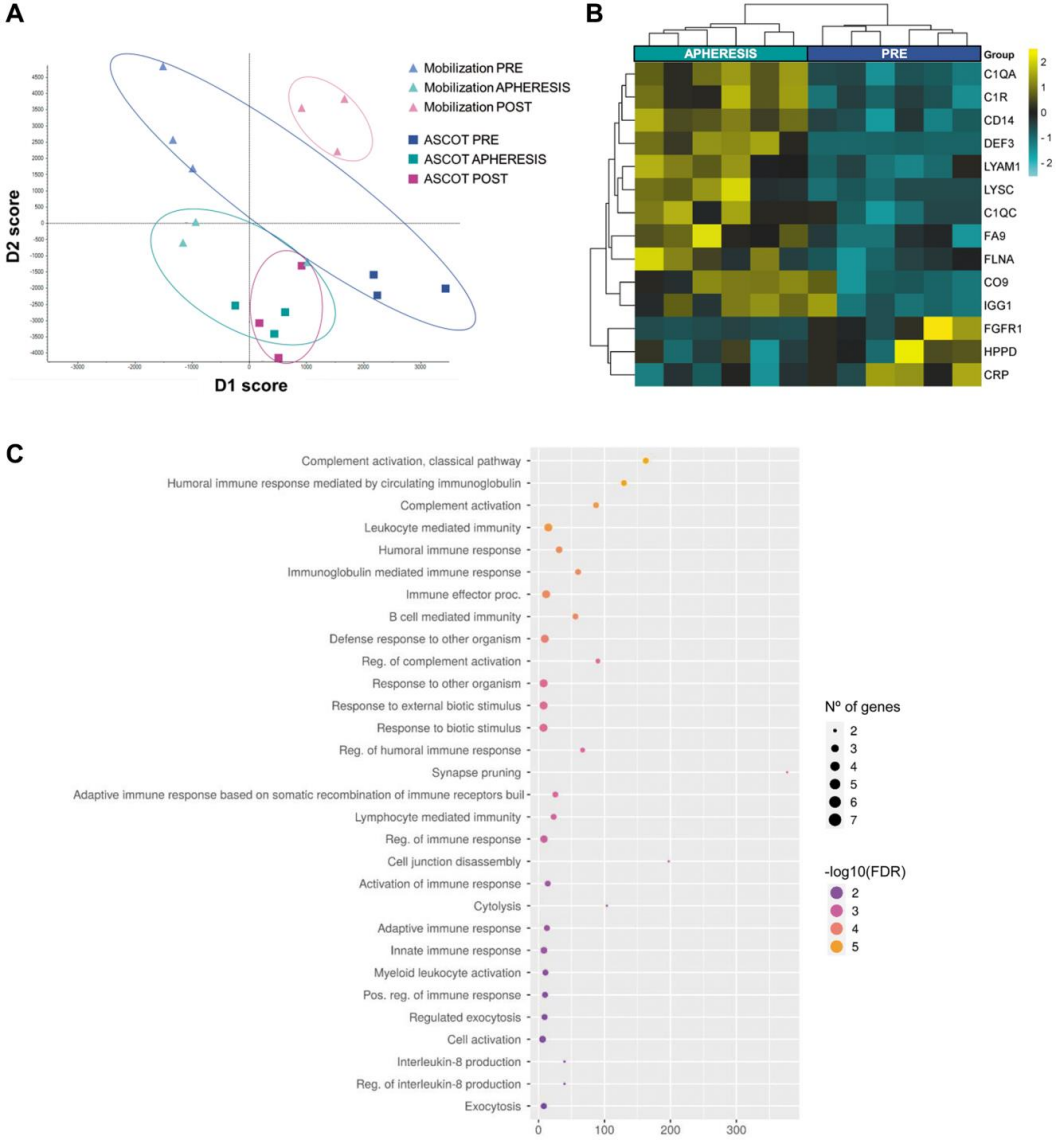
**Plasma proteomic changes following stem cell mobilization in patients with premature ovarian insufficiency.** (**A**) Discriminant analysis plot considering all PRE and all APHERESIS samples together. ASCOT, autologous stem cell ovarian transplantation. Heatmap depicting the hierarchical clustering of the 14 differentially expressed proteins between the PRE and APHERESIS samples (**B**), and dot plot showing the corresponding top 30 significantly enriched (FDR < 0.05) GO biological processes (**C**). The heatmap is color coded according to protein expression determined by SWATH^™^ analysis, where yellow and turquoise indicate an increase or decrease in expression, respectively.

A total of 431 proteins were quantified among all samples from the patients with POI (Supplementary Table 6). The analysis of dimensionality reduction showed a clear distinction between the PRE and APHERESIS samples (Figure 2A; D1: 50%, D2: 50%), characterized by the presence of 14 DEPs (3.2% of the total; Figure 2B). Specifically, both the complement C1q subcomponent subunit A (C1QA) and complement C1r subcomponent (C1R) exhibited the highest change for the estimated fold change (Supplementary Table 7). Accordingly, functional analysis highlighted significant enrichment of pathways related to complement cascades and immune response (Figure 2C). Moreover, when the link of these proteomic changes with aging was evaluated, we found three proteins that were upregulated in APHERESIS whose plasma levels decrease with aging (i.e., complement C1q subcomponent subunit C (C1QC), lysozyme C (LYSC), and L-selectin (LYAM1)), and one downregulated protein (i.e., C-reactive protein (CRP)) whose expression rises with age [11].

Interestingly, the POST samples showed a clearly different proteomic pattern in each study arm (Figure 2A), highlighting an effect of stem cell infusion. Therefore, each arm was considered independently to determine the systemic effects of stem cell mobilization and injection (with respect to their corresponding PRE groups).

#### Specific effects in the mobilization arm

**Figure 3:**
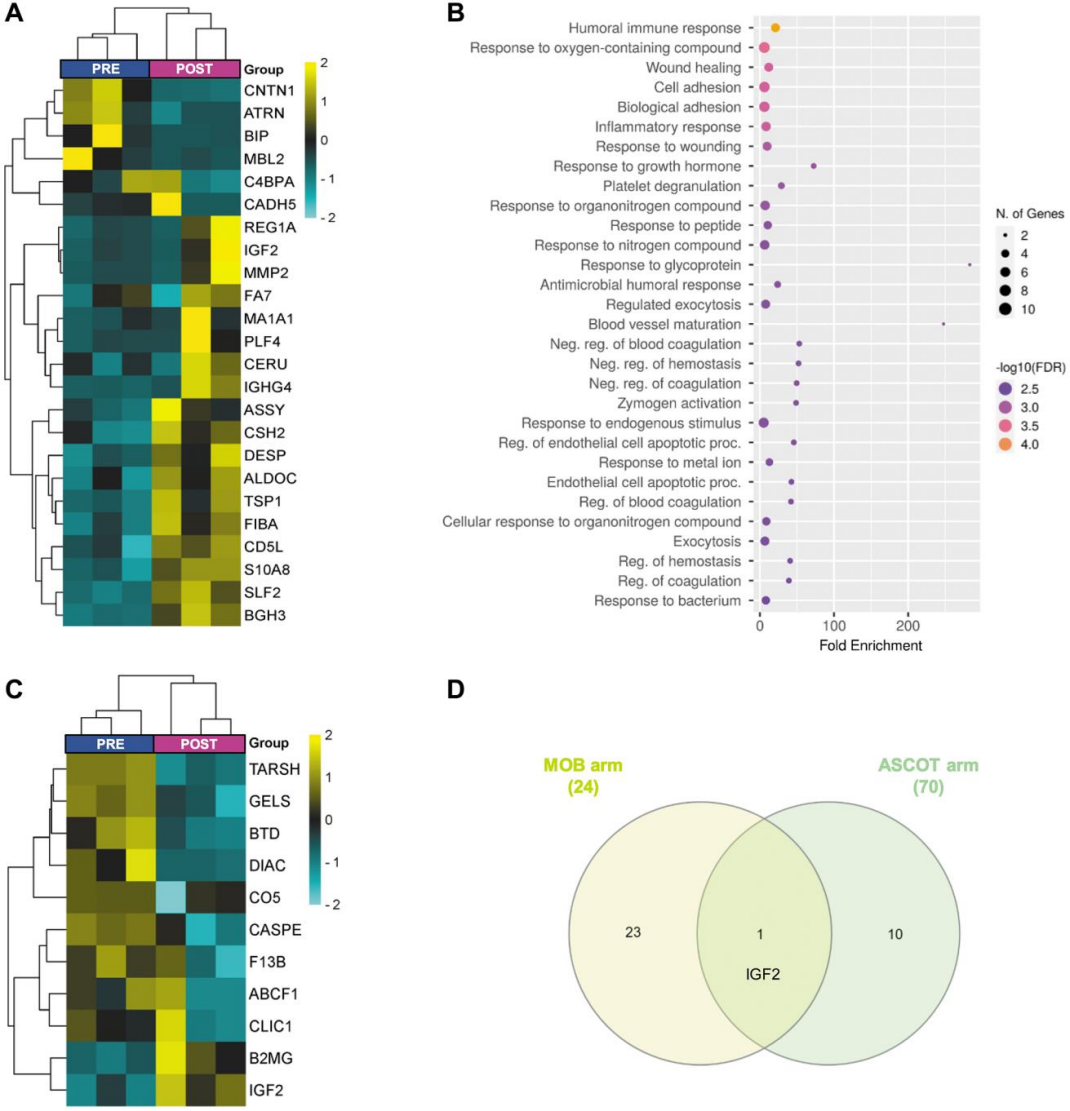
**Plasma proteomic changes three months after stem cell mobilization/injection in patients with premature ovarian insufficiency.** Heatmap depicting the hierarchical clustering of the 24 differentially expressed proteins (DEPs)between PRE and POST samples in the mobilization arm (**A**), and dot plot showing the corresponding top 30 significantly enriched (FDR < 0.05) GO biological processes (**B**). (**C**) Heatmap depicting the hierarchical clustering of the 11 DEPs between PRE and POST samples in the ASCOT arm. (**D**) Venn diagram highlighting the shared DEPs between POST samples of both study arms. The heatmaps are color coded according to protein expression determined by SWATH^™^ analysis, where yellow and turquoise indicate an increase or decrease in expression, respectively.

Within the mobilization arm, we identified 24 DEPs between PRE and POST groups (5.6% of the total; Figure 3A; Supplementary Table 8), suggesting that stem cell mobilization, alone, can elicit systemic changes that persist over time. Next, functional analysis revealed that these changes were associated with responses related to the immune system, oxygen-containing compounds, wounding, and growth hormones, in addition to cell adhesion, platelet degranulation, and blood vessel maturation (Figure 3B). Notably, increased plasma levels of two DEPs (i.e., 72 kDa type IV collagenase (MMP2) and fructose-bisphosphate aldolase C (ALDOC)) which were described to decrease with age [11].

#### *Specific effects of stem cell injection* (*ASCOT arm*)

To determine how stem cell injection affected plasma proteome, we compared POST samples of patients included in the ASCOT arm with their corresponding PRE samples.

These samples were also clearly differentiated (Figure 2A), although only 11 DEPs were identified (Figure 3C, Supplementary Table 9), and no significantly associated GO Biological Processes were found. Nevertheless, amidst these DEPs, we found the di-N-acetylchitobiase (DIAC) whose plasma levels increase with aging [11] and was downregulated after ASCOT.

Additionally, the comparison between POST samples from both study arms identified only one shared DEP (Figure 3D), suggesting that those proteomic changes in peripheral plasma after the technique depend not only on stem cell mobilization, but also on ovarian injection. Indeed, 16 DEPs (Supplementary Figure 2A; Supplementary Table 10) related to platelet degranulation, catabolic processes, immune response, blood coagulation, proteolysis, complement cascades, and plasma lipoprotein oxidation (Supplementary Figure 2B), were found between POST samples of both study arms.

### Comparative assessment of plasma proteomic changes associated to the ASCOT technique in patients with POR and POI

Finally, we compared the DEPs between patients with POR and POI to assess if modifications of proteomic profiles after stem cell mobilization and infusion change according to diagnosis.

**Figure 4:**
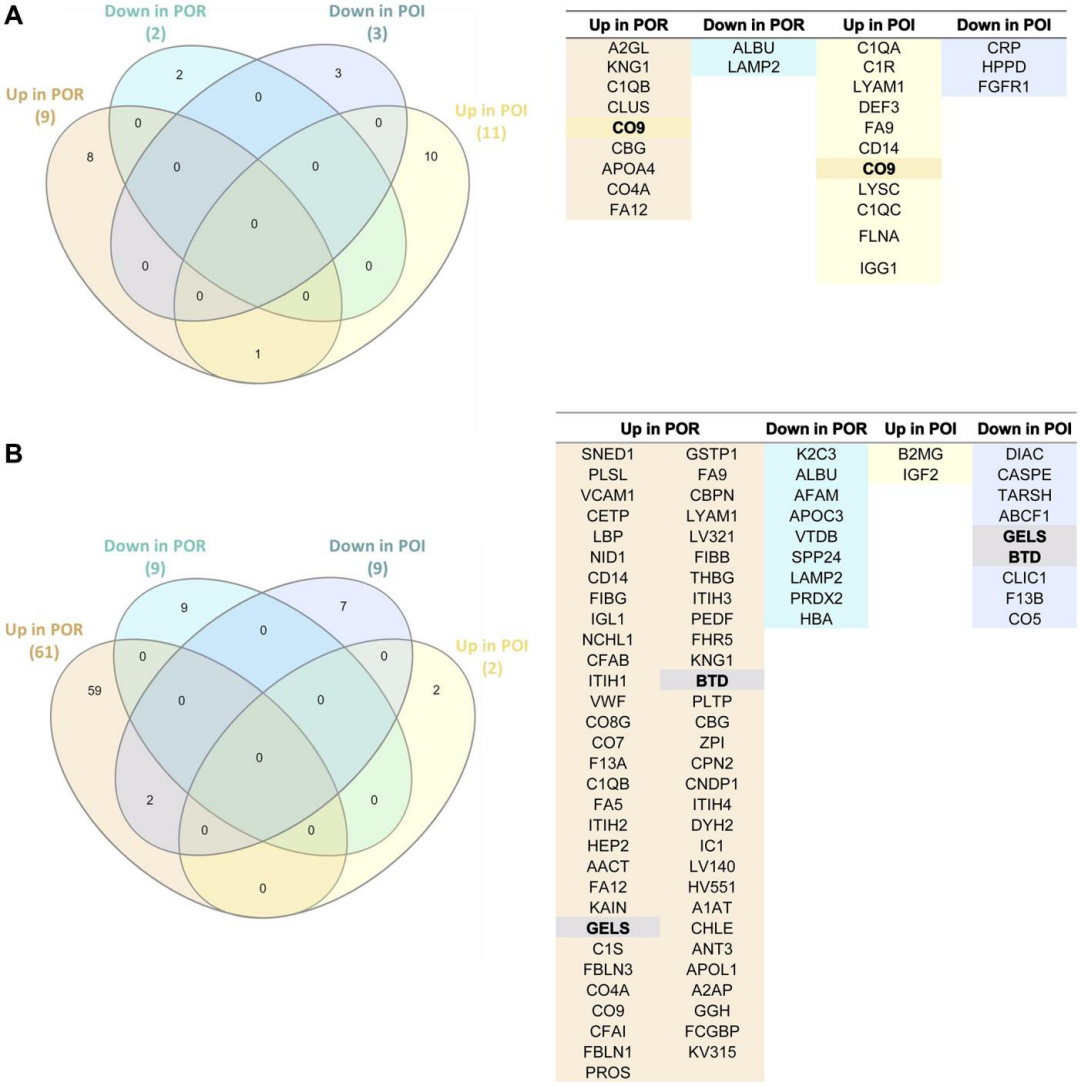
**Comparative assessment of the proteomic effects of stem cell mobilization and injection in patients with poor ovarian response (POR) and premature ovarian insufficiency (POI).** (**A**) Venn diagram (left) and protein name (right) of the differentially expressed proteins in APHERESIS samples of patients with POR and POI (both study arms), showing the relationships between the type of protein regulation (up/down). (**B**) Venn diagram (left) and protein name (right) of the differentially expressed proteins three months after autologous stem cell ovarian transplantation (ASCOT) in patients with POR and patients with POI from the ASCOT arm, showing the relationships between the type of protein regulation (up/down). The total number of significant differentially expressed proteins in each category is represented in brackets.

We found that only one protein (CO9) was commonly upregulated between the APHERESIS samples of patients with POR and POI after stem cell mobilization (Figure 4A).

The stem cell infusion procedure also induced different systemic changes between patients with POR and POI patients from the ASCOT arm (with cell infusion), as shown in Figure 4B. In fact, the only two shared DEPs following stem cell injection were biotinidase (BTD) and gelsolin (GELS), which were upregulated in POR and downregulated in POI patients.

## DISCUSSION

Blood is considered a sensitive marker for functional aging, with an active role in this process. In fact, several studies have shown that the soluble factors present in blood from younger individuals can reverse some aspects and clinical signs of aging [9, 14–16]. Based on these findings, along with the long-term fertility restoration induced by ASCOT in women with POR/POI and our murine models of ovarian damage, we aimed to evaluate the plasma proteome modifications induced by ASCOT, with special interest in those proteins with potential ovarian regenerative properties.

Our functional analyses suggested that, overall, the ASCOT technique affects biological processes related to platelet activation and degranulation, the immune system and the complement cascade. Platelet release of growth factors becomes less efficient with age [10], and alterations in immune responses have been proposed as major causes of ovarian aging [17]. Likewise, complement cascades are related to folliculogenesis, oocyte maturation, and ovarian aging [18, 19]. In this context, our results suggest that the promotion of follicle growth and ovulation observed after ASCOT [5] may be elicited by the proteomic changes of the complement system.

In the POR cohort, almost all the DEPs in the APHERESIS samples were also detected in the POST samples, suggesting that BMDSCs induced proteomic changes in plasma composition that remain over time and that could underlie the second wave of follicular growth previously observed 3 months after ASCOT, and numerous spontaneous pregnancies reported more than 6 months after the procedure [5]. Of the DEPs from the POST samples, the most prominent change was observed for SNED1, an extracellular matrix (ECM) protein. Notably, the ECM is altered with ovarian aging [20, 21], and affects follicle development, oocyte quality, fibrosis, and vascularization [22–26]. In fact, a restoration of ovulation and an extension of the reproductive lifespan of female mice was achieved by reverting the ovarian fibrosis occurring with aging [27]. Therefore, the promotion of follicle growth observed after ASCOT [5] could be related to changes in the ECM, as our previous studies using murine models suggested [8], and SNED1 might play a key role in these effects within the ovarian niche. Moreover, the ASCOT procedure increased proteins whose levels are reduced with aging (such as VCAM1, NID1, ITIH1, THBG, PEDF, APOL1, and A2AP), and repressed others that increase with age (i.e., APOC3 and VTDB) [10, 11]. These findings supported that ASCOT may partially reverse some age-related proteomic changes in plasma, providing factors with potential regenerative properties in the ovaries. It is important to note that the levels of VCAM1, ITIH1, and APOL1 are reduced in the wave of plasma protein changes that occurs at the age of 34 [11], when ovarian aging begins [28]. VCAM1 plays a crucial role in age-related ovarian vascular alterations, such as increased oxidative stress, vascular fibrosis, and endothelial barrier impairment [17, 29, 30]. Thus, the ASCOT technique might optimize ovarian function by reverting the decline of the aforementioned proteins, which are likely involved in ovarian aging. However, further investigation of the proteins we identified is warranted to determine whether they truly play a role in the ovarian regenerative effects observed after ASCOT in women with POR.

In patients with POI, stem cell mobilization also modified the plasma proteomic profile, however the DEPs in APHERESIS samples differed from those observed in patients with POR, with only one common upregulated protein. This finding suggests that although mobilization may affect similar biological processes, the downstream effects depend on the diagnosis of the patient. This finding is consistent with previous data showing differences in apheresis composition with menstrual cycle phase and hormone levels [31]. In fact, although POI and POR patients can be mixed up, they represent different clinical entities, showing differences in menses cyclicity and sexual hormone profile.

Our study revealed that the G-CSF mobilization treatment, *per se*, induced changes in the plasma proteomic composition still detectable 3 months after treatment, that might be linked to follicular growth observed in patients of the mobilization arm [6, 7]. This is consistent with tissue regeneration induced by G-CSF treatment, due to the ability of BMDSCs to migrate and engraft in damaged tissues and organs [32–35]. These effects of mobilization in women with POI would be related to the immune system and platelet degranulation, responses to oxygen-containing compounds and growth hormones, and blood vessel maturation, which are all processes associated with the oocyte’s bioenergetics and quality [36], folliculogenesis and ovulation [37, 38]. Among those systemic changes induced by G-CSF mobilization that remain detectable for a few months, we observed an up-regulation of MMP2 and ALDOC, whose levels are described to decrease in plasma with aging [11]. MMP2 degrades ECM proteins, and as such, is involved in diverse functions (e.g., vascular remodeling, angiogenesis, tissue repair, and inflammation). Further, the ovarian expression of MMP2 decreased with age [21]. Based on the ovarian niche regeneration and microvessel formation induced by the intravenous administration of BMDSCs in murine models of POI [8], MMP2 appears to be a key player in the beneficial effects of these stem cells. Likewise, ALDOC stimulates follicle activation [39] and, therefore, might be involved in the late wave of follicle growth observed in patients with POI six months after stem cell mobilization [6, 7] – which aligns with the 5–6 month period required for a human primordial follicle to reach the preovulatory stage [40].

On the other hand, direct BMDSCs infusion into the ovary also produced non-transient changes in the plasma proteomic profile of women with POI, however, there were fewer and diverse DEPs from those caused by G-CSF mobilization alone, highlighting an effect of stem cell injection. Interestingly, only insulin-like growth factor II (IGF2) was differentially expressed in POST samples of both study arms. The levels of IGF2 have been reported to decrease in serum and oocytes with aging, and thus, its supplementation could potentially enhance oocyte competence [41, 42]. Besides IGF2, another ten DEPs were found in POST ASCOT samples, including F13B and TARSH, which are dysregulated in women with PCOS [43, 44], and ABCF1, which regulates innate immune responses and DNA repair [45].

In conclusion, our results showed that G-CSF mobilization and our ASCOT technique elicits proteomic changes in peripheral blood plasma composition in both POR and POI women that remain for few months. Specifically, stem cell mobilization and infusion reversed age-related proteomic changes, particularly those occurring in the wave of changes that occurs at the age of 34, when ovarian aging begins [10, 11]. These proteins would be involved in the activation and promotion of follicle growth observed following the ASCOT technique, and could be key regulators of ovarian aging. We aimed to investigate those blood-borne changes induced by the ASCOT technique in these patients, who may already present an altered plasmatic profile, to explain the ovarian effects detected months after ASCOT [5]. However, further investigation including a reference group of age-matched fertile women will be required to assess how the ovarian diagnose affects plasma composition and if the technique restores proteomic condition. Moreover, considering the small sample size used in this study, further experimental studies will also be needed to determine the extent of their regenerative effects within the ovaries, and their direct implications in ovarian aging. Identifying plasma proteins that regenerate aged or damaged ovaries could lead to more effective, targeted and/or preventive therapies for affected patients.

## METHODS

### Ethical approval

All study procedures were approved by the Institutional Review Board of the Hospital Universitario y Politécnico La Fe, in Valencia, Spain (2014/0147 and 2017/0251) and in accordance with the principles expressed in the Declaration of Helsinki.

### Study design

Blood samples from women with POR and POI, included in our previous ASCOT pilot studies (NTC02240342 and NCT03535480), were employed in this study.

Patients with POR (*N* = 3), defined according to the European Society of Human Reproduction and Embryology (ESHRE) criteria [12], were treated with G-CSF (10 mg/kg/day, subcutaneously) during 5 days to mobilize the stem cells from the bone marrow to peripheral blood. On day fifth, the BMDSCs were collected by apheresis, and infused into the ovarian artery by intra-arterial catheterization, as previously described [5]. Meanwhile, as described before, six POI patients according to the ESHRE criteria [13] were divided into two trial arms: (1) ASCOT, in which the stem cells were mobilized and infused into the ovary, as described for POR women; (2) Mobilization (MOB), in which the mobilized stem cells remained circulating in peripheral blood without local transplant in the ovary. Plasma samples were collected before (PRE), during (APHERESIS) and three months after stem cell mobilization and injection (POST), assessing the proteomic profile of each time point by the high throughput quantitative SWATH^™^ technique.

### Sample collection and plasma isolation

The peripheral blood was collected in BD Vacutainer^®^ EDTAK2 tubes (BD Diagnostics, NJ, USA) prior to, and three months after, stem cell mobilization (PRE and POST samples, respectively). An aliquot of the apheresis of each patient was also collected in EDTA tubes. Plasma samples were isolated through centrifugation (for 10 min at 4°C), and stored at −80°C until further use. Plasma samples from three patients with POR and six patients with POI (three in each study arm) were then analyzed by proteomic techniques.

### Proteomic assessment of plasma samples by LC-MS/ MS and SWATH^™^ analysis

A quantitative proteomic approach using SWATH™ was applied to analyze the proteomic profiles of plasma from PRE, APHERESIS, and POST samples.

To prepare the samples for proteomic analysis, 150 μL of each plasma sample was centrifuged at 15,000 × g for 15 min at 5°C, to separate the lipoproteins present in the circulating blood. Samples were then pooled to build a library, as described in the Supplementary Materials.

Once the library was generated, the PRE, APHERESIS, and POST samples from the POR and POI patients were analyzed individually. The proteins were extracted from each sample, quantified, and digested as described in the Supplementary Materials. Digested peptide mixtures (5 μL) were loaded onto NanoLC Columns (3 μ C18-CL, 75 μm × 15 cm; Eksigent Technologies, CA, USA) and desalted with 0.1% trifluoroacetic acid at 2 μL/min during 10 min. Analytical columns (LC Column, 3 μ C18-CL, 75 μm × 12 cm; Nikkyo Japan) were equilibrated in 5% acetonitrile with 0.1% formic acid, before eluting peptides with a linear gradient of 5–35% acetonitrile solution in 0.1% formic acid for 120 min (flow rate: 300 nL/min). Peptides were analyzed in a mass spectrometer nanoESI qQTOF (5600 TripleTOF; AB Sciex, MA, USA) operating in SWATH mode, in which a 0.050-s TOF MS scan from 350–1250 m/z was performed, followed by 0.080-s product ion scans from 350–1250 m/z on the 32 defined windows (3.05 sec/cycle). Thirty-seven SWATH windows were used, with 15 Da window widths, from 450 to 1000 Da. The individual samples were randomized in blocks and the total ions were counted. The resulting wiff files were analyzed using Peak View 2.1 with the previously generated “Pan Serum Library”. The processing settings used for the peptide selection are indicated in the Supplementary Materials. After peptide detection, the retention times were realigned using the high-confidence peptides from the library, and peptides were re-analyzed with a 10 min XIC extraction window. Proteins were quantified employing Marker View (Sciex, MA, USA), and the computed protein areas were normalized by the total sum of the areas of all the quantified proteins.

### Statistical analysis

Exploratory multidimensional analysis of proteomic data was performed using principal component analysis and discriminant analysis to reduce dimensionality and explore the results of SWATH^™^ assessment. To this end, R software [46] was employed. Then, a penalized linear regression ElasticNet model was applied using R, and the glmnet library was employed to DEPs among PRE and APHERESIS samples, and PRE and POST samples, in patients with POR and POI. Specifically, after a logarithmic transformation of the SWATH quantified protein areas, the train function of the Caret package was employed to obtain the parameters needed for ElasticNet penalized linear regression.

Finally, to determine the biological processes affected by BMDSCs mobilization and infusion into the ovary, a Gene Ontology Enrichment Analysis for the differentially expressed proteins was performed using the ShinyGO web-tool (version 0.741). GO Biological Processes with a FDR < 0.05 were considered significantly enriched, and the top thirty were selected and represented in dotplot charts.

## Supplementary Materials

Supplementary Methods

Supplementary Figures

Supplementary Tables 1 and 6

Supplementary Tables 2-5 and 7-10
