# Supplementary material for: Systemic changes induced by autologous stem cell ovarian transplant in plasma proteome of women with impaired ovarian reserves
Source: Aging (Albany NY). 2023 Dec 26;15(24):14553–73. doi: 10.18632/aging.205400 (PMC10781467; doi:10.18632/aging.205400)
Supplement: Supplementary Methods [file aging-15-205400-s001.pdf]

SUPPLEMENTARY METHODS

Library construction: sample pool protein extraction, digestion and LC-MS/MS SWATH™

A sample pool was generated by combining 15 µL aliquots from each patient samples. Of this sample pool, 50 µL was precipitated with ice-cold ethanol at a final concentration of 40% during 2 h at 5°C. The sample was then centrifuged for 1 h at 15,000 g and the supernatant (containing human serum albumin (HSA)) was removed. The pellet was left to air dry and the precipitated proteins were validated using 1:1 dilution in 150 nM NaCl loaded onto a 12% SDS-PAGE gel stained with Coomassie Colloidal. To reduce the protein complexity for the LC-MS/MS analysis, the gel was fractioned into slices and incubated overnight at 37°C with 500 ng sequencing grade trypsin (Promega, WI, USA) for enzymatic digestion, as previously described (12). The digestion was stopped with 20% trifluoroacetic acid and the supernatant was removed before dehydrating the library gel slides with pure acetonitrite. The new peptide solutions were combined with the corresponding supernatant, dried in a speed vacuum and resuspended in 2% acetonitrite, 0.1% trifluoroacetic acid. The final volume was adjusted (from 6–10 µL) according to the intensity of the staining.

LC-MS/MS was adapted from a previously described protocol (13) set up in similar samples. Digested peptide mixtures (5 µL) were loaded onto NanoLC Columns (3 µ C18-CL, 75 um × 15 cm; Eksigent Technologies, CA, USA) and desalted with 0.1% trifluoroacetic acid at 3 µl/min during 5 mins. Analytical columns (LC Column, 3 µ C18-CL, 75 um × 12 cm; Nikkyo, Japan) were equilibrated in acetonitrile solution (5% acetonitrile with 0.1% formic acid) before eluting peptides with a linear gradient of 5–35% acetonitrile solution in 0.1% formic acid for 30 min at a flow rate of 300 nL/min. Peptides were analyzed in a mass spectrometer nanoESI qQTOF (5600 TripleTOF; ABSCIEX, MA, USA) and a library was created by combining all the data. ProteinPilot (v.5.0) default parameters were used to generate a peak list directly from 5600 TripleTof wiff files. The Paragon algorithm (14) was used to search the Swissprot database (03.2018) with the following parameters: trypsin specificity, cys-alkylation, without taxonomy restriction, and the search effort set to through and false discovery rate (FDR) correction for proteins. To avoid using the same spectral evidence in more than one protein, the identified proteins were grouped based on MS/MS spectra by the ProteinPilot Progroup algorithm. Furthermore, data obtained in this experiment was combined with all the data of human plasma generated in the proteomic laboratory of the Central Service for Experimental Research (SCSIE; University of Valencia), to create a Pan Serum Spectrum Library, and amplify the number of proteins represented in the library from 337 to 507.

Individual sample protein extraction, quantification and digestion

One hundred µL of each individual plasma sample was dilipidated by centrifugation and 85 µL of delipidated plasma was precipitated with ethanol 40% to remove HSA as explained above. The pellets were dissolved with 100 µL of 0.5 M Tetraethylammonium bromide with 4 M Urea and 2% SDS and the final solutions were quantified with a protein quantification assay (Machery-Nagel, France) according the manufacturer protocol. Samples (25 µg) were then loaded without resolving in 1D PAGE gels and incubated overnight at 37°C with 500 ng sequencing grade trypsin (Promega, WI, USA) for enzymatic digestion, as previously described (12). The digestion was stopped with 20% trifluoroacetic acid and the supernatant was removed before dehydrating the gels with pure acetonitrite. The peptide solutions were dried in a vacuum centrifuge and resuspended in 50 µL 2% acetonitrite, 0.1% trifluoroacetic acid.

Processing settings used for peptide selection in individual samples

The following processing settings were applied to select peptides in the nine individual plasma samples: 20 peptides/protein, 6 transitions/peptide, 95% peptide confidence threshold, 1% FDR, exclude modified peptides. XIC options were set to 20 min XIC extraction windows and 50 ppm for XIC width.
