# Supplementary material for: Systemic changes induced by autologous stem cell ovarian transplant in plasma proteome of women with impaired ovarian reserves
Source: Aging (Albany NY). 2023 Dec 26;15(24):14553–73. doi: 10.18632/aging.205400 (PMC10781467; doi:10.18632/aging.205400)
Supplement: Supplementary Figures [file aging-15-205400-s002.pdf]

SUPPLEMENTARY FIGURES

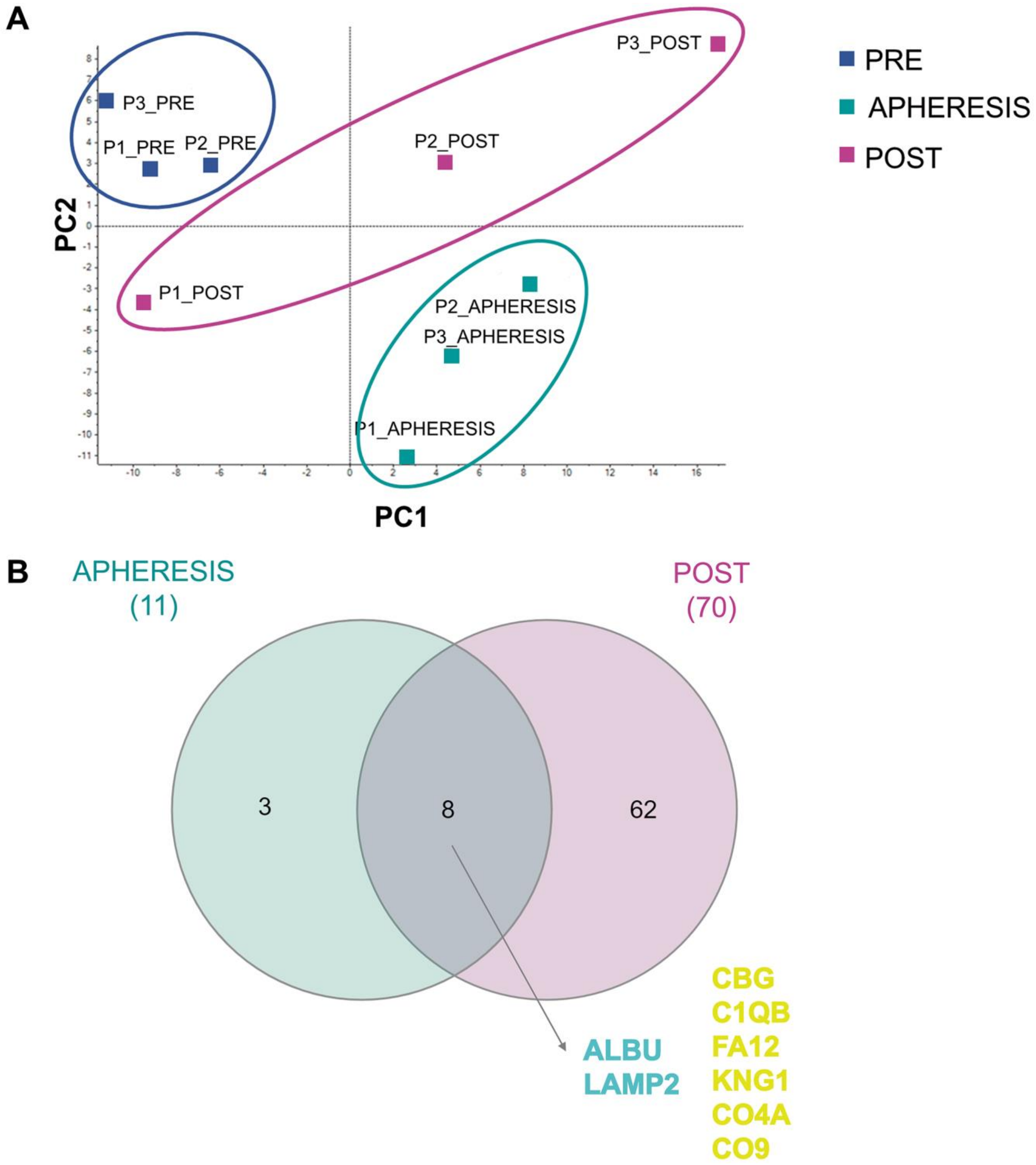

Supplementary Figure 1. Differences and similarities between immediate and lasting proteomic changes induced by stem cell mobilization and injection in women with poor ovarian response (POR). (**A**) Principal component analysis plot considering the expression of the 296 quantified proteins, showing a clear separation between PRE, APHERESIS and POST samples. (**B**) Venn diagram highlighting the share differentially expressed proteins between APHERESIS vs. PRE and POST vs. PRE comparisons.

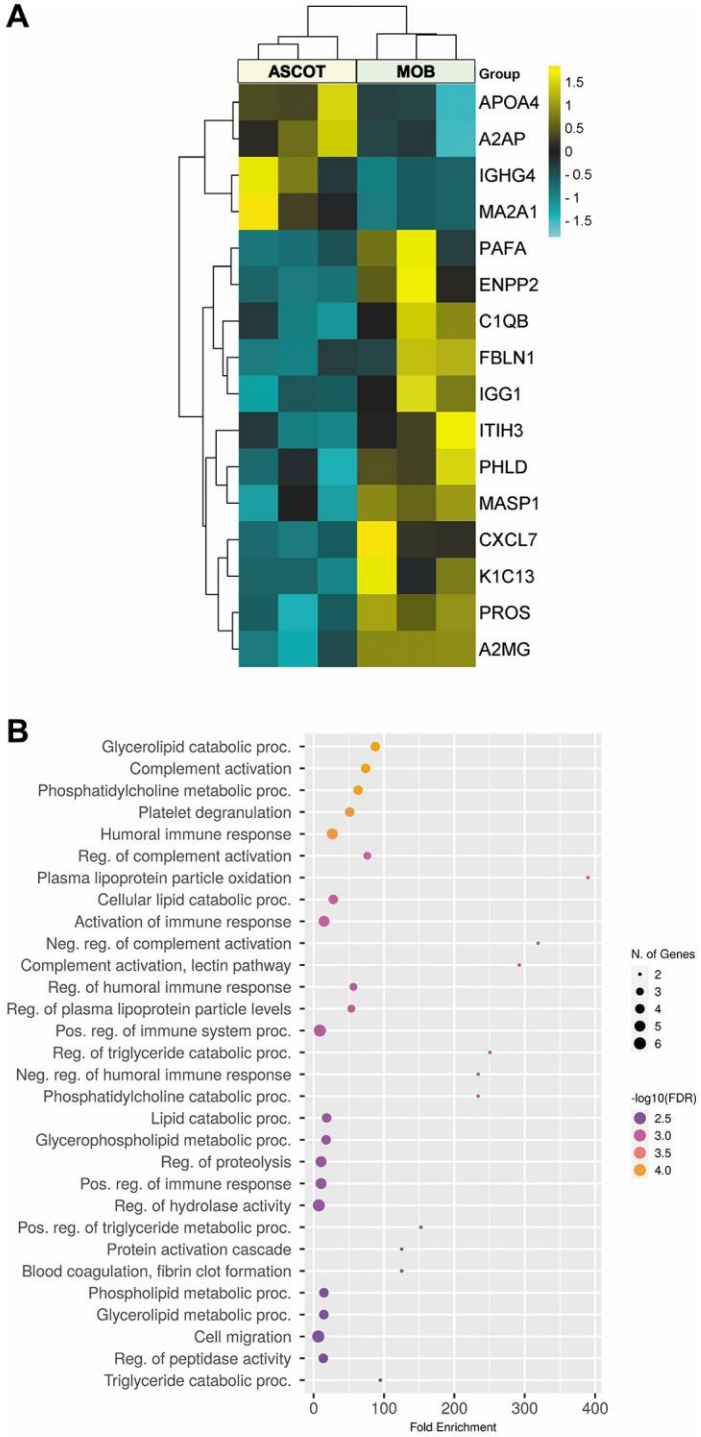

Supplementary Figure 2. Comparison of the proteomic effects induced by stem cell mobilization, *per se,* and by stem cell injection three months after the procedures in women with premature ovarian insufficiency (POI). (**A**) Heatmap depicting the hierarchical clustering of the 16 differentially expressed proteins between POST samples of both study arms: mobilization (MOB) and autologous stem cell ovarian transplantation (ASCOT). (**B**) Dot plot showing the corresponding top 30 significantly enriched (FDR < 0.05) GO biological processes.
