# Supplementary material for: Systemic changes induced by autologous stem cell ovarian transplant in plasma proteome of women with impaired ovarian reserves
Source: Aging (Albany NY). 2023 Dec 26;15(24):14553–73. doi: 10.18632/aging.205400 (PMC10781467; doi:10.18632/aging.205400)
Supplement: Supplementary Tables 1 and 6 [file aging-15-205400-s003.docx]

**Supplementary Table 1. Proteomic profile of plasma before (PRE), during (APHERESIS) and after (POST) stem cell mobilization and infusion in women with poor ovarian response (POR).**

| Protein ID | Protein name | Protein name abbreviation | Gene name | P1_PRE | P2_PRE | P3_PRE | P1_APHERESIS | P2_APHERESIS | P3_APHERESIS | P1_POST | P2_POST | P3_POST |
| --- | --- | --- | --- | --- | --- | --- | --- | --- | --- | --- | --- | --- |
| P04114 | Apolipoprotein B-100 | APOB | APOB | 933481,3 | 688373,3 | 298811,6 | 2361700,7 | 2021366,1 | 937789,1 | 362572,1 | 2838121,3 | 2406141,7 |
| P0C0L5 | Complement C4-B | CO4B | C4B | 141255,0 | 231135,8 | 68137,3 | 564957,7 | 718680,5 | 258672,5 | 201590,8 | 25519,1 | 72676,9 |
| P00450 | Ceruloplasmin | CERU | CP | 3931459,2 | 7149560,4 | 3936958,5 | 2398263,8 | 3053923,1 | 2560202,3 | 2009353,2 | 2357489,2 | 1042356,7 |
| P02751 | Fibronectin | FINC | FN1 | 721522,7 | 589513,6 | 594916,9 | 846710,2 | 1987259,6 | 2606103,2 | 1524657,3 | 2350349,9 | 1084902,6 |
| P01023 | Alpha-2-macroglobulin | A2MG | A2M | 10164,7 | 3314,1 | 1955,9 | 7169,8 | 15794,0 | 372288,0 | 3065,3 | 13303,0 | 43538,2 |
| P01031 | Complement C5 | CO5 | C5 | 244192,2 | 153334,1 | 95148,3 | 1167701,8 | 417978,5 | 543118,7 | 346502,4 | 973593,8 | 785438,3 |
| P02768 | Serum albumin | ALBU | ALB | 87504351,9 | 77375149,4 | 97471924,0 | 44563799,6 | 57590988,1 | 50493427,1 | 88960212,8 | 71966874,6 | 70157145,1 |
| P00747 | Plasminogen | PLMN | PLG | 68802,2 | 226373,4 | 94538,3 | 309885,8 | 240594,0 | 658674,9 | 214929,3 | 57352,4 | 146314,8 |
| P00751 | Complement factor B | CFAB | CFB | 716490,9 | 814314,9 | 323881,4 | 3710259,2 | 2731933,5 | 2371846,8 | 1488591,3 | 3820836,9 | 4900081,7 |
| P08603 | Complement factor H | CFAH | CFH | 541196,7 | 708920,5 | 295232,4 | 1687597,9 | 449529,7 | 2220572,6 | 1092700,0 | 517635,5 | 246436,9 |
| P02790 | Hemopexin | HEMO | HPX | 5183982,4 | 5273826,2 | 3548025,9 | 8536395,2 | 1691752,0 | 10424884,9 | 6072276,3 | 3695663,1 | 3869966,6 |
| P01024 | Complement C3 | CO3 | C3 | 1880,2 | 1092,1 | 1253,5 | 2520,7 | 3029,8 | 1648,2 | 729,6 | 2102,6 | 7904,5 |
| Q14624 | Inter-alpha-trypsin inhibitor heavy chain H4 | ITIH4 | ITIH4 | 751596,2 | 1066049,8 | 850928,9 | 2176916,3 | 1923319,1 | 1711221,7 | 963261,3 | 1498088,5 | 1844359,4 |
| P02787 | Serotransferrin | TRFE | TF | 2354,2 | 3237,9 | 2814,8 | 5013,4 | 1181,4 | 4525,8 | 2233,7 | 1449,3 | 2772,5 |
| P02774 | Vitamin D-binding protein | VTDB | GC | 4806044,6 | 7609110,2 | 5141652,9 | 2771301,7 | 1434902,2 | 2886373,7 | 1338000,0 | 1389902,6 | 577823,7 |
| P00734 | Prothrombin | THRB | F2 | 853162,8 | 1549672,3 | 694249,4 | 2139911,0 | 702283,2 | 2417540,4 | 1235182,7 | 773356,9 | 335711,0 |
| P19823 | Inter-alpha-trypsin inhibitor heavy chain H2 | ITIH2 | ITIH2 | 802331,2 | 748296,8 | 453180,2 | 2843819,2 | 3096351,8 | 1936406,3 | 1469331,7 | 2580752,9 | 1641489,2 |
| P06396 | Gelsolin | GELS | GSN | 310689,5 | 316029,7 | 305950,3 | 673059,9 | 1156582,8 | 1549828,8 | 394510,4 | 414814,7 | 404956,6 |
| P19827 | Inter-alpha-trypsin inhibitor heavy chain H1 | ITIH1 | ITIH1 | 568466,9 | 464281,3 | 320863,0 | 2041346,5 | 2220620,3 | 1957045,7 | 1222839,0 | 2170280,0 | 1726274,5 |
| P01011 | Alpha-1-antichymotrypsin | AACT | SERPINA3 | 2043886,9 | 2911896,0 | 1550599,4 | 8042517,4 | 10263273,0 | 5411924,7 | 3802572,8 | 6973167,7 | 7335298,7 |
| P01008 | Antithrombin-III | ANT3 | SERPINC1 | 941937,9 | 942182,6 | 818827,6 | 1856091,8 | 1858313,4 | 1366877,1 | 1186803,8 | 910255,4 | 533908,1 |
| P43652 | Afamin | AFAM | AFM | 491230,2 | 607983,9 | 372719,1 | 225299,5 | 385288,4 | 203901,2 | 422276,5 | 363772,9 | 239048,2 |
| P06727 | Apolipoprotein A-IV | APOA4 | APOA4 | 1613755,5 | 1568442,4 | 1317308,9 | 1332245,8 | 1594914,6 | 1345867,4 | 572555,5 | 437292,7 | 653927,7 |
| P01042 | Kininogen-1 | KNG1 | KNG1 | 1367257,7 | 620534,3 | 1000790,3 | 2322445,3 | 2820862,4 | 2112800,9 | 757507,8 | 432724,3 | 309781,1 |
| P13671 | Complement component C6 | CO6 | C6 | 81131,0 | 64268,3 | 41115,9 | 359948,4 | 114702,6 | 260317,9 | 135258,1 | 155406,0 | 107765,2 |
| P02671 | Fibrinogen alpha chain | FIBA | FGA | 2100,8 | 2699,7 | 1637,9 | 13683,6 | 9998,7 | 4316,5 | 2784,8 | 1701,2 | 6477,4 |
| P04217 | Alpha-1B-glycoprotein | A1BG | A1BG | 1816591,6 | 1883685,2 | 1108520,7 | 4306849,9 | 1864747,9 | 3599599,9 | 2558209,1 | 2136255,0 | 1203453,5 |
| P12259 | Coagulation factor V | FA5 | F5 | 33759,7 | 39770,8 | 18767,6 | 147415,9 | 143353,8 | 77274,1 | 40497,1 | 49561,4 | 112937,7 |
| P09871 | Complement C1s subcomponent | C1S | C1S | 130635,5 | 125120,2 | 54057,9 | 429164,9 | 302637,1 | 262264,8 | 195503,9 | 327656,5 | 233926,5 |
| P01019 | Angiotensinogen | ANGT | AGT | 999469,6 | 580424,0 | 556143,5 | 2849287,7 | 1732708,5 | 1398270,3 | 1778029,0 | 1952034,7 | 3120121,6 |
| P00736 | Complement C1r subcomponent | C1R | C1R | 253411,7 | 207148,0 | 127886,0 | 683450,2 | 238893,8 | 439523,8 | 382523,3 | 309203,7 | 138430,0 |
| P35908 | Keratin, type II cytoskeletal 2 epidermal | K22E | KRT2 | 25941,9 | 98987,9 | 52596,3 | 52656,3 | 334086,0 | 191575,4 | 8831,3 | 393444,2 | 811111,3 |
| P10643 | Complement component C7 | CO7 | C7 | 94932,5 | 134433,8 | 79633,5 | 449683,3 | 272372,4 | 622181,5 | 190899,0 | 154673,7 | 248962,8 |
| P05155 | Plasma protease C1 inhibitor | IC1 | SERPING1 | 989579,1 | 770142,6 | 756639,1 | 1852425,3 | 1725044,3 | 1715920,7 | 1713642,5 | 1697192,1 | 2661191,8 |
| P02749 | Beta-2-glycoprotein 1 | APOH | APOH | 946633,6 | 1779250,9 | 566038,9 | 869426,7 | 221958,2 | 579184,5 | 716724,2 | 146681,1 | 134705,6 |
| P04003 | C4b-binding protein alpha chain | C4BPA | C4BPA | 693160,7 | 502264,1 | 218521,7 | 2342661,4 | 1043021,1 | 1825363,6 | 227000,7 | 654977,4 | 370104,6 |
| P08697 | Alpha-2-antiplasmin | A2AP | SERPINF2 | 826648,4 | 777022,2 | 633587,8 | 1586953,4 | 1434549,5 | 1087891,5 | 1203632,1 | 699430,5 | 926245,8 |
| P02748 | Complement component C9 | CO9 | C9 | 385484,1 | 573225,1 | 323673,8 | 1265308,1 | 1328847,0 | 1492882,2 | 488771,5 | 620560,4 | 351179,5 |
| P02765 | Alpha-2-HS-glycoprotein | FETUA | AHSG | 311769,0 | 545944,2 | 265766,7 | 194762,8 | 74573,7 | 199655,4 | 221947,3 | 58862,8 | 128124,2 |
| P04264 | Keratin, type II cytoskeletal 1 | K2C1 | KRT1 | 702703,9 | 1213319,9 | 476748,5 | 388508,6 | 2945984,9 | 1455522,5 | 113101,7 | 2865406,0 | 6956591,8 |
| P04275 | von Willebrand factor | VWF | VWF | 18204,9 | 5567,2 | 11032,1 | 55612,1 | 40942,9 | 44916,8 | 4014,2 | 92325,7 | 38079,9 |
| P02647 | Apolipoprotein A-I | APOA1 | APOA1 | 1658,9 | 1647,1 | 1662,9 | 1298,8 | 1883,9 | 1340,8 | 553,2 | 1321,6 | 5140,9 |
| P35527 | Keratin, type I cytoskeletal 9 | K1C9 | KRT9 | 318094,4 | 327021,8 | 312327,9 | 113392,3 | 603905,3 | 526839,4 | 70847,6 | 592430,4 | 1474119,6 |
| Q06033 | Inter-alpha-trypsin inhibitor heavy chain H3 | ITIH3 | ITIH3 | 121846,6 | 180904,6 | 257756,5 | 362539,2 | 527461,3 | 546280,8 | 142934,9 | 728004,5 | 403760,1 |
| P06681 | Complement C2 | CO2 | C2 | 151806,6 | 114705,6 | 63413,5 | 705953,2 | 449962,0 | 320689,2 | 324011,9 | 464577,4 | 644751,7 |
| Q9Y6R7 | IgGFc-binding protein | FCGBP | FCGBP | 50085,5 | 57926,9 | 49586,4 | 66998,2 | 107649,2 | 98857,1 | 55433,4 | 107165,3 | 126474,2 |
| P03952 | Plasma kallikrein | KLKB1 | KLKB1 | 140884,2 | 213385,9 | 133900,2 | 349101,5 | 221004,7 | 329335,8 | 211786,4 | 192976,8 | 151117,9 |
| P07358 | Complement component C8 beta chain | CO8B | C8B | 139981,6 | 113118,5 | 26791,5 | 514273,3 | 456802,0 | 249948,9 | 138312,2 | 446614,0 | 260030,7 |
| P00738 | Haptoglobin | HPT | HP | 5338,3 | 7134,3 | 6450,9 | 5772,0 | 5685,1 | 11152,2 | 6318,3 | 2686,6 | 8075,1 |
| P04004 | Vitronectin | VTNC | VTN | 206534,5 | 220936,8 | 151227,2 | 304623,4 | 153295,3 | 446937,7 | 141716,5 | 239799,5 | 282132,3 |
| P13645 | Keratin, type I cytoskeletal 10 | K1C10 | KRT10 | 129487,4 | 276261,2 | 102611,7 | 109736,3 | 436605,5 | 436136,1 | 44075,2 | 641783,6 | 1113483,4 |
| P02675 | Fibrinogen beta chain | FIBB | FGB | 1348,6 | 1486,6 | 1379,7 | 5195,8 | 3235,8 | 3329,1 | 644,5 | 2105,1 | 7222,5 |
| P05156 | Complement factor I | CFAI | CFI | 95228,8 | 133057,7 | 78315,6 | 346169,5 | 304974,6 | 291460,6 | 201811,6 | 269589,3 | 305955,3 |
| P05546 | Heparin cofactor 2 | HEP2 | SERPIND1 | 188231,0 | 169287,6 | 93359,7 | 607713,7 | 579965,8 | 523611,2 | 270395,0 | 1009879,4 | 318310,7 |
| P00488 | Coagulation factor XIII A chain | F13A | F13A1 | 36313,6 | 61226,3 | 17517,3 | 125452,3 | 180898,2 | 127713,3 | 69735,1 | 229056,6 | 261872,5 |
| P08519 | Apolipoprotein(a) | APOA | LPA | 2126,9 | 1816,7 | 1264,0 | 1408,0 | 1533,3 | 5513,4 | 486,5 | 2389,8 | 3692,2 |
| P05160 | Coagulation factor XIII B chain | F13B | F13B | 103316,1 | 70829,8 | 101249,0 | 203147,1 | 115218,0 | 197065,0 | 153646,3 | 105217,1 | 162107,7 |
| Q96PD5 | N-acetylmuramoyl-L-alanine amidase | PGRP2 | PGLYRP2 | 352680,0 | 398291,8 | 177721,8 | 889657,4 | 550199,2 | 403376,9 | 365587,5 | 470059,4 | 182445,1 |
| O75882 | Attractin | ATRN | ATRN | 13996,9 | 17282,3 | 13978,1 | 12425,6 | 3510,3 | 28344,5 | 15631,0 | 17298,7 | 23721,6 |
| P20742 | Pregnancy zone protein | PZP | PZP | 341750,5 | 403536,2 | 250244,8 | 1897100,6 | 540483,6 | 2397125,7 | 775111,5 | 740827,4 | 1318209,3 |
| P04196 | Histidine-rich glycoprotein | HRG | HRG | 1382807,3 | 1086965,1 | 1683878,2 | 476897,3 | 1252233,2 | 1004384,7 | 774105,7 | 314562,8 | 338303,7 |
| P00748 | Coagulation factor XII | FA12 | F12 | 205666,2 | 215892,3 | 114033,4 | 624389,1 | 633452,4 | 532868,6 | 349746,1 | 364359,5 | 225450,3 |
| P36955 | Pigment epithelium-derived factor | PEDF | SERPINF1 | 271294,9 | 194813,0 | 160059,1 | 637998,0 | 550407,1 | 408924,6 | 153691,7 | 523921,8 | 443220,3 |
| P26927 | Hepatocyte growth factor-like protein | HGFL | MST1 | 13159,8 | 10064,3 | 11963,0 | 41658,5 | 26989,2 | 21671,9 | 7100,5 | 16343,8 | 58821,9 |
| P01009 | Alpha-1-antitrypsin | A1AT | SERPINA1 | 2447,8 | 2294,3 | 3116,4 | 5671,2 | 5154,8 | 5130,9 | 510,8 | 2415,1 | 3845,8 |
| P15924 | Desmoplakin | DESP | DSP | 8750,0 | 9774,6 | 7240,9 | 11784,2 | 24171,4 | 10592,4 | 4689,6 | 8748,5 | 25870,3 |
| P29622 | Kallistatin | KAIN | SERPINA4 | 156894,5 | 81749,0 | 63472,0 | 419527,4 | 326147,6 | 240920,5 | 242887,8 | 338387,9 | 242023,0 |
| P10909 | Clusterin | CLUS | CLU | 246209,8 | 483528,8 | 315536,5 | 407555,2 | 583349,2 | 406683,3 | 211291,1 | 158813,7 | 58488,3 |
| P02760 | Protein AMBP | AMBP | AMBP | 197929,2 | 256481,4 | 195240,0 | 361413,2 | 70195,8 | 308808,7 | 111081,2 | 144150,3 | 168363,1 |
| Q16610 | Extracellular matrix protein 1 | ECM1 | ECM1 | 19312,9 | 19159,7 | 16690,2 | 20801,9 | 29453,2 | 29658,9 | 8325,1 | 22088,2 | 53868,6 |
| P02538 | Keratin, type II cytoskeletal 6A | K2C6A | KRT6A | 5860,3 | 16035,5 | 1713,0 | 3010,8 | 11964,1 | 11311,4 | 3452,9 | 10339,8 | 17505,0 |
| P07225 | Vitamin K-dependent protein S | PROS | PROS1 | 162684,8 | 143087,3 | 85756,3 | 484001,5 | 390869,1 | 291574,9 | 300141,7 | 238521,7 | 62577,5 |
| P07357 | Complement component C8 alpha chain | CO8A | C8A | 18729,2 | 23487,0 | 14054,6 | 69386,6 | 14642,7 | 55831,1 | 39098,6 | 25460,7 | 52462,5 |
| P25311 | Zinc-alpha-2-glycoprotein | ZA2G | AZGP1 | 41268,5 | 24589,2 | 17886,6 | 32647,4 | 33702,8 | 27451,9 | 16289,9 | 31091,6 | 139458,8 |
| P02679 | Fibrinogen gamma chain | FIBG | FGG | 2172,4 | 1772,7 | 2217,7 | 14661,2 | 10567,5 | 7206,3 | 3733,4 | 3172,1 | 3830,2 |
| P0DOX5 | Immunoglobulin gamma-1 heavy chain | IGG1 | 1 SV | 5998,8 | 4079,0 | 3741,8 | 8272,1 | 6805,3 | 6100,4 | 686,5 | 2841,6 | 7350,2 |
| P35858 | Insulin-like growth factor-binding protein complex acid labile subunit | ALS | IGFALS | 226765,8 | 152668,7 | 111309,3 | 728266,5 | 438735,0 | 184579,4 | 443674,1 | 782640,2 | 859363,1 |
| O00391 | Sulfhydryl oxidase 1 | QSOX1 | QSOX1 | 92033,2 | 90782,7 | 56023,2 | 120775,9 | 113254,6 | 107534,8 | 47287,8 | 70912,3 | 101837,9 |
| P02649 | Apolipoprotein E | APOE | APOE | 153518,5 | 157268,6 | 329038,7 | 271686,4 | 298181,0 | 575583,9 | 124009,3 | 249559,4 | 187115,9 |
| P22792 | Carboxypeptidase N subunit 2 | CPN2 | CPN2 | 200180,6 | 265681,3 | 136545,8 | 520373,1 | 371534,5 | 407188,6 | 374185,4 | 266086,5 | 120799,2 |
| P08779 | Keratin, type I cytoskeletal 16 | K1C16 | KRT16 | 71125,5 | 228531,4 | 33861,7 | 19957,8 | 94681,4 | 133977,2 | 23671,9 | 99065,5 | 307929,2 |
| P03951 | Coagulation factor XI | FA11 | F11 | 26146,9 | 13351,0 | 16582,8 | 32753,2 | 16519,7 | 18817,0 | 5393,5 | 20020,8 | 22784,6 |
| P01871 | Immunoglobulin heavy constant mu | IGHM | IGHM | 411530,4 | 40451,2 | 52482,3 | 378573,9 | 39184,5 | 199506,0 | 2573,3 | 125969,6 | 12297,6 |
| Q14520 | Hyaluronan-binding protein 2 | HABP2 | HABP2 | 12397,9 | 23048,4 | 10618,5 | 41009,6 | 21205,7 | 49363,6 | 30626,8 | 14753,3 | 16648,0 |
| P13796 | Plastin-2 | PLSL | LCP1 | 21145,4 | 39453,7 | 31855,3 | 298577,4 | 392025,2 | 149690,0 | 47511,3 | 85462,5 | 168704,8 |
| P05543 | Thyroxine-binding globulin | THBG | SERPINA7 | 157945,6 | 304039,8 | 183203,4 | 542320,3 | 641019,1 | 493329,1 | 163532,8 | 541034,7 | 668351,8 |
| Q92954 | Proteoglycan 4 | PRG4 | PRG4 | 36825,5 | 45156,5 | 15544,7 | 92974,8 | 177658,1 | 64875,8 | 47075,0 | 183939,4 | 94671,6 |
| P22105 | Tenascin-X | TENX | TNXB | 38115,6 | 63674,7 | 46778,5 | 48222,9 | 69176,1 | 32077,5 | 9222,1 | 66177,7 | 43238,4 |
| P02753 | Retinol-binding protein 4 | RET4 | RBP4 | 139328,2 | 96299,3 | 125683,6 | 65478,4 | 28067,7 | 78065,6 | 11875,0 | 49273,7 | 56157,5 |
| P80108 | Phosphatidylinositol-glycan-specific phospholipase D | PHLD | GPLD1 | 115204,2 | 129974,4 | 58695,9 | 298745,5 | 364812,7 | 133481,5 | 151133,8 | 243541,8 | 258109,9 |
| P06276 | Cholinesterase | CHLE | BCHE | 77127,3 | 58168,6 | 40899,7 | 134478,4 | 104960,4 | 109992,0 | 41283,5 | 78921,7 | 77934,9 |
| P05452 | Tetranectin | TETN | CLEC3B | 52139,3 | 87970,7 | 89241,5 | 73810,8 | 114729,6 | 137302,1 | 30372,1 | 81404,0 | 101491,1 |
| Q96KN2 | Beta-Ala-His dipeptidase | CNDP1 | CNDP1 | 51235,0 | 38102,6 | 39922,8 | 106112,7 | 83092,7 | 93065,6 | 46109,9 | 48766,8 | 173425,4 |
| P13647 | Keratin, type II cytoskeletal 5 | K2C5 | KRT5 | 436,1 | 2644,0 | 515,7 | 2155,8 | 4996,5 | 2839,3 | 847,4 | 3593,0 | 15069,6 |
| P51884 | Lumican | LUM | LUM | 548989,0 | 797157,9 | 644662,5 | 568664,3 | 834162,1 | 530295,3 | 311647,8 | 105707,2 | 77598,5 |
| Q04756 | Hepatocyte growth factor activator | HGFA | HGFAC | 39640,5 | 59138,5 | 35341,4 | 28995,4 | 52009,4 | 47115,0 | 39553,5 | 35791,9 | 80199,9 |
| Q9UK55 | Protein Z-dependent protease inhibitor | ZPI | SERPINA10 | 102067,4 | 90631,2 | 70504,5 | 222812,8 | 218263,7 | 154432,4 | 111950,9 | 190911,6 | 245182,4 |
| Q96IY4 | Carboxypeptidase B2 | CBPB2 | CPB2 | 214423,7 | 254903,9 | 159168,9 | 258868,9 | 310818,5 | 189591,2 | 173586,3 | 271372,6 | 144386,5 |
| P02746 | Complement C1q subcomponent subunit B | C1QB | C1QB | 100099,4 | 67020,1 | 44084,0 | 363933,7 | 247620,8 | 221720,1 | 50744,0 | 99662,5 | 36495,8 |
| P00742 | Coagulation factor X | FA10 | F10 | 189193,1 | 273793,1 | 118555,6 | 244767,1 | 182297,9 | 55146,2 | 123950,8 | 185981,2 | 114601,3 |
| P02750 | Leucine-rich alpha-2-glycoprotein | A2GL | LRG1 | 726725,1 | 1399082,6 | 884150,9 | 1775685,9 | 2594170,0 | 1409275,4 | 394790,6 | 197808,6 | 409884,1 |
| P48740 | Mannan-binding lectin serine protease 1 | MASP1 | MASP1 | 4698,9 | 20202,8 | 4387,3 | 24472,2 | 25725,6 | 21201,1 | 13485,1 | 12937,1 | 46713,7 |
| P14923 | Junction plakoglobin | PLAK | JUP | 11125,7 | 16956,8 | 5041,1 | 3855,3 | 32786,9 | 19152,0 | 3224,6 | 34075,6 | 81569,1 |
| P02743 | Serum amyloid P-component | SAMP | APCS | 997813,8 | 1284347,8 | 736444,4 | 981579,5 | 820301,3 | 507761,8 | 428123,8 | 1338539,8 | 64242,5 |
| P08185 | Corticosteroid-binding globulin | CBG | SERPINA6 | 144027,6 | 126052,6 | 111039,2 | 295677,2 | 288378,2 | 289196,7 | 166105,9 | 71273,9 | 109268,8 |
| Q15582 | Transforming growth factor-beta-induced protein ig-h3 | BGH3 | TGFBI | 32917,3 | 29562,4 | 22435,1 | 80416,9 | 109767,6 | 56451,6 | 23164,7 | 87692,0 | 125689,0 |
| P07360 | Complement component C8 gamma chain | CO8G | C8G | 86699,7 | 83170,1 | 48297,7 | 307501,7 | 321705,3 | 277246,5 | 28355,1 | 362220,0 | 261536,5 |
| P23142 | Fibulin-1 | FBLN1 | FBLN1 | 37076,7 | 57081,1 | 36067,2 | 124432,1 | 106119,8 | 163590,0 | 69579,2 | 54044,8 | 77781,6 |
| Q86YZ3 | Hornerin | HORN | HRNR | 252,6 | 253,4 | 290,0 | 235,6 | 472,8 | 320,2 | 145,1 | 367,5 | 1369,3 |
| P27169 | Serum paraoxonase/arylesterase 1 | PON1 | PON1 | 882,1 | 4182,8 | 9432,4 | 6458,5 | 1491,3 | 1643,0 | 1798,6 | 1783,4 | 3972,7 |
| P60709 | Actin, cytoplasmic 1 | ACTB | ACTB | 37847,7 | 9778,4 | 8762,3 | 87611,3 | 92207,4 | 230903,2 | 21218,5 | 55849,5 | 94081,1 |
| P05154 | Plasma serine protease inhibitor | IPSP | SERPINA5 | 142523,4 | 159549,6 | 222842,9 | 194347,7 | 230891,1 | 121537,8 | 116327,7 | 171157,5 | 182811,9 |
| P49747 | Cartilage oligomeric matrix protein | COMP | COMP | 13133,6 | 26450,3 | 10279,1 | 11817,2 | 21832,3 | 6458,7 | 4772,2 | 8221,9 | 35101,4 |
| P15144 | Aminopeptidase N | AMPN | ANPEP | 22808,5 | 12423,1 | 16335,1 | 14720,2 | 67189,8 | 57536,3 | 24068,5 | 100550,9 | 356057,3 |
| P01876 | Immunoglobulin heavy constant alpha 1 | IGHA1 | IGHA1 | 126,6 | 113,3 | 94,2 | 229,7 | 280,9 | 162,2 | 143,3 | 310,9 | 955,0 |
| Q04695 | Keratin, type I cytoskeletal 17 | K1C17 | KRT17 | 7803,8 | 24813,3 | 2607,0 | 5413,6 | 12852,0 | 4712,0 | 2747,8 | 23351,2 | 55203,9 |
| Q12805 | EGF-containing fibulin-like extracellular matrix protein 1 | FBLN3 | EFEMP1 | 13111,1 | 15423,9 | 4800,9 | 29392,3 | 40446,9 | 30484,3 | 1724,3 | 32887,8 | 43754,7 |
| P43251 | Biotinidase | BTD | BTD | 46953,2 | 34719,7 | 27511,9 | 110774,2 | 60156,6 | 100982,0 | 81109,2 | 55633,8 | 73643,0 |
| P12111 | Collagen alpha-3(VI) chain | CO6A3 | COL6A3 | 35576,2 | 18944,1 | 64677,7 | 29601,9 | 29273,6 | 22888,8 | 4447,6 | 39540,3 | 58919,0 |
| P22352 | Glutathione peroxidase 3 | GPX3 | GPX3 | 41275,0 | 94914,1 | 84454,8 | 73332,7 | 73103,6 | 9430,8 | 6494,7 | 10740,3 | 31913,9 |
| Q9BXR6 | Complement factor H-related protein 5 | FHR5 | CFHR5 | 115,7 | 248,7 | 298,5 | 355,7 | 632,8 | 628,1 | 419,6 | 298,9 | 2035,2 |
| P04278 | Sex hormone-binding globulin | SHBG | SHBG | 186631,1 | 241346,5 | 182065,0 | 280091,8 | 761628,9 | 377405,9 | 94532,7 | 382302,3 | 311288,2 |
| P0C0L4 | Complement C4-A | CO4A | C4A | 392321,9 | 465240,7 | 259750,1 | 1345201,9 | 1354259,6 | 946588,7 | 542562,4 | 705569,3 | 508083,6 |
| P15169 | Carboxypeptidase N catalytic chain | CBPN | CPN1 | 81240,7 | 77927,1 | 58249,2 | 198843,2 | 233734,5 | 189602,7 | 89133,6 | 228969,8 | 185116,3 |
| P18428 | Lipopolysaccharide-binding protein | LBP | LBP | 104833,1 | 89802,1 | 48822,0 | 596195,3 | 535300,1 | 307358,1 | 64692,8 | 284436,8 | 259917,2 |
| Q9UGM5 | Fetuin-B | FETUB | FETUB | 32391,6 | 46687,1 | 8544,6 | 49941,1 | 14963,5 | 21441,3 | 29085,1 | 8263,5 | 45510,1 |
| P00740 | Coagulation factor IX | FA9 | F9 | 21015,1 | 15474,7 | 14943,6 | 56586,7 | 37144,5 | 62305,0 | 40324,1 | 45325,6 | 86634,9 |
| P0DOX8 | Immunoglobulin lambda-1 light chain | IGL1 | 1 SV | 390,4 | 237,5 | 1367,9 | 2314,5 | 1851,2 | 3860,4 | 1122,8 | 286,4 | 806,8 |
| O00533 | Neural cell adhesion molecule L1-like protein | NCHL1 | CHL1 | 4142,7 | 2370,8 | 7358,5 | 19945,4 | 17731,2 | 26607,3 | 4295,5 | 5041,9 | 31648,4 |
| P22891 | Vitamin K-dependent protein Z | PROZ | PROZ | 9027,8 | 7285,4 | 865,0 | 15054,1 | 8955,4 | 9877,3 | 12248,2 | 6510,6 | 20878,3 |
| P68871 | Hemoglobin subunit beta | HBB | HBB | 261,0 | 575,9 | 190,1 | 230,8 | 207,4 | 414,5 | 114,7 | 133,1 | 1770,8 |
| Q08380 | Galectin-3-binding protein | LG3BP | LGALS3BP | 4664,5 | 14173,4 | 2797,1 | 22926,3 | 2456,1 | 18708,4 | 1089,1 | 10342,3 | 17302,0 |
| Q76LX8 | A disintegrin and metalloproteinase with thrombospondin motifs 13 | ATS13 | ADAMTS13 | 66902,6 | 25811,7 | 25544,5 | 11788,6 | 24619,8 | 31882,5 | 6767,2 | 11997,3 | 21064,6 |
| P17936 | Insulin-like growth factor-binding protein 3 | IBP3 | IGFBP3 | 1717,9 | 5671,3 | 1570,2 | 8596,1 | 12598,6 | 2594,2 | 2211,4 | 2655,5 | 11153,4 |
| O95445 | Apolipoprotein M | APOM | APOM | 21216,2 | 22457,2 | 19041,7 | 21273,1 | 25668,0 | 17062,4 | 11555,5 | 6507,5 | 44567,2 |
| Q9NZP8 | Complement C1r subcomponent-like protein | C1RL | C1RL | 38237,5 | 77507,7 | 39155,9 | 71525,4 | 139285,2 | 81769,3 | 42134,6 | 40648,3 | 78840,1 |
| Q13822 | Ectonucleotide pyrophosphatase/phosphodiesterase family member 2 | ENPP2 | ENPP2 | 450,0 | 59,9 | 78,8 | 232,3 | 273,4 | 168,3 | 96,5 | 186,6 | 731,9 |
| P08571 | Monocyte differentiation antigen CD14 | CD14 | CD14 | 39050,2 | 45696,8 | 28174,0 | 183901,5 | 269603,1 | 142075,0 | 36989,1 | 128371,1 | 76458,4 |
| P02747 | Complement C1q subcomponent subunit C | C1QC | C1QC | 11013,0 | 4389,6 | 3641,7 | 9491,2 | 16890,4 | 3487,8 | 6343,7 | 15339,4 | 24136,2 |
| P01859 | Immunoglobulin heavy constant gamma 2 | IGHG2 | IGHG2 | 176,7 | 165,6 | 243,8 | 199,4 | 417,0 | 500,1 | 143,4 | 279,7 | 2112,4 |
| P35443 | Thrombospondin-4 | TSP4 | THBS4 | 4523,3 | 5148,0 | 3727,7 | 7861,1 | 4053,2 | 7160,6 | 6180,6 | 19295,6 | 28840,8 |
| Q5T749 | Keratinocyte proline-rich protein | KPRP | KPRP | 2786,1 | 2038,5 | 3439,6 | 1573,8 | 1476,2 | 4434,5 | 3973,5 | 2064,4 | 16938,2 |
| P02766 | Transthyretin | TTHY | TTR | 246,3 | 540,0 | 243,9 | 378,2 | 539,4 | 776,5 | 578,5 | 881,8 | 3557,4 |
| O00187 | Mannan-binding lectin serine protease 2 | MASP2 | MASP2 | 1501,7 | 1955,8 | 1788,5 | 3337,0 | 731,0 | 1889,1 | 2055,9 | 1163,0 | 2885,1 |
| Q9UHG3 | Prenylcysteine oxidase 1 | PCYOX | PCYOX1 | 26966,7 | 19799,3 | 24726,9 | 26796,4 | 43519,6 | 41706,4 | 32928,8 | 57903,1 | 56680,7 |
| P02652 | Apolipoprotein A-II | APOA2 | APOA2 | 307319,2 | 115008,5 | 163902,5 | 104119,1 | 234713,0 | 58207,4 | 52015,8 | 177635,8 | 166763,4 |
| P00746 | Complement factor D | CFAD | CFD | 46945,5 | 51754,2 | 53929,8 | 58755,6 | 73416,5 | 55628,5 | 7357,4 | 18765,5 | 49632,3 |
| Q86VB7 | Scavenger receptor cysteine-rich type 1 protein M130 | C163A | CD163 | 1007,1 | 434,6 | 212,2 | 225,8 | 929,4 | 356,4 | 219,4 | 994,7 | 5139,0 |
| P33908 | Mannosyl-oligosaccharide 1,2-alpha-mannosidase IA | MA1A1 | MAN1A1 | 8669,9 | 7686,9 | 4677,7 | 17038,2 | 16457,2 | 9127,6 | 8124,1 | 12611,2 | 34733,6 |
| P19013 | Keratin, type II cytoskeletal 4 | K2C4 | KRT4 | 4110,5 | 1556,5 | 782,5 | 3964,3 | 34039,5 | 2427,9 | 2769,3 | 5944,6 | 42896,1 |
| O43866 | CD5 antigen-like | CD5L | CD5L | 3982,3 | 9089,1 | 5249,5 | 7366,6 | 13095,2 | 16512,7 | 5431,8 | 2494,9 | 9442,5 |
| P32119 | Peroxiredoxin-2 | PRDX2 | PRDX2 | 136045,9 | 61674,5 | 51161,5 | 5550,4 | 7852,0 | 9998,5 | 4648,0 | 16069,4 | 4272,3 |
| P02533 | Keratin, type I cytoskeletal 14 | K1C14 | KRT14 | 15602,5 | 22031,8 | 15172,1 | 11317,7 | 23242,4 | 17247,2 | 8462,9 | 11238,0 | 89535,1 |
| O75636 | Ficolin-3 | FCN3 | FCN3 | 35661,9 | 68195,1 | 21310,1 | 139318,2 | 17025,9 | 151679,3 | 4495,7 | 34854,2 | 56073,3 |
| P27918 | Properdin | PROP | CFP | 4990,6 | 11614,0 | 3008,1 | 13109,7 | 2446,7 | 12064,4 | 7820,9 | 1957,6 | 5528,2 |
| P02745 | Complement C1q subcomponent subunit A | C1QA | C1QA | 11118,9 | 2452,6 | 3365,7 | 4015,1 | 6021,0 | 7472,4 | 3188,6 | 7355,4 | 12837,2 |
| P49908 | Selenoprotein P | SEPP1 | SELENOP | 2620,7 | 26661,9 | 34420,4 | 31217,1 | 15147,9 | 14250,2 | 38870,4 | 15475,4 | 35687,8 |
| P55058 | Phospholipid transfer protein | PLTP | PLTP | 11344,1 | 16439,9 | 13077,2 | 25869,2 | 29387,4 | 43982,0 | 22013,5 | 41321,4 | 58101,9 |
| Q07954 | Prolow-density lipoprotein receptor-related protein 1 | LRP1 | LRP1 | 678,4 | 834,8 | 724,6 | 1202,8 | 1690,6 | 757,2 | 286,3 | 5396,6 | 7285,2 |
| P13591 | Neural cell adhesion molecule 1 | NCAM1 | NCAM1 | 1880,5 | 540,0 | 401,5 | 2613,4 | 1994,6 | 1509,8 | 425,2 | 3424,7 | 3306,1 |
| P33151 | Cadherin-5 | CADH5 | CDH5 | 13046,3 | 16128,7 | 3959,2 | 14652,7 | 35072,1 | 33977,6 | 10575,5 | 16570,3 | 20842,1 |
| P07359 | Platelet glycoprotein Ib alpha chain | GP1BA | GP1BA | 13531,4 | 15534,0 | 11121,4 | 21534,1 | 12513,7 | 3778,0 | 3944,9 | 12305,0 | 13722,3 |
| O14791 | Apolipoprotein L1 | APOL1 | APOL1 | 50,7 | 75,6 | 70,8 | 100,9 | 156,7 | 108,9 | 57,4 | 149,6 | 459,3 |
| P02656 | Apolipoprotein C-III | APOC3 | APOC3 | 493907,5 | 346211,8 | 518062,2 | 141962,6 | 271947,8 | 161607,2 | 154428,9 | 70138,5 | 39693,0 |
| P08709 | Coagulation factor VII | FA7 | F7 | 11965,1 | 17753,8 | 26998,6 | 27341,0 | 57793,8 | 22598,5 | 16481,0 | 23295,7 | 61422,3 |
| P13646 | Keratin, type I cytoskeletal 13 | K1C13 | KRT13 | 244,9 | 919,8 | 1267,7 | 228,3 | 241,3 | 478,2 | 85,6 | 1160,8 | 1553,7 |
| Q8NBP7 | Proprotein convertase subtilisin/kexin type 9 | PCSK9 | PCSK9 | 833,0 | 1833,3 | 865,5 | 11773,7 | 17293,6 | 5392,5 | 2119,6 | 2939,9 | 12180,1 |
| Q6EMK4 | Vasorin | VASN | VASN | 1168,2 | 6384,8 | 1714,0 | 18545,2 | 21870,2 | 10145,1 | 12092,8 | 19017,8 | 26958,1 |
| P04070 | Vitamin K-dependent protein C | PROC | PROC | 9925,4 | 21696,2 | 17345,0 | 109799,5 | 32245,4 | 57790,5 | 20193,0 | 9457,7 | 22089,6 |
| P20851 | C4b-binding protein beta chain | C4BPB | C4BPB | 27282,9 | 16104,4 | 13562,4 | 49937,6 | 3030,6 | 37847,1 | 4721,9 | 9324,9 | 10666,1 |
| P54802 | Alpha-N-acetylglucosaminidase | ANAG | NAGLU | 7191,4 | 1001,5 | 9530,4 | 545,0 | 3454,3 | 584,6 | 448,6 | 13779,3 | 3730,1 |
| P19320 | Vascular cell adhesion protein 1 | VCAM1 | VCAM1 | 1570,1 | 3601,9 | 1179,6 | 11910,2 | 19108,5 | 17600,1 | 421,9 | 10853,0 | 13583,1 |
| P05090 | Apolipoprotein D | APOD | APOD | 9058,1 | 14301,7 | 13203,6 | 1992,4 | 9773,3 | 12331,1 | 6932,8 | 1870,9 | 7992,8 |
| P04180 | Phosphatidylcholine-sterol acyltransferase | LCAT | LCAT | 54525,4 | 49979,7 | 22630,2 | 60223,9 | 22633,4 | 25368,4 | 19319,3 | 36715,4 | 19977,6 |
| P04040 | Catalase | CATA | CAT | 27529,8 | 11336,4 | 6066,6 | 6207,4 | 14113,1 | 11726,1 | 9158,2 | 10097,6 | 24906,5 |
| P61626 | Lysozyme C | LYSC | LYZ | 18736,3 | 26856,5 | 18439,3 | 59625,9 | 19229,8 | 100887,3 | 9901,0 | 18134,3 | 63155,4 |
| P01880 | Immunoglobulin heavy constant delta | IGHD | IGHD | 14203,4 | 20603,3 | 79034,9 | 30168,9 | 20100,0 | 333966,0 | 12693,8 | 22676,4 | 70133,4 |
| P07355 | Annexin A2 | ANXA2 | ANXA2 | 566,2 | 7506,9 | 6912,9 | 6082,1 | 13917,3 | 4713,4 | 3565,0 | 5809,6 | 56066,1 |
| O95497 | Pantetheinase | VNN1 | VNN1 | 2491,4 | 1293,8 | 1106,0 | 981,7 | 4859,9 | 3879,3 | 2035,0 | 2389,5 | 8643,6 |
| P07996 | Thrombospondin-1 | TSP1 | THBS1 | 24088,1 | 17742,2 | 14209,8 | 64049,1 | 54706,6 | 29601,3 | 13770,3 | 89584,9 | 138044,1 |
| P02741 | C-reactive protein | CRP | CRP | 60052,8 | 33135,6 | 52041,9 | 720980,5 | 194007,4 | 239713,6 | 42528,2 | 38394,9 | 76835,8 |
| Q8N1N4 | Keratin, type II cytoskeletal 78 | K2C78 | KRT78 | 1370,8 | 9595,4 | 5875,3 | 3234,9 | 36634,5 | 97102,3 | 9006,4 | 16787,6 | 153871,6 |
| P18206 | Vinculin | VINC | VCL | 2540,3 | 1461,2 | 2019,6 | 15035,9 | 90845,5 | 25712,8 | 34323,1 | 75939,7 | 12853,1 |
| P12955 | Xaa-Pro dipeptidase | PEPD | PEPD | 3940,5 | 6656,2 | 1318,8 | 3301,7 | 2700,4 | 3518,2 | 2490,9 | 5593,6 | 31033,2 |
| P35542 | Serum amyloid A-4 protein | SAA4 | SAA4 | 338635,6 | 236897,7 | 162957,1 | 170590,8 | 29829,6 | 114104,8 | 10360,7 | 162147,1 | 112244,7 |
| P69905 | Hemoglobin subunit alpha | HBA | HBA1 | 96981,5 | 119610,8 | 48319,2 | 6415,2 | 365,8 | 4641,5 | 290,0 | 1624,3 | 1621,4 |
| P01619 | Immunoglobulin kappa variable 3-20 | KV320 | IGKV3-20 | 146,5 | 223,2 | 204,3 | 297,8 | 399,8 | 255,6 | 214,0 | 500,4 | 1762,8 |
| P02655 | Apolipoprotein C-II | APOC2 | APOC2 | 105270,9 | 63332,0 | 116186,9 | 36375,1 | 56949,2 | 59385,8 | 37483,0 | 10717,0 | 41803,6 |
| Q15113 | Procollagen C-endopeptidase enhancer 1 | PCOC1 | PCOLCE | 116,8 | 75,6 | 268,1 | 222,2 | 104,7 | 131,5 | 57,3 | 186,6 | 308,7 |
| Q13093 | Platelet-activating factor acetylhydrolase | PAFA | PLA2G7 | 474,8 | 6625,2 | 972,5 | 1747,8 | 7220,4 | 2293,5 | 3174,2 | 6886,1 | 6134,6 |
| Q5D862 | Filaggrin-2 | FILA2 | FLG2 | 3235,0 | 13105,5 | 2269,5 | 4139,3 | 35859,0 | 17558,7 | 1061,5 | 27133,8 | 123428,1 |
| P14151 | L-selectin | LYAM1 | SELL | 4475,8 | 22256,6 | 14966,0 | 32338,5 | 27879,7 | 38677,2 | 4189,2 | 15856,1 | 22735,7 |
| Q16706 | Alpha-mannosidase 2 | MA2A1 | MAN2A1 | 5185,1 | 2837,0 | 4132,7 | 7768,8 | 8245,7 | 3324,2 | 2597,8 | 4441,0 | 28684,1 |
| Q7Z794 | Keratin, type II cytoskeletal 1b | K2C1B | KRT77 | 4925,7 | 1841,6 | 2909,7 | 4147,1 | 12100,4 | 8568,3 | 1450,5 | 3987,1 | 78748,2 |
| Q12860 | Contactin-1 | CNTN1 | CNTN1 | 2979,2 | 961,2 | 1878,3 | 1836,5 | 2444,6 | 5658,9 | 32528,7 | 11429,6 | 4616,8 |
| P0DOY3 | Immunoglobulin lambda constant 3 | IGLC3 | IGLC3 | 2378,3 | 584,7 | 2409,5 | 781,6 | 3490,9 | 4139,5 | 3008,3 | 716,8 | 2313,6 |
| P80188 | Neutrophil gelatinase-associated lipocalin | NGAL | LCN2 | 3753,3 | 8711,8 | 18336,0 | 28388,6 | 7370,8 | 19730,0 | 2420,3 | 17132,3 | 32255,5 |
| P54108 | Cysteine-rich secretory protein 3 | CRIS3 | CRISP3 | 5731,3 | 17746,2 | 13762,7 | 2548,3 | 3905,0 | 4926,3 | 4564,1 | 5907,6 | 19205,0 |
| Q9UNW1 | Multiple inositol polyphosphate phosphatase 1 | MINP1 | MINPP1 | 12254,7 | 21804,6 | 4663,6 | 1582,1 | 11764,1 | 5298,3 | 818,5 | 8568,5 | 23430,7 |
| P12273 | Prolactin-inducible protein | PIP | PIP | 9553,2 | 10918,5 | 4802,0 | 9128,3 | 6813,4 | 31228,3 | 2385,5 | 3604,9 | 49379,9 |
| Q92820 | Gamma-glutamyl hydrolase | GGH | GGH | 8877,6 | 9119,3 | 11538,5 | 21315,7 | 12426,6 | 20357,0 | 2476,0 | 6803,5 | 51049,6 |
| A0A075B6K4 | Immunoglobulin lambda variable 3-10 | LV310 | IGLV3-10 | 97,0 | 140,5 | 121,3 | 55,3 | 110,2 | 213,5 | 83,0 | 246,2 | 1109,8 |
| Q13103 | Secreted phosphoprotein 24 | SPP24 | SPP2 | 9663,2 | 9093,7 | 4197,7 | 1739,7 | 4476,7 | 2758,3 | 3365,7 | 19010,9 | 6691,6 |
| P0DOX2 | Immunoglobulin alpha-2 heavy chain | IGA2 | 1 SV | 3398,7 | 251,4 | 177,9 | 19030,5 | 1559,1 | 1812,9 | 338,2 | 311,6 | 906,7 |
| Q16853 | Membrane primary amine oxidase | AOC3 | AOC3 | 4646,6 | 12280,1 | 1785,0 | 2399,1 | 60052,2 | 4677,6 | 5451,1 | 11272,7 | 36728,3 |
| Q8NI99 | Angiopoietin-related protein 6 | ANGL6 | ANGPTL6 | 391,4 | 552,9 | 199,7 | 1725,6 | 353,6 | 2320,9 | 101,6 | 267,8 | 1406,3 |
| Q86U17 | Serpin A11 | SPA11 | SERPINA11 | 2683,2 | 666,7 | 1233,1 | 2292,7 | 793,8 | 1297,1 | 2000,2 | 2345,7 | 1388,1 |
| P07333 | Macrophage colony-stimulating factor 1 receptor | CSF1R | CSF1R | 4127,5 | 5329,1 | 1699,3 | 971,4 | 11163,8 | 14163,2 | 1147,9 | 4653,0 | 8048,5 |
| P07737 | Profilin-1 | PROF1 | PFN1 | 55000,8 | 7345,4 | 21677,7 | 20427,7 | 36432,1 | 63564,5 | 3181,0 | 14940,5 | 27765,1 |
| P05362 | Intercellular adhesion molecule 1 | ICAM1 | ICAM1 | 2023,8 | 3899,4 | 396,1 | 638,9 | 387,1 | 526,3 | 3622,9 | 734,1 | 1957,2 |
| P49913 | Cathelicidin antimicrobial peptide | CAMP | CAMP | 8724,1 | 52435,8 | 24992,4 | 13961,6 | 32402,1 | 9701,0 | 468,2 | 38674,7 | 14599,0 |
| P04406 | Glyceraldehyde-3-phosphate dehydrogenase | G3P | GAPDH | 7745,2 | 10473,3 | 3699,7 | 4997,8 | 2773,4 | 3216,7 | 9259,5 | 1749,5 | 19710,3 |
| Q13740 | CD166 antigen | CD166 | ALCAM | 5051,4 | 3276,5 | 1575,9 | 1212,2 | 5505,9 | 23085,0 | 2033,0 | 10237,3 | 12823,0 |
| P03950 | Angiogenin | ANGI | ANG | 229,2 | 385,9 | 708,4 | 90,6 | 499,4 | 934,0 | 211,8 | 248,9 | 720,2 |
| P55056 | Apolipoprotein C-IV | APOC4 | APOC4 | 14953,0 | 1352,6 | 13218,1 | 9331,5 | 20914,6 | 6892,2 | 6726,8 | 22798,5 | 11742,4 |
| Q9NPH3 | Interleukin-1 receptor accessory protein | IL1AP | IL1RAP | 918,5 | 7127,2 | 4195,3 | 13369,8 | 18654,8 | 4142,0 | 11783,8 | 18423,0 | 8888,0 |
| P36980 | Complement factor H-related protein 2 | FHR2 | CFHR2 | 4549,2 | 2343,3 | 1506,6 | 6609,3 | 6603,8 | 2743,2 | 751,9 | 1241,3 | 4259,0 |
| P01860 | Immunoglobulin heavy constant gamma 3 | IGHG3 | IGHG3 | 360092,7 | 320742,6 | 125109,2 | 558773,0 | 140465,6 | 890443,3 | 588530,3 | 45459,4 | 15248,2 |
| P04083 | Annexin A1 | ANXA1 | ANXA1 | 238,7 | 692,0 | 1799,6 | 722,0 | 489,0 | 11929,9 | 6598,1 | 18374,0 | 12219,1 |
| A0A0C4DH38 | Immunoglobulin heavy variable 5-51 | HV551 | IGHV5-51 | 322,8 | 319,8 | 174,8 | 562,4 | 434,8 | 638,6 | 334,6 | 411,4 | 1095,8 |
| P12035 | Keratin, type II cytoskeletal 3 | K2C3 | KRT3 | 4816,3 | 3635,0 | 4176,7 | 2606,8 | 3082,4 | 2883,4 | 2326,0 | 17858,8 | 17653,5 |
| Q15848 | Adiponectin | ADIPO | ADIPOQ | 17714,5 | 1340,5 | 41342,5 | 11102,4 | 5076,8 | 22911,6 | 3197,2 | 5015,8 | 11014,0 |
| Q9H8L6 | Multimerin-2 | MMRN2 | MMRN2 | 885,0 | 4114,4 | 2134,7 | 3893,7 | 1310,5 | 6362,7 | 239,9 | 2473,0 | 14894,0 |
| P11717 | Cation-independent mannose-6-phosphate receptor | MPRI | IGF2R | 580498,3 | 1235004,5 | 964226,8 | 1015741,4 | 130832,2 | 2570014,6 | 722462,3 | 291808,1 | 74425,1 |
| P11597 | Cholesteryl ester transfer protein | CETP | CETP | 9734,8 | 18208,9 | 7981,5 | 74447,0 | 101132,2 | 50304,6 | 30623,6 | 87254,5 | 19252,8 |
| Q01469 | Fatty acid-binding protein, epidermal | FABP5 | FABP5 | 11814,7 | 7707,5 | 7671,4 | 1288,4 | 8353,3 | 9522,3 | 3238,5 | 7250,5 | 7005,6 |
| P05451 | Lithostathine-1-alpha | REG1A | REG1A | 7797,6 | 25042,1 | 19226,0 | 41801,5 | 56069,2 | 32801,3 | 9956,2 | 26502,1 | 53092,5 |
| Q9Y5Y7 | Lymphatic vessel endothelial hyaluronic acid receptor 1 | LYVE1 | LYVE1 | 3705,5 | 2597,1 | 4722,4 | 1955,8 | 2803,2 | 3791,6 | 1588,9 | 3587,8 | 3657,5 |
| P05109 | Protein S100-A8 | S10A8 | S100A8 | 75,9 | 112,2 | 121,7 | 222,0 | 155,4 | 380,9 | 77,5 | 124,4 | 972,1 |
| P07339 | Cathepsin D | CATD | CTSD | 2728,0 | 5140,8 | 7234,5 | 6124,8 | 16545,6 | 7652,8 | 4079,2 | 14708,2 | 16274,3 |
| P02775 | Platelet basic protein | CXCL7 | PPBP | 7357,6 | 18593,8 | 5413,6 | 9849,1 | 371,8 | 8863,7 | 2782,6 | 2373,5 | 1752,1 |
| P04433 | Immunoglobulin kappa variable 3-11 | KV311 | IGKV3-11 | 88,5 | 101,2 | 94,2 | 69,3 | 281,2 | 163,3 | 57,3 | 65,5 | 440,8 |
| P06331 | Immunoglobulin heavy variable 4-34 | HV434 | IGHV4-34 | 205,5 | 113,3 | 93,6 | 496,3 | 214,3 | 246,5 | 301,0 | 245,6 | 712,5 |
| P22897 | Macrophage mannose receptor 1 | MRC1 | MRC1 | 641,9 | 488,6 | 298,9 | 1503,0 | 1612,5 | 417,5 | 359,5 | 151,5 | 1691,0 |
| Q3SY84 | Keratin, type II cytoskeletal 71 | K2C71 | KRT71 | 3034,4 | 1212,4 | 2055,7 | 1534,9 | 5079,2 | 3146,3 | 932,3 | 2069,6 | 14028,5 |
| P00915 | Carbonic anhydrase 1 | CAH1 | CA1 | 95055,2 | 27515,8 | 24816,2 | 768,6 | 2119,2 | 916,2 | 14319,2 | 5328,5 | 8612,0 |
| P22735 | Protein-glutamine gamma-glutamyltransferase K | TGM1 | TGM1 | 163,9 | 178,0 | 357,9 | 474,1 | 1174,5 | 396,0 | 527,2 | 700,8 | 2984,1 |
| P61769 | Beta-2-microglobulin | B2MG | B2M | 18954,1 | 18464,3 | 13283,6 | 18965,8 | 3232,6 | 12536,1 | 29394,9 | 5809,6 | 22664,1 |
| P05089 | Arginase-1 | ARGI1 | ARG1 | 2426,1 | 5126,1 | 2525,2 | 3073,3 | 7551,7 | 7740,8 | 2684,9 | 18547,4 | 7293,2 |
| P01861 | Immunoglobulin heavy constant gamma 4 | IGHG4 | IGHG4 | 76,0 | 157,4 | 104,3 | 82,0 | 136,6 | 247,6 | 114,7 | 275,2 | 806,2 |
| Q13835 | Plakophilin-1 | PKP1 | PKP1 | 152,7 | 108,7 | 65,3 | 178,0 | 152,5 | 163,1 | 114,7 | 186,6 | 488,0 |
| P80748 | Immunoglobulin lambda variable 3-21 | LV321 | IGLV3-21 | 39,6 | 94,8 | 62,8 | 216,6 | 139,0 | 164,8 | 126,7 | 125,2 | 975,4 |
| P21333 | Filamin-A | FLNA | FLNA | 799,5 | 1418,6 | 1370,5 | 2263,2 | 3075,2 | 6156,6 | 1638,7 | 3858,7 | 7816,5 |
| P01700 | Immunoglobulin lambda variable 1-47 | LV147 | IGLV1-47 | 70,0 | 172,3 | 108,8 | 150,7 | 253,3 | 163,0 | 116,4 | 121,1 | 1228,7 |
| P13727 | Bone marrow proteoglycan | PRG2 | PRG2 | 2648,8 | 1000,0 | 495,9 | 2118,6 | 4728,9 | 2035,3 | 677,3 | 1192,5 | 8522,4 |
| P08195 | 4F2 cell-surface antigen heavy chain | 4F2 | SLC3A2 | 1217,2 | 743,0 | 739,0 | 4538,0 | 8641,7 | 2800,7 | 2054,8 | 2325,0 | 4131,2 |
| P01714 | Immunoglobulin lambda variable 3-19 | LV319 | IGLV3-19 | 152,0 | 142,9 | 219,8 | 181,1 | 261,4 | 350,3 | 171,5 | 187,3 | 976,5 |
| P25774 | Cathepsin S | CATS | CTSS | 2339,6 | 2616,6 | 1978,0 | 1894,0 | 9634,6 | 10520,1 | 4687,0 | 1263,2 | 3056,0 |
| O75223 | Gamma-glutamylcyclotransferase | GGCT | GGCT | 18043,8 | 24687,8 | 16613,5 | 35011,9 | 26720,2 | 28556,4 | 26882,7 | 11612,1 | 25291,6 |
| P30041 | Peroxiredoxin-6 | PRDX6 | PRDX6 | 1648,3 | 2042,0 | 5604,4 | 8618,8 | 12957,1 | 1411,8 | 782,8 | 15326,9 | 22887,0 |
| P62937 | Peptidyl-prolyl cis-trans isomerase A | PPIA | PPIA | 271,2 | 318,8 | 1362,9 | 451,0 | 2928,4 | 487,1 | 384,4 | 1154,2 | 1223,4 |
| P02008 | Hemoglobin subunit zeta | HBAZ | HBZ | 68,7 | 117,4 | 95,0 | 164,7 | 380,5 | 271,1 | 225,5 | 371,3 | 1437,2 |
| Q8N6C8 | Leukocyte immunoglobulin-like receptor subfamily A member 3 | LIRA3 | LILRA3 | 1600,0 | 2883,2 | 1914,4 | 17910,8 | 9379,7 | 243,3 | 259,2 | 614,0 | 6770,1 |
| P23528 | Cofilin-1 | COF1 | CFL1 | 164,8 | 133,3 | 158,8 | 99,9 | 261,1 | 161,7 | 243,7 | 290,4 | 1519,8 |
| Q6P179 | Endoplasmic reticulum aminopeptidase 2 | ERAP2 | ERAP2 | 4664,2 | 8009,8 | 1945,4 | 7916,7 | 7899,3 | 6226,2 | 1671,6 | 17557,8 | 13486,9 |
| P01624 | Immunoglobulin kappa variable 3-15 | KV315 | IGKV3-15 | 88,4 | 118,9 | 90,6 | 129,0 | 156,9 | 163,4 | 101,1 | 248,7 | 917,6 |
| P12830 | Cadherin-1 | CADH1 | CDH1 | 3259,6 | 2031,3 | 5727,1 | 9413,1 | 1647,5 | 1127,9 | 1134,3 | 3579,3 | 18161,6 |
| P0DJI8 | Serum amyloid A-1 protein | SAA1 | SAA1 | 148,4 | 113,3 | 189,3 | 78,1 | 590,3 | 191,6 | 200,8 | 172,8 | 725,5 |
| Q9UNN8 | Endothelial protein C receptor | EPCR | PROCR | 5285,3 | 7761,4 | 3241,3 | 9263,5 | 10725,7 | 29049,1 | 1092,7 | 26289,0 | 80301,0 |
| P14543 | Nidogen-1 | NID1 | NID1 | 1199,2 | 4589,1 | 1879,9 | 11477,4 | 11656,4 | 13040,8 | 4545,1 | 5160,2 | 12458,1 |
| P06702 | Protein S100-A9 | S10A9 | S100A9 | 30094,2 | 77126,1 | 1949,3 | 162193,2 | 232751,9 | 93014,1 | 55241,2 | 15168,5 | 74025,7 |
| P29508 | Serpin B3 | SPB3 | SERPINB3 | 1010,1 | 3939,9 | 2081,5 | 553,4 | 13239,5 | 4777,6 | 614,2 | 12737,0 | 20733,9 |
| P02654 | Apolipoprotein C-I | APOC1 | APOC1 | 14278,3 | 7280,4 | 7766,1 | 6788,1 | 5585,7 | 15724,9 | 2730,9 | 5604,5 | 13843,8 |
| P20933 | N(4)-(beta-N-acetylglucosaminyl)-L-asparaginase | ASPG | AGA | 3093,2 | 49852,1 | 531,4 | 180399,4 | 7739,7 | 1886,8 | 2634,2 | 15736,7 | 16721,0 |
| P01703 | Immunoglobulin lambda variable 1-40 | LV140 | IGLV1-40 | 59,5 | 102,7 | 114,7 | 202,0 | 138,4 | 217,6 | 86,0 | 256,8 | 732,0 |
| P16070 | CD44 antigen | CD44 | CD44 | 5513,2 | 5174,0 | 5147,5 | 12064,2 | 11171,6 | 5066,6 | 3826,3 | 4135,1 | 9280,0 |
| P13473 | Lysosome-associated membrane glycoprotein 2 | LAMP2 | LAMP2 | 4331,2 | 4536,9 | 5142,0 | 501,5 | 2570,7 | 2394,0 | 4700,8 | 7068,9 | 5522,7 |
| P20930 | Filaggrin | FILA | FLG | 66,6 | 166,0 | 217,4 | 140,3 | 209,3 | 161,8 | 74,8 | 248,8 | 695,3 |
| P23284 | Peptidyl-prolyl cis-trans isomerase B | PPIB | PPIB | 554,3 | 292,4 | 1401,3 | 2006,0 | 1401,0 | 31598,1 | 5416,8 | 1325,9 | 18639,4 |
| P01611 | Immunoglobulin kappa variable 1D-12 | KVD12 | IGKV1D-12 | 91,3 | 113,3 | 93,3 | 96,3 | 271,4 | 134,2 | 108,4 | 168,9 | 1335,9 |
| P13598 | Intercellular adhesion molecule 2 | ICAM2 | ICAM2 | 1362,8 | 2067,8 | 1945,0 | 1613,4 | 1105,3 | 770,0 | 1634,8 | 1019,2 | 6991,2 |
| P01344 | Insulin-like growth factor II | IGF2 | IGF2 | 14572,7 | 17313,6 | 10846,9 | 14315,4 | 4931,4 | 12324,9 | 1448,5 | 9754,1 | 15024,5 |
| P20023 | Complement receptor type 2 | CR2 | CR2 | 448,2 | 1643,2 | 403,1 | 743,3 | 8958,2 | 3902,5 | 1021,8 | 2840,9 | 9960,0 |
| Q13790 | Apolipoprotein F | APOF | APOF | 10326,3 | 37847,6 | 21587,3 | 16529,8 | 25721,5 | 16519,4 | 9357,5 | 6966,6 | 37199,3 |
| P63267 | Actin, gamma-enteric smooth muscle | ACTH | ACTG2 | 3625,6 | 1454,9 | 4044,5 | 3085,6 | 2571,9 | 26200,1 | 8487,6 | 5306,2 | 9322,1 |
| P01743 | Immunoglobulin heavy variable 1-46 | HV146 | IGHV1-46 | 48816,8 | 45061,8 | 29980,2 | 171241,2 | 245,3 | 55790,4 | 113,9 | 307,2 | 487,9 |
| P52566 | Rho GDP-dissociation inhibitor 2 | GDIR2 | ARHGDIB | 1336,1 | 3501,2 | 2929,5 | 2547,5 | 2292,8 | 1431,9 | 4067,6 | 1911,8 | 10048,3 |
| Q8TER0 | Sushi, nidogen and EGF-like domain-containing protein 1 | SNED1 | SNED1 | 1023,0 | 347,9 | 5924,7 | 5561,5 | 17162,9 | 24373,1 | 5028,7 | 9627,9 | 17861,0 |
| P15814 | Immunoglobulin lambda-like polypeptide 1 | IGLL1 | IGLL1 | 3948,8 | 2825,8 | 3236,8 | 6113,4 | 416,5 | 5194,6 | 4833,6 | 181,9 | 901,2 |
| Q6YHK3 | CD109 antigen | CD109 | CD109 | 3103,4 | 6667,4 | 4132,1 | 1938,9 | 8660,7 | 2836,3 | 2597,3 | 2907,6 | 17155,0 |
| Q13867 | Bleomycin hydrolase | BLMH | BLMH | 1590,2 | 2023,5 | 4276,4 | 11977,1 | 15361,0 | 5411,8 | 1270,9 | 1169,9 | 14351,6 |
| Q86SQ4 | Adhesion G-protein coupled receptor G6 | AGRG6 | ADGRG6 | 668,9 | 1356,0 | 1020,3 | 891,9 | 2404,8 | 8281,0 | 527,8 | 5495,2 | 14414,2 |
| P62979 | Ubiquitin-40S ribosomal protein S27a | RS27A | RPS27A | 68,2 | 134,5 | 91,0 | 96,3 | 173,1 | 152,9 | 86,0 | 124,4 | 731,9 |
| P41222 | Prostaglandin-H2 D-isomerase | PTGDS | PTGDS | 3190,9 | 5356,4 | 4306,5 | 25103,7 | 8896,9 | 22219,9 | 3036,5 | 28530,8 | 9765,7 |
| P04075 | Fructose-bisphosphate aldolase A | ALDOA | ALDOA | 2294,6 | 17545,7 | 21729,6 | 7302,5 | 30822,0 | 42063,4 | 3350,8 | 13244,5 | 48293,7 |
| Q96QA5 | Gasdermin-A | GSDMA | GSDMA | 2136,2 | 2905,4 | 1300,3 | 9071,8 | 19839,5 | 7038,4 | 2982,0 | 10403,7 | 18295,4 |
| P09211 | Glutathione S-transferase P | GSTP1 | GSTP1 | 75,9 | 116,6 | 39,5 | 204,1 | 209,7 | 224,3 | 172,9 | 122,7 | 818,2 |
| Q9NZ08 | Endoplasmic reticulum aminopeptidase 1 | ERAP1 | ERAP1 | 1337,3 | 1492,7 | 4789,2 | 1098,4 | 2296,5 | 1381,7 | 3452,3 | 3562,0 | 10173,0 |
| A0A0B4J1X5 | Immunoglobulin heavy variable 3-74 | HV374 | IGHV3-74 | 126,6 | 175,5 | 156,7 | 76,3 | 268,0 | 347,6 | 81,7 | 186,7 | 1107,7 |
| P24593 | Insulin-like growth factor-binding protein 5 | IBP5 | IGFBP5 | 479,0 | 578,6 | 151,6 | 2939,0 | 1070,7 | 1034,5 | 1105,5 | 306,2 | 2480,4 |
| P49721 | Proteasome subunit beta type-2 | PSB2 | PSMB2 | 1472,4 | 4454,2 | 2184,4 | 11521,6 | 19177,4 | 8127,7 | 3104,5 | 2274,0 | 9774,8 |
| Q9P2D3 | HEAT repeat-containing protein 5B | HTR5B | HEATR5B | 4606,2 | 1862,3 | 395,2 | 316,4 | 158,8 | 116,8 | 411,2 | 3292,8 | 2384,9 |
| P0DP09 | Immunoglobulin kappa variable 1-13 | KV113 | IGKV1-13 | 269,5 | 216,5 | 138,4 | 629,9 | 482,4 | 338,2 | 251,2 | 436,3 | 1704,5 |
| Q96PC5 | Melanoma inhibitory activity protein 2 | MIA2 | MIA2 | 1293,3 | 563,5 | 1117,3 | 2570,4 | 1771,0 | 930,4 | 385,9 | 1018,7 | 6227,7 |
| Q9P225 | Dynein heavy chain 2, axonemal | DYH2 | DNAH2 | 1107,1 | 1142,3 | 886,0 | 2297,7 | 2319,3 | 2024,1 | 1086,7 | 1546,7 | 5034,7 |
| Q9Y3R5 | Protein dopey-2 | DOP2 | DOPEY2 | 1335,9 | 1017,5 | 466,0 | 1223,2 | 2445,4 | 562,6 | 419,1 | 1608,3 | 9474,3 |

**Supplemental Table 6. Proteomic profile of plasma before (PRE), during (APHERESIS) and after (POST) stem cell mobilization and infusion in women with premature ovarian insufficiency (POI).**

| Protein ID | Protein name | Protein name abbreviation | Gene name | P1_ASCOT-Pre | P4_ASCOT-Pre | P8_ASCOT-Pre | P1-ASCOT-Apheresis | P4_ASCOT-Apheresis | P8_ASCOT-Apheresis | P1_ASCOT-Post | P4_ASCOT-Post | P8_ASCOT-Post | P3_MOB-Pre | P5_MOB-Pre | P6_MOB-Pre | P3_MOB-Apheresis | P5_MOB-Apheresis | P6_MOB-Apheresis | P3_MOB-Post | P5_MOB-Post | P6_MOB-Post |
| --- | --- | --- | --- | --- | --- | --- | --- | --- | --- | --- | --- | --- | --- | --- | --- | --- | --- | --- | --- | --- | --- |
| P04114 | Apolipoprotein B-100 | APOB | APOB | 10113611,3 | 11658046,4 | 5804021,0 | 5649975,3 | 9603767,5 | 4614403,7 | 9084339,0 | 10974486,6 | 5186376,3 | 6970010,8 | 9335206,9 | 6819910,9 | 5283715,9 | 4318660,4 | 7648354,9 | 6412005,0 | 7771814,3 | 5859974,6 |
| P0C0L5 | Complement C4-B | CO4B | C4B | 290300,1 | 175944,1 | 146457,0 | 286239,1 | 212528,6 | 90751,8 | 119071,9 | 141152,9 | 148276,2 | 79028,1 | 155340,0 | 153532,8 | 235421,3 | 163883,4 | 270425,5 | 174364,9 | 117752,2 | 38775,0 |
| P02751 | Fibronectin | FINC | FN1 | 1930265,2 | 2299487,8 | 5947681,3 | 5563795,8 | 6356452,3 | 3560519,7 | 6739968,0 | 4069036,5 | 5403425,5 | 4751287,5 | 6120635,0 | 5191828,8 | 5042024,2 | 5437390,5 | 3080227,5 | 5863260,7 | 5102814,9 | 5217097,0 |
| P00450 | Ceruloplasmin | CERU | CP | 1121141,5 | 1568347,8 | 803191,1 | 1566485,5 | 1398712,0 | 963629,2 | 570111,9 | 801100,3 | 1094018,4 | 774654,7 | 744808,1 | 531345,3 | 551692,0 | 761350,3 | 747508,0 | 1237310,2 | 847345,4 | 988345,0 |
| P01023 | Alpha-2-macroglobulin | A2MG | A2M | 397186,5 | 407223,3 | 433255,5 | 275338,7 | 380846,3 | 277482,7 | 417536,2 | 415789,6 | 420093,0 | 326828,7 | 266789,4 | 238691,2 | 405430,6 | 254443,5 | 566933,7 | 248356,1 | 290938,1 | 203346,2 |
| P01031 | Complement C5 | CO5 | C5 | 1973894,2 | 1948444,7 | 1951060,8 | 2126334,2 | 2137680,9 | 2220973,9 | 1706697,0 | 1775217,1 | 1819317,7 | 1445676,6 | 1605028,8 | 1172638,8 | 2017952,3 | 1793505,2 | 2270928,1 | 1513648,6 | 913269,0 | 1493900,4 |
| P08603 | Complement factor H | CFAH | CFH | 3909925,2 | 3089337,4 | 3040420,3 | 3822370,5 | 3927112,7 | 3219105,7 | 3032557,9 | 2660771,2 | 2854197,3 | 2731512,6 | 4343989,7 | 2306492,0 | 2843394,9 | 4743601,1 | 3689198,9 | 3086284,8 | 2849190,1 | 2233431,7 |
| P02768 | Serum albumin | ALBU | ALB | 110676208,2 | 119713082,1 | 120695859,5 | 103304656,0 | 98592750,0 | 112469927,1 | 132491434,2 | 137792051,3 | 118859075,5 | 149736354,0 | 136701546,0 | 173126078,0 | 135195820,0 | 127146454,6 | 123290199,6 | 134732817,5 | 158882516,9 | 160504839,3 |
| P02790 | Hemopexin | HEMO | HPX | 24249130,7 | 19456564,7 | 29816374,3 | 23163499,0 | 24563468,5 | 26060721,8 | 17303341,2 | 21136621,9 | 30274625,4 | 19099484,3 | 25603592,5 | 12907843,2 | 18399222,0 | 21498899,3 | 28314032,2 | 19544832,4 | 17453517,9 | 14776627,5 |
| P00747 | Plasminogen | PLMN | PLG | 3336040,3 | 3568081,7 | 3088788,1 | 3047486,8 | 4679504,3 | 3574530,5 | 2849865,6 | 3129104,2 | 3321496,6 | 2746004,6 | 3974914,4 | 2515709,5 | 3061075,6 | 3328534,3 | 3904537,5 | 3867004,9 | 2180202,7 | 3265583,6 |
| Q14624 | Inter-alpha-trypsin inhibitor heavy chain H4 | ITIH4 | ITIH4 | 3239806,9 | 2757201,1 | 3542159,7 | 3811356,5 | 3899730,0 | 3679859,6 | 3020871,1 | 2940655,6 | 3195788,6 | 2616307,9 | 4003668,0 | 2633076,4 | 2811709,5 | 4243441,8 | 4345943,8 | 3477638,1 | 2496160,7 | 3364767,3 |
| P01024 | Complement C3 | CO3 | C3 | 76760,9 | 48917,0 | 67358,0 | 39919,5 | 49889,2 | 12609,6 | 20363,7 | 43244,1 | 12707,6 | 27750,9 | 13179,3 | 22176,5 | 33079,3 | 15047,5 | 38848,8 | 11488,6 | 66809,4 | 35786,2 |
| P00751 | Complement factor B | CFAB | CFB | 3294470,3 | 3099914,7 | 4528231,8 | 4376148,8 | 5196228,7 | 5317346,0 | 3408293,3 | 3114896,6 | 3951251,2 | 3540957,0 | 4840737,7 | 2660843,9 | 3865664,5 | 5518934,4 | 4005283,5 | 3420853,8 | 3257466,8 | 2489607,5 |
| P02787 | Serotransferrin | TRFE | TF | 21502,6 | 7131,2 | 26365,2 | 26786,2 | 21698,6 | 48991,0 | 15009,8 | 30924,8 | 21922,8 | 30030,0 | 14515,1 | 21336,9 | 15604,9 | 40002,4 | 12925,4 | 19054,4 | 26950,3 | 29587,6 |
| P02774 | Vitamin D-binding protein | VTDB | GC | 1404884,2 | 870590,2 | 761838,4 | 1596160,5 | 2267888,0 | 1050973,4 | 684056,1 | 1215780,2 | 1097260,3 | 1319235,7 | 2015568,3 | 865211,1 | 1316564,2 | 1437415,2 | 1096417,9 | 2739744,6 | 840192,2 | 1238990,9 |
| P01011 | Alpha-1-antichymotrypsin | AACT | SERPINA3 | 7948357,7 | 11898811,3 | 8073885,2 | 10326075,2 | 17752736,6 | 16265301,4 | 8887677,0 | 6317890,9 | 14868471,5 | 11115799,0 | 11383921,9 | 4622292,1 | 11687298,0 | 14463578,0 | 9364212,5 | 11788986,8 | 3711719,4 | 4826001,2 |
| P19823 | Inter-alpha-trypsin inhibitor heavy chain H2 | ITIH2 | ITIH2 | 3223968,0 | 2869926,8 | 4381863,7 | 2889112,3 | 3022891,3 | 2992492,4 | 3380557,7 | 2752704,7 | 2626865,8 | 2375548,0 | 3974857,5 | 1951955,2 | 2497283,2 | 3577354,4 | 4078159,7 | 2807153,3 | 2339668,8 | 2973381,8 |
| P00734 | Prothrombin | THRB | F2 | 5027177,9 | 4029335,4 | 4028755,8 | 4597191,4 | 6472159,7 | 3534148,7 | 3318320,4 | 4806763,3 | 3494954,0 | 3986130,8 | 5316524,2 | 2504080,2 | 2844091,1 | 4901219,0 | 5372853,7 | 4911049,1 | 3506199,4 | 3218293,6 |
| P19827 | Inter-alpha-trypsin inhibitor heavy chain H1 | ITIH1 | ITIH1 | 3844074,1 | 3359277,5 | 3949934,3 | 4406943,7 | 3752764,3 | 3811470,6 | 3640059,2 | 3054834,1 | 3838003,5 | 3195181,2 | 3516526,6 | 2631107,0 | 3538334,4 | 4881949,1 | 3660389,3 | 4004228,3 | 3133012,8 | 2823440,6 |
| P02671 | Fibrinogen alpha chain | FIBA | FGA | 26568,0 | 18028,4 | 35780,8 | 15670,7 | 20762,3 | 18086,8 | 46329,6 | 32931,6 | 25018,5 | 11868,3 | 12574,4 | 21429,0 | 22753,9 | 12113,7 | 28825,6 | 27607,6 | 25481,3 | 38282,9 |
| P06396 | Gelsolin | GELS | GSN | 1178043,2 | 1198649,1 | 1135731,2 | 1035456,2 | 1207128,3 | 933313,2 | 733794,3 | 797184,2 | 945721,3 | 844344,0 | 973579,7 | 668926,8 | 575272,2 | 775578,9 | 947211,3 | 820903,8 | 975534,8 | 617195,6 |
| P01008 | Antithrombin-III | ANT3 | SERPINC1 | 1876912,4 | 1657273,1 | 1888569,6 | 1742574,6 | 1644918,8 | 1678538,2 | 1620693,9 | 1347688,3 | 1861551,8 | 1436684,4 | 2134302,9 | 1450273,1 | 1217353,6 | 1827407,6 | 1781550,6 | 1154405,1 | 1368355,4 | 1094927,2 |
| P01042 | Kininogen-1 | KNG1 | KNG1 | 3534790,8 | 3148060,0 | 2492506,5 | 3589684,8 | 3144890,0 | 3784516,1 | 2142909,2 | 2401562,2 | 3488508,7 | 2946825,4 | 3296292,3 | 2187133,4 | 2793657,7 | 3024778,9 | 3797793,7 | 3735441,4 | 3841253,2 | 2860159,3 |
| P06727 | Apolipoprotein A-IV | APOA4 | APOA4 | 6009528,3 | 4463928,7 | 4657667,1 | 3488476,5 | 3826853,8 | 5212099,6 | 4350785,0 | 4303166,7 | 3156397,9 | 3465018,2 | 5645165,5 | 6049888,0 | 4481998,8 | 3838219,8 | 6118534,7 | 5118578,4 | 6258982,0 | 5086167,0 |
| P43652 | Afamin | AFAM | AFM | 746523,6 | 198163,9 | 368179,8 | 500523,5 | 232963,1 | 517400,5 | 521495,7 | 356212,9 | 216812,6 | 339286,0 | 168748,2 | 305732,4 | 174485,7 | 176353,8 | 389885,8 | 217708,9 | 443815,6 | 364111,9 |
| P13671 | Complement component C6 | CO6 | C6 | 394428,1 | 276482,2 | 279277,6 | 439061,8 | 394119,2 | 321293,0 | 320689,2 | 309899,5 | 232309,7 | 318535,3 | 275686,1 | 314527,9 | 341184,4 | 338083,6 | 358540,8 | 297528,1 | 289910,7 | 322610,8 |
| P12259 | Coagulation factor V | FA5 | F5 | 218969,0 | 244932,2 | 243693,3 | 376196,8 | 323499,3 | 260117,2 | 203791,8 | 245169,1 | 274802,0 | 202475,7 | 226292,0 | 335061,2 | 262211,5 | 291489,1 | 535623,9 | 212832,9 | 343944,5 | 288553,7 |
| P04217 | Alpha-1B-glycoprotein | A1BG | A1BG | 4494747,3 | 4696373,3 | 3174371,3 | 5822177,6 | 6100821,3 | 4170161,4 | 2653620,9 | 3519332,4 | 4433983,8 | 4080071,7 | 4567731,1 | 2541294,2 | 3850715,2 | 4763485,9 | 2954201,2 | 3823319,3 | 2678396,0 | 2284277,4 |
| P01009 | Alpha-1-antitrypsin | A1AT | SERPINA1 | 11069,1 | 17358,4 | 11718,1 | 5638,8 | 20348,2 | 5030,5 | 10329,1 | 9131,4 | 20706,6 | 6142,2 | 10190,4 | 27663,0 | 13765,0 | 4917,5 | 23863,8 | 8366,4 | 23197,6 | 13773,2 |
| P01019 | Angiotensinogen | ANGT | AGT | 953425,6 | 1741841,9 | 1317492,0 | 1770200,1 | 755424,4 | 1849576,7 | 615477,5 | 515172,4 | 1430136,9 | 1049944,0 | 1782122,6 | 932921,7 | 1046470,5 | 1694442,8 | 1629935,6 | 1897021,9 | 1070938,7 | 1199607,9 |
| P04264 | Keratin, type II cytoskeletal 1 | K2C1 | KRT1 | 4403115,7 | 3371704,7 | 1907793,3 | 3305814,2 | 1812770,4 | 368582,2 | 2568746,1 | 1876806,8 | 1872616,2 | 1963760,0 | 758638,8 | 1184434,8 | 1722203,2 | 664741,4 | 766694,7 | 918550,2 | 1834633,9 | 755384,8 |
| P09871 | Complement C1s subcomponent | C1S | C1S | 489536,9 | 604519,8 | 425400,4 | 491240,0 | 677753,8 | 393560,9 | 347862,6 | 400702,2 | 294899,2 | 474289,4 | 382063,7 | 332680,4 | 519898,4 | 524634,2 | 630693,6 | 402761,8 | 377734,9 | 398524,9 |
| P05155 | Plasma protease C1 inhibitor | IC1 | SERPING1 | 2343271,6 | 1721086,8 | 1473950,3 | 2089678,1 | 2371780,8 | 1367526,9 | 1431935,0 | 1378725,0 | 2449474,4 | 1033285,3 | 2647192,1 | 1102891,4 | 1728692,3 | 2041398,1 | 1279462,9 | 1302051,5 | 550076,6 | 750011,0 |
| P02749 | Beta-2-glycoprotein 1 | APOH | APOH | 1273891,9 | 1331624,2 | 1315247,1 | 889456,5 | 902747,2 | 1340728,2 | 1263151,7 | 1052911,4 | 1480353,6 | 1497004,6 | 734510,5 | 1384334,0 | 928781,7 | 583788,5 | 1228452,7 | 1311885,2 | 1369402,2 | 1347393,0 |
| P10643 | Complement component C7 | CO7 | C7 | 1205554,4 | 1102070,6 | 744036,7 | 1348507,7 | 1448943,5 | 967727,9 | 385451,9 | 1164927,8 | 679401,7 | 971923,0 | 386769,5 | 815312,8 | 939370,2 | 467732,1 | 1013122,7 | 1025819,8 | 943821,7 | 814008,8 |
| P04275 | von Willebrand factor | VWF | VWF | 106873,7 | 89002,2 | 232611,7 | 461444,8 | 294413,0 | 157347,2 | 212378,2 | 40071,0 | 178158,3 | 103945,5 | 138232,1 | 97124,9 | 192341,2 | 256409,6 | 37287,2 | 81256,4 | 165096,4 | 171703,1 |
| P00736 | Complement C1r subcomponent | C1R | C1R | 835709,7 | 784047,4 | 705623,6 | 1081025,3 | 1224160,9 | 933494,3 | 645901,4 | 791392,2 | 687570,8 | 830481,1 | 747585,5 | 649468,4 | 942833,8 | 1026587,8 | 1173372,7 | 940861,9 | 667342,1 | 648610,3 |
| P02675 | Fibrinogen beta chain | FIBB | FGB | 16248,0 | 8495,1 | 10829,8 | 25962,4 | 18154,1 | 13511,9 | 29041,9 | 16278,3 | 15247,8 | 15196,9 | 9321,2 | 20151,6 | 11099,2 | 16763,1 | 12806,3 | 17131,8 | 12554,1 | 22918,4 |
| P02765 | Alpha-2-HS-glycoprotein | FETUA | AHSG | 442879,6 | 522380,2 | 421655,6 | 588257,0 | 441881,5 | 487193,4 | 332288,9 | 328232,6 | 446814,6 | 391195,8 | 289985,2 | 475211,2 | 311955,8 | 292489,9 | 431483,4 | 269346,8 | 333136,9 | 406870,7 |
| P08697 | Alpha-2-antiplasmin | A2AP | SERPINF2 | 1124843,1 | 1044505,2 | 1420799,8 | 1236223,7 | 1210655,9 | 1226765,8 | 1077180,9 | 1088709,2 | 953071,3 | 1233353,1 | 1704106,6 | 1069894,6 | 999219,2 | 1141786,2 | 1473112,4 | 1131586,9 | 1273859,5 | 1190747,5 |
| P00738 | Haptoglobin | HPT | HP | 414685,4 | 495553,8 | 180365,7 | 421350,2 | 481448,7 | 553825,4 | 435315,6 | 396823,2 | 743710,8 | 118525,3 | 76037,1 | 145909,4 | 250787,0 | 296047,4 | 589617,4 | 74891,4 | 292141,8 | 358572,1 |
| P04003 | C4b-binding protein alpha chain | C4BPA | C4BPA | 2195741,9 | 1750295,5 | 2408885,0 | 2191212,9 | 2368765,5 | 3331439,4 | 2962412,3 | 1970953,1 | 1488535,5 | 2153279,1 | 3000186,0 | 1881261,2 | 2391690,5 | 4313614,3 | 1624223,2 | 1553214,8 | 658642,8 | 1419001,1 |
| P02748 | Complement component C9 | CO9 | C9 | 1984351,7 | 1875939,1 | 3182443,1 | 2674802,9 | 3421178,0 | 3530632,2 | 1937164,2 | 2206734,1 | 3118512,7 | 1351725,1 | 1964013,1 | 1682382,5 | 2401819,6 | 3371457,4 | 3547828,0 | 1596735,1 | 1746316,1 | 2083425,2 |
| P02647 | Apolipoprotein A-I | APOA1 | APOA1 | 11201,9 | 4104,6 | 4205,2 | 5466,4 | 6239,0 | 2934,5 | 10898,3 | 6646,0 | 5581,2 | 5537,5 | 6636,3 | 11820,2 | 10210,0 | 4723,2 | 7384,3 | 7536,2 | 7613,6 | 7515,3 |
| P35908 | Keratin, type II cytoskeletal 2 epidermal | K22E | KRT2 | 1739998,9 | 1356313,3 | 1267567,9 | 1255129,2 | 599258,9 | 569084,3 | 1882949,4 | 566028,2 | 1128651,0 | 730141,1 | 264326,3 | 516870,5 | 2149995,9 | 789306,6 | 361101,0 | 417085,5 | 527633,0 | 965011,6 |
| Q06033 | Inter-alpha-trypsin inhibitor heavy chain H3 | ITIH3 | ITIH3 | 403263,7 | 486901,2 | 540879,2 | 552872,5 | 634029,3 | 449791,7 | 292849,7 | 326527,6 | 513914,5 | 237436,3 | 302625,9 | 263548,9 | 459739,4 | 440291,5 | 655476,0 | 250407,7 | 156234,4 | 167593,8 |
| P35527 | Keratin, type I cytoskeletal 9 | K1C9 | KRT9 | 274547,0 | 279236,8 | 112922,0 | 401076,6 | 95673,6 | 194795,5 | 478057,8 | 86046,8 | 180706,9 | 46267,9 | 68558,7 | 130508,4 | 468759,1 | 45210,7 | 36141,1 | 83485,3 | 70482,9 | 60139,2 |
| P06681 | Complement C2 | CO2 | C2 | 257810,5 | 294842,6 | 317863,4 | 388875,9 | 404140,3 | 280694,8 | 362374,1 | 277384,3 | 260659,9 | 252819,2 | 247548,2 | 207828,2 | 304710,5 | 286929,8 | 384688,1 | 237917,9 | 297449,3 | 193559,1 |
| Q9Y6R7 | IgGFc-binding protein | FCGBP | FCGBP | 115535,6 | 75069,9 | 175124,2 | 136637,9 | 144085,8 | 86384,2 | 87787,0 | 77978,8 | 72640,1 | 62049,5 | 103069,0 | 124556,1 | 146074,7 | 116600,0 | 90443,7 | 136229,8 | 50461,6 | 98021,0 |
| P07358 | Complement component C8 beta chain | CO8B | C8B | 769343,9 | 664321,2 | 793838,6 | 617530,7 | 620581,9 | 702061,9 | 873394,9 | 728445,2 | 363224,1 | 433866,6 | 526332,7 | 716659,7 | 997778,3 | 496095,7 | 810481,3 | 707504,2 | 602125,4 | 596955,1 |
| P03952 | Plasma kallikrein | KLKB1 | KLKB1 | 1270359,7 | 743121,4 | 1261832,7 | 1412887,9 | 1272729,9 | 1105537,6 | 1042781,4 | 1107749,8 | 862183,9 | 1025848,4 | 390695,5 | 993633,7 | 961861,6 | 1278778,7 | 1288147,8 | 1155844,5 | 1282472,2 | 1260097,3 |
| P04004 | Vitronectin | VTNC | VTN | 1965816,0 | 1324424,1 | 2314568,1 | 1701016,6 | 2202228,3 | 2309343,6 | 1508461,0 | 1515933,8 | 2485991,1 | 1220282,9 | 2543461,8 | 888244,2 | 1759445,6 | 2076764,5 | 3148376,0 | 1311192,8 | 1974451,9 | 1122783,9 |
| P13645 | Keratin, type I cytoskeletal 10 | K1C10 | KRT10 | 4528186,9 | 4736207,2 | 3341755,0 | 6877856,8 | 1671342,5 | 2258848,0 | 8219648,7 | 2851545,1 | 2202485,8 | 2323830,6 | 1309113,3 | 3350865,1 | 6420604,4 | 1809246,9 | 1549548,6 | 1410535,1 | 1144468,1 | 4137013,6 |
| P05156 | Complement factor I | CFAI | CFI | 1078634,8 | 1025453,6 | 1012149,4 | 1397984,4 | 1377779,1 | 1686889,5 | 1080577,1 | 754217,7 | 1304884,2 | 1090573,2 | 1545581,1 | 1053405,8 | 997833,8 | 1746400,4 | 1003847,2 | 972504,7 | 899892,5 | 770541,5 |
| Q96PD5 | N-acetylmuramoyl-L-alanine amidase | PGRP2 | PGLYRP2 | 363488,0 | 686381,2 | 520419,7 | 569617,3 | 561265,2 | 562403,1 | 543113,0 | 650207,6 | 682223,4 | 689192,0 | 580337,6 | 367727,5 | 545594,1 | 742620,0 | 749308,8 | 709623,4 | 810547,0 | 473786,0 |
| O75882 | Attractin | ATRN | ATRN | 74981,2 | 36368,4 | 52777,0 | 63988,9 | 64467,2 | 50012,2 | 40408,5 | 43218,0 | 61862,5 | 76896,0 | 52991,5 | 87157,9 | 55904,7 | 46265,3 | 53859,4 | 49962,5 | 43929,7 | 49784,5 |
| P08519 | Apolipoprotein(a) | APOA | LPA | 2069113,4 | 350077,2 | 787095,9 | 1625886,0 | 290165,5 | 393457,0 | 1658378,1 | 330895,4 | 558802,0 | 245445,7 | 1373275,0 | 364006,3 | 268681,2 | 1890131,6 | 345978,1 | 357288,4 | 1919579,8 | 569445,7 |
| P05546 | Heparin cofactor 2 | HEP2 | SERPIND1 | 2032458,7 | 1966804,3 | 1229433,3 | 1974699,8 | 1984873,4 | 1869841,6 | 2267908,9 | 1363980,5 | 1639634,7 | 914904,1 | 1587929,5 | 1117367,7 | 1179803,1 | 1603152,0 | 1679506,1 | 1516399,0 | 705391,7 | 1391919,9 |
| P02679 | Fibrinogen gamma chain | FIBG | FGG | 6389,5 | 11204,5 | 8719,1 | 12616,4 | 40736,9 | 12364,3 | 12020,2 | 148217,7 | 10207,6 | 7041,1 | 7493,0 | 16708,3 | 14662,3 | 12544,9 | 20186,9 | 12343,4 | 16657,9 | 15346,3 |
| P00488 | Coagulation factor XIII A chain | F13A | F13A1 | 54507,1 | 51442,2 | 106825,9 | 104374,7 | 70544,4 | 78945,0 | 72287,7 | 92539,0 | 68899,4 | 77214,2 | 66004,1 | 41164,9 | 59998,4 | 56601,9 | 68740,6 | 58915,0 | 104768,2 | 35500,0 |
| P05160 | Coagulation factor XIII B chain | F13B | F13B | 303961,0 | 303409,2 | 330768,0 | 430901,9 | 254417,7 | 292485,0 | 273927,0 | 240634,9 | 269344,7 | 240772,2 | 122295,2 | 283682,6 | 215212,4 | 235811,5 | 269477,5 | 275451,6 | 314769,5 | 291654,7 |
| P20742 | Pregnancy zone protein | PZP | PZP | 8196341,1 | 7941204,6 | 5556714,9 | 8529290,4 | 8640173,7 | 5874139,3 | 182607,1 | 6231694,7 | 4635115,5 | 7935447,9 | 4531255,5 | 4732959,8 | 7469793,1 | 5216912,2 | 6420015,5 | 8979395,3 | 5986127,0 | 5245579,4 |
| P00748 | Coagulation factor XII | FA12 | F12 | 1535941,4 | 693391,3 | 737924,5 | 1463835,9 | 968393,4 | 838835,4 | 1107983,5 | 764990,1 | 794024,7 | 827368,4 | 1205964,5 | 782981,9 | 651576,2 | 1310305,6 | 970921,4 | 953448,0 | 1151717,0 | 1065587,0 |
| P04196 | Histidine-rich glycoprotein | HRG | HRG | 284173,1 | 1047320,1 | 184213,1 | 1435682,2 | 894925,7 | 290959,1 | 597423,7 | 403222,4 | 647366,0 | 361717,1 | 865514,5 | 248650,4 | 903314,4 | 684020,2 | 374555,2 | 835717,8 | 361150,3 | 246832,2 |
| P10909 | Clusterin | CLUS | CLU | 673366,7 | 636568,0 | 158905,5 | 504789,6 | 576310,6 | 426133,9 | 311289,1 | 393060,7 | 382303,6 | 164656,3 | 158039,7 | 151590,5 | 232232,0 | 216134,7 | 119133,1 | 633462,7 | 406019,4 | 127031,2 |
| P0DOX5 | Immunoglobulin gamma-1 heavy chain | IGG1 | 1 SV | 2221,5 | 2942,0 | 10930,5 | 5274,5 | 9423,2 | 4869,5 | 8337,6 | 14711,9 | 11528,1 | 2037,0 | 3829,8 | 2007,0 | 8449,8 | 10307,7 | 8734,5 | 3016,4 | 5812,0 | 6028,3 |
| P36955 | Pigment epithelium-derived factor | PEDF | SERPINF1 | 861135,8 | 703217,1 | 668307,1 | 933885,8 | 730063,5 | 753904,5 | 547467,2 | 610068,5 | 701452,2 | 530538,6 | 783738,6 | 576225,5 | 587843,0 | 689881,3 | 898724,5 | 529655,5 | 655869,9 | 609683,1 |
| P02760 | Protein AMBP | AMBP | AMBP | 2720031,5 | 1936000,5 | 3048740,6 | 2838782,2 | 2972326,6 | 3448356,2 | 2359106,9 | 2267397,5 | 2943002,1 | 2333309,1 | 2484413,8 | 1768967,7 | 2535696,7 | 3370369,6 | 3224184,4 | 2577932,3 | 2524650,5 | 2156039,5 |
| P26927 | Hepatocyte growth factor-like protein | HGFL | MST1 | 180732,3 | 154871,2 | 180388,0 | 191230,8 | 197600,6 | 157894,4 | 140934,2 | 172682,8 | 128504,5 | 132583,7 | 147405,7 | 167196,7 | 182887,6 | 194078,9 | 200103,3 | 152940,3 | 141697,8 | 204409,0 |
| P25311 | Zinc-alpha-2-glycoprotein | ZA2G | AZGP1 | 94568,1 | 87932,9 | 76830,4 | 71186,7 | 76545,7 | 75690,7 | 111969,6 | 93375,0 | 91790,7 | 70594,6 | 52534,4 | 120151,6 | 83560,3 | 51424,4 | 65317,8 | 90126,4 | 107230,9 | 98588,4 |
| P15924 | Desmoplakin | DESP | DSP | 80391,4 | 202951,1 | 46478,9 | 106703,5 | 141846,1 | 40053,8 | 112023,0 | 30884,4 | 65857,9 | 42931,0 | 60446,0 | 65798,9 | 112230,5 | 51841,1 | 48502,5 | 82211,1 | 130314,0 | 142276,7 |
| P29622 | Kallistatin | KAIN | SERPINA4 | 1213437,4 | 1090632,5 | 665431,2 | 1168226,4 | 644647,8 | 847998,4 | 1483292,2 | 1166614,2 | 741048,1 | 1063481,7 | 556323,8 | 680899,0 | 971770,7 | 555586,5 | 929931,4 | 1119929,6 | 862612,9 | 1011727,9 |
| P07357 | Complement component C8 alpha chain | CO8A | C8A | 511368,3 | 638397,0 | 422881,6 | 521025,8 | 598397,4 | 327359,8 | 374638,3 | 462108,7 | 399134,6 | 382686,1 | 328637,8 | 324542,1 | 480790,0 | 452684,4 | 473075,5 | 494403,3 | 283902,4 | 346743,3 |
| P02538 | Keratin, type II cytoskeletal 6A | K2C6A | KRT6A | 392033,8 | 100951,4 | 55952,9 | 194052,0 | 35493,2 | 26572,1 | 190886,2 | 47850,0 | 123797,6 | 61150,0 | 175898,1 | 38289,2 | 178114,5 | 168544,1 | 222276,6 | 89882,6 | 173761,8 | 30388,0 |
| P07225 | Vitamin K-dependent protein S | PROS | PROS1 | 558465,6 | 390465,4 | 582343,8 | 591497,7 | 437194,1 | 466266,7 | 532144,3 | 472665,2 | 514125,0 | 334673,5 | 425064,6 | 402610,3 | 407694,0 | 560325,9 | 437353,2 | 343959,1 | 349290,1 | 259301,6 |
| Q16610 | Extracellular matrix protein 1 | ECM1 | ECM1 | 139887,1 | 129673,8 | 270599,0 | 204544,7 | 231642,3 | 132265,7 | 106986,5 | 86460,9 | 143941,4 | 146698,5 | 75186,6 | 146301,3 | 216386,1 | 80725,5 | 119243,1 | 143454,8 | 156256,1 | 102237,6 |
| P02649 | Apolipoprotein E | APOE | APOE | 4528736,1 | 2528358,3 | 5829107,6 | 2965025,4 | 3215445,0 | 5560203,1 | 2224901,0 | 3853789,3 | 5763202,6 | 4479023,8 | 3876326,7 | 2779483,4 | 2856421,2 | 2493489,7 | 4618702,1 | 2527446,6 | 3914135,7 | 2396791,6 |
| P05543 | Thyroxine-binding globulin | THBG | SERPINA7 | 356799,3 | 334667,6 | 137258,2 | 433709,8 | 444457,5 | 501449,7 | 211377,6 | 86542,4 | 394187,4 | 349453,2 | 383456,5 | 117091,4 | 350188,7 | 389510,0 | 139422,3 | 524546,9 | 77832,6 | 66474,9 |
| P35858 | Insulin-like growth factor-binding protein complex acid labile subunit | ALS | IGFALS | 1483671,3 | 566655,9 | 978874,9 | 779644,5 | 547284,8 | 823314,6 | 1390442,9 | 835273,0 | 723413,8 | 717446,7 | 504400,2 | 472440,7 | 445495,5 | 837744,2 | 688987,2 | 602571,8 | 1157215,6 | 1041037,2 |
| P08779 | Keratin, type I cytoskeletal 16 | K1C16 | KRT16 | 567097,6 | 123754,7 | 91760,3 | 154998,0 | 32761,2 | 44379,1 | 268219,7 | 35091,8 | 73332,1 | 73738,4 | 38334,9 | 65703,1 | 89448,3 | 70358,2 | 216765,3 | 53803,4 | 53919,8 | 29238,8 |
| P22792 | Carboxypeptidase N subunit 2 | CPN2 | CPN2 | 888479,6 | 689928,1 | 667509,8 | 1065042,6 | 850238,4 | 883374,5 | 578994,2 | 607367,4 | 716335,0 | 734805,8 | 849337,9 | 370511,6 | 597765,9 | 961882,3 | 669664,8 | 709572,6 | 854233,5 | 494078,7 |
| O00391 | Sulfhydryl oxidase 1 | QSOX1 | QSOX1 | 81845,5 | 91870,9 | 75468,6 | 98744,6 | 82606,8 | 84353,9 | 80874,7 | 137100,5 | 61892,8 | 116173,1 | 122272,8 | 45157,9 | 123552,2 | 84440,5 | 90855,9 | 84930,2 | 158139,1 | 88767,3 |
| P01871 | Immunoglobulin heavy constant mu | IGHM | IGHM | 6458,3 | 7473,5 | 10481,9 | 1674,7 | 3097,0 | 12772,9 | 8389,8 | 4534,7 | 9718,2 | 4329,8 | 3234,1 | 7156,8 | 5115,5 | 6908,6 | 6754,6 | 7547,6 | 9751,6 | 7145,8 |
| P03951 | Coagulation factor XI | FA11 | F11 | 122038,0 | 87599,7 | 144962,1 | 182742,1 | 134728,0 | 168838,2 | 134879,5 | 130803,7 | 126051,1 | 60434,1 | 126407,4 | 110389,7 | 96515,2 | 159630,5 | 118317,9 | 127548,5 | 111452,7 | 94108,8 |
| Q92954 | Proteoglycan 4 | PRG4 | PRG4 | 166857,1 | 139057,8 | 121086,2 | 207435,5 | 298761,5 | 234066,1 | 147220,9 | 224639,0 | 125413,0 | 139102,2 | 167458,7 | 138612,6 | 140546,3 | 212227,9 | 188368,8 | 259324,9 | 143424,0 | 192569,2 |
| P80108 | Phosphatidylinositol-glycan-specific phospholipase D | PHLD | GPLD1 | 618633,9 | 386621,7 | 486629,2 | 695069,1 | 434802,4 | 442604,1 | 394532,8 | 388885,8 | 443405,4 | 391461,3 | 475184,8 | 262358,4 | 340502,6 | 476104,0 | 404295,8 | 341484,2 | 311683,6 | 366825,1 |
| P02753 | Retinol-binding protein 4 | RET4 | RBP4 | 37678,1 | 22080,2 | 101880,5 | 70595,9 | 32991,5 | 78963,3 | 27944,4 | 52450,0 | 55233,2 | 25480,9 | 69488,2 | 72295,3 | 52924,1 | 70309,1 | 48946,7 | 59833,4 | 69534,1 | 59098,9 |
| P13796 | Plastin-2 | PLSL | LCP1 | 141461,6 | 70529,9 | 107980,1 | 171540,4 | 198831,8 | 127577,3 | 211636,3 | 177447,5 | 69784,1 | 88231,4 | 97329,6 | 136814,0 | 178145,2 | 76523,3 | 131085,0 | 81289,5 | 83093,2 | 161069,0 |
| Q96KN2 | Beta-Ala-His dipeptidase | CNDP1 | CNDP1 | 135805,1 | 144241,5 | 148151,3 | 124478,2 | 79379,5 | 106864,7 | 118006,3 | 158721,2 | 115136,1 | 103702,3 | 148445,0 | 108794,5 | 85235,5 | 124556,9 | 114950,1 | 137124,1 | 101620,3 | 126971,6 |
| Q14520 | Hyaluronan-binding protein 2 | HABP2 | HABP2 | 522486,2 | 365505,3 | 454569,8 | 398151,0 | 375553,8 | 539244,4 | 432712,6 | 484777,5 | 348110,8 | 352713,7 | 492173,0 | 341981,1 | 240199,1 | 394052,7 | 349786,2 | 313473,2 | 330338,3 | 395532,9 |
| P22105 | Tenascin-X | TENX | TNXB | 214247,6 | 186432,4 | 104758,6 | 168275,1 | 231249,4 | 168076,6 | 143876,7 | 69173,5 | 241460,6 | 229589,4 | 160859,4 | 94453,8 | 164992,3 | 105231,6 | 150106,5 | 244633,8 | 153505,8 | 171716,8 |
| P02743 | Serum amyloid P-component | SAMP | APCS | 831538,2 | 743562,6 | 638309,8 | 700601,7 | 849431,8 | 604511,4 | 704185,2 | 667500,6 | 661349,2 | 470348,4 | 943094,1 | 687207,7 | 633432,0 | 661489,0 | 734108,2 | 879045,4 | 242898,7 | 795483,1 |
| P13647 | Keratin, type II cytoskeletal 5 | K2C5 | KRT5 | 135126,7 | 130093,2 | 164689,1 | 269943,3 | 89326,6 | 87916,4 | 185648,8 | 105297,8 | 102475,7 | 98815,6 | 78575,0 | 341373,6 | 129917,0 | 131475,3 | 50317,1 | 93436,0 | 99366,1 | 99629,7 |
| Q04756 | Hepatocyte growth factor activator | HGFA | HGFAC | 80893,6 | 91888,5 | 127663,2 | 55955,6 | 69445,8 | 56592,7 | 63227,2 | 83548,6 | 63796,2 | 68828,8 | 41487,6 | 73771,9 | 53670,3 | 38405,2 | 77615,3 | 56035,1 | 50536,7 | 73340,5 |
| P05452 | Tetranectin | TETN | CLEC3B | 86141,2 | 147648,0 | 109513,8 | 110308,4 | 119414,4 | 129171,2 | 86994,1 | 99492,9 | 79704,8 | 133768,5 | 78059,9 | 93976,5 | 87286,0 | 68248,8 | 85339,8 | 107896,8 | 130995,7 | 80461,0 |
| P02741 | C-reactive protein | CRP | CRP | 18299,5 | 12500,7 | 13135,9 | 6502,7 | 12681,6 | 8605,4 | 24498,6 | 19257,3 | 3930,3 | 9498,4 | 18858,7 | 19308,7 | 10958,6 | 5362,9 | 10849,2 | 23562,6 | 11178,8 | 10165,3 |
| Q96IY4 | Carboxypeptidase B2 | CBPB2 | CPB2 | 526105,7 | 546185,1 | 565788,4 | 820881,8 | 929470,6 | 806286,0 | 425203,2 | 544461,5 | 613922,1 | 525120,5 | 671474,3 | 433248,4 | 600124,2 | 662909,3 | 710504,8 | 491436,0 | 615796,8 | 599486,9 |
| P51884 | Lumican | LUM | LUM | 458531,5 | 789405,5 | 677704,5 | 520430,8 | 688107,1 | 506701,3 | 393812,1 | 478201,8 | 521460,6 | 546863,2 | 366426,7 | 568556,8 | 361282,0 | 428126,4 | 727223,5 | 390318,0 | 541660,1 | 312794,0 |
| P08185 | Corticosteroid-binding globulin | CBG | SERPINA6 | 766319,9 | 807746,8 | 589150,2 | 765905,6 | 900250,6 | 390151,5 | 421064,8 | 458271,2 | 739118,7 | 643537,9 | 653194,1 | 747563,9 | 746303,0 | 999130,5 | 961729,8 | 1051766,3 | 588128,5 | 1162433,4 |
| P06276 | Cholinesterase | CHLE | BCHE | 184989,9 | 168223,1 | 203455,4 | 214881,1 | 167113,1 | 257143,0 | 129376,7 | 98007,9 | 203772,4 | 188019,5 | 123653,3 | 211429,5 | 135107,6 | 219662,2 | 127750,4 | 175914,5 | 135912,6 | 151797,1 |
| P02750 | Leucine-rich alpha-2-glycoprotein | A2GL | LRG1 | 580756,2 | 507194,6 | 718884,2 | 489717,9 | 470158,6 | 1087610,2 | 215470,8 | 304277,5 | 939022,5 | 229532,6 | 133625,2 | 458347,5 | 413501,0 | 213750,1 | 227753,7 | 415453,9 | 405683,9 | 353326,6 |
| Q9UK55 | Protein Z-dependent protease inhibitor | ZPI | SERPINA10 | 165949,5 | 161073,3 | 123474,5 | 177346,2 | 148363,1 | 133386,1 | 121489,5 | 108914,4 | 99817,6 | 117386,1 | 115420,0 | 125818,0 | 122843,2 | 152629,7 | 157413,5 | 95669,0 | 133455,8 | 97957,4 |
| P07360 | Complement component C8 gamma chain | CO8G | C8G | 248726,6 | 204150,2 | 159944,7 | 227950,3 | 301385,2 | 171462,2 | 191739,9 | 205979,4 | 177490,5 | 167826,0 | 156848,5 | 199167,4 | 279731,1 | 248698,5 | 209990,8 | 202075,9 | 137294,1 | 138925,5 |
| Q15582 | Transforming growth factor-beta-induced protein ig-h3 | BGH3 | TGFBI | 104633,5 | 68890,9 | 73613,3 | 78826,4 | 98522,7 | 71196,8 | 66989,0 | 116050,5 | 63897,1 | 45671,2 | 47927,4 | 48035,8 | 51227,8 | 50654,7 | 72774,9 | 84692,1 | 58211,5 | 72733,8 |
| P14923 | Junction plakoglobin | PLAK | JUP | 74838,2 | 91163,3 | 91421,9 | 82139,7 | 92271,2 | 87424,3 | 67093,0 | 92791,7 | 88648,4 | 98746,8 | 71115,2 | 103759,4 | 74706,6 | 113278,6 | 109311,2 | 60137,1 | 85709,6 | 42905,1 |
| P00742 | Coagulation factor X | FA10 | F10 | 51496,0 | 98265,8 | 106205,9 | 108907,2 | 88674,0 | 67531,8 | 57658,4 | 46122,5 | 56974,6 | 39649,1 | 103818,2 | 69092,1 | 45231,6 | 49080,4 | 31433,5 | 48288,8 | 61056,4 | 87684,1 |
| P48740 | Mannan-binding lectin serine protease 1 | MASP1 | MASP1 | 239570,0 | 216599,1 | 314725,1 | 222815,3 | 230790,6 | 257929,5 | 232447,4 | 223036,6 | 236840,8 | 222343,8 | 184221,8 | 168533,4 | 190942,4 | 207465,3 | 194832,5 | 163469,3 | 163291,8 | 201422,9 |
| P23142 | Fibulin-1 | FBLN1 | FBLN1 | 97682,5 | 178679,6 | 306523,8 | 218503,6 | 224737,5 | 255514,1 | 173700,6 | 218360,5 | 215452,2 | 174096,4 | 126873,7 | 227224,6 | 157830,5 | 125057,8 | 213640,5 | 160740,7 | 174849,8 | 158906,9 |
| P98160 | Basement membrane-specific heparan sulfate proteoglycan core protein | PGBM | HSPG2 | 16043,3 | 23644,7 | 87774,3 | 37454,7 | 39716,6 | 31166,3 | 27524,5 | 71469,1 | 33414,6 | 34738,5 | 30729,1 | 54439,5 | 28827,0 | 39557,6 | 28765,6 | 35299,0 | 56119,0 | 29344,4 |
| P15144 | Aminopeptidase N | AMPN | ANPEP | 37918,3 | 23040,3 | 64943,5 | 74306,2 | 45755,4 | 21946,7 | 31000,5 | 88905,7 | 29136,4 | 32496,3 | 26469,1 | 129470,1 | 57879,2 | 39799,4 | 32269,5 | 57539,3 | 71924,9 | 61314,6 |
| Q12805 | EGF-containing fibulin-like extracellular matrix protein 1 | FBLN3 | EFEMP1 | 69476,8 | 45867,2 | 71264,6 | 57474,1 | 75448,8 | 101867,3 | 68977,9 | 56576,0 | 83868,8 | 66493,7 | 59997,3 | 29021,8 | 49074,1 | 53205,1 | 70922,6 | 54216,3 | 56765,4 | 42401,2 |
| P60709 | Actin, cytoplasmic 1 | ACTB | ACTB | 66683,3 | 46789,0 | 20179,9 | 22834,0 | 62305,2 | 30221,5 | 21735,4 | 25744,7 | 35850,8 | 17994,9 | 19237,8 | 33129,2 | 51485,2 | 42171,8 | 28161,1 | 43064,5 | 22861,9 | 24293,2 |
| P27169 | Serum paraoxonase/arylesterase 1 | PON1 | PON1 | 168138,1 | 65866,9 | 63706,9 | 132661,1 | 117347,1 | 80218,4 | 69594,8 | 114543,7 | 129484,5 | 25549,3 | 34009,8 | 96400,1 | 123118,9 | 50183,4 | 31075,5 | 59784,1 | 77766,8 | 102250,4 |
| Q86YZ3 | Hornerin | HORN | HRNR | 29210,5 | 19109,8 | 12758,3 | 47137,7 | 15379,9 | 25345,3 | 26322,4 | 12925,9 | 14498,8 | 33195,5 | 19509,8 | 20034,0 | 46368,2 | 25274,2 | 22500,7 | 27326,5 | 28693,2 | 25384,9 |
| P02746 | Complement C1q subcomponent subunit B | C1QB | C1QB | 218937,8 | 229378,7 | 139028,1 | 182068,8 | 252595,0 | 100787,9 | 111536,3 | 177366,3 | 150480,9 | 92690,5 | 163358,4 | 112340,5 | 257001,0 | 149338,8 | 272910,1 | 97045,2 | 56631,0 | 66351,7 |
| P05154 | Plasma serine protease inhibitor | IPSP | SERPINA5 | 303239,8 | 168609,1 | 266444,0 | 301185,4 | 131553,1 | 278085,8 | 324543,9 | 202231,6 | 232513,2 | 299973,9 | 301845,1 | 192018,9 | 233917,4 | 270910,8 | 190676,7 | 319690,5 | 230260,8 | 149615,1 |
| P49747 | Cartilage oligomeric matrix protein | COMP | COMP | 36959,5 | 35464,2 | 40069,5 | 52944,0 | 58431,8 | 31202,8 | 35037,1 | 69349,8 | 48624,0 | 33384,8 | 33146,8 | 60027,9 | 45807,2 | 41560,9 | 34510,7 | 58637,4 | 35760,1 | 44802,2 |
| P68871 | Hemoglobin subunit beta | HBB | HBB | 9806,3 | 5428,2 | 9354,3 | 13357,5 | 5508,0 | 10269,6 | 3468,2 | 26632,7 | 38259,9 | 25822,9 | 1584,1 | 21068,4 | 2998,5 | 7887,2 | 7850,7 | 8810,5 | 38631,9 | 16851,2 |
| P43251 | Biotinidase | BTD | BTD | 232882,7 | 362272,2 | 328068,1 | 201372,3 | 248813,4 | 175751,2 | 158253,5 | 120778,7 | 128646,4 | 139669,0 | 144712,0 | 319749,0 | 176496,3 | 143130,1 | 160661,7 | 127758,0 | 173792,7 | 119038,2 |
| P18428 | Lipopolysaccharide-binding protein | LBP | LBP | 171552,2 | 74525,6 | 104294,2 | 247186,1 | 110105,0 | 185924,7 | 62852,2 | 37890,3 | 162839,7 | 50097,7 | 184648,4 | 38522,2 | 91387,1 | 356726,4 | 70529,7 | 174036,5 | 60813,0 | 44063,4 |
| P22352 | Glutathione peroxidase 3 | GPX3 | GPX3 | 130104,3 | 123113,9 | 89419,9 | 135525,7 | 103647,7 | 42317,2 | 97643,3 | 72664,9 | 65962,7 | 74475,8 | 71320,2 | 39100,3 | 232096,8 | 113437,8 | 101535,5 | 161471,6 | 83951,7 | 76866,1 |
| P0DOX8 | Immunoglobulin lambda-1 light chain | IGL1 | 1 SV | 754628,0 | 1382430,6 | 457219,5 | 181098,2 | 1411551,4 | 1702329,7 | 406570,1 | 914764,1 | 423906,5 | 157009,6 | 1274214,3 | 224710,3 | 465821,7 | 1114834,9 | 882658,1 | 1094682,8 | 142451,5 | 622137,6 |
| P02533 | Keratin, type I cytoskeletal 14 | K1C14 | KRT14 | 204547,5 | 145498,4 | 70350,6 | 208630,9 | 73134,2 | 59880,5 | 246750,1 | 52828,0 | 59168,0 | 83458,7 | 27315,2 | 80818,3 | 194214,6 | 69992,9 | 104322,7 | 59481,7 | 44676,4 | 55647,3 |
| P15169 | Carboxypeptidase N catalytic chain | CBPN | CPN1 | 313390,6 | 230803,1 | 298175,2 | 320879,8 | 303205,0 | 332833,7 | 275593,9 | 268360,8 | 232073,3 | 236773,1 | 253103,0 | 194212,8 | 246556,4 | 313618,8 | 218049,2 | 236285,0 | 301868,2 | 172116,5 |
| P00740 | Coagulation factor IX | FA9 | F9 | 259127,3 | 272412,8 | 252003,0 | 327301,4 | 291511,1 | 448742,7 | 278431,6 | 213950,3 | 279810,2 | 197716,5 | 196697,5 | 161421,9 | 346953,0 | 284293,8 | 336198,1 | 243118,9 | 199844,5 | 214751,1 |
| P12111 | Collagen alpha-3(VI) chain | CO6A3 | COL6A3 | 26030,0 | 31537,8 | 24421,1 | 18818,0 | 34633,5 | 26091,7 | 26392,7 | 44224,1 | 23250,0 | 24281,6 | 30555,2 | 24943,4 | 33894,6 | 18711,6 | 30214,0 | 54116,7 | 43253,2 | 23054,6 |
| P01876 | Immunoglobulin heavy constant alpha 1 | IGHA1 | IGHA1 | 90087,6 | 261544,6 | 254071,8 | 84966,0 | 198987,9 | 407879,1 | 58013,7 | 147316,9 | 408552,5 | 73763,2 | 88364,4 | 66898,0 | 62213,4 | 84013,2 | 106610,7 | 74747,2 | 62883,1 | 83694,4 |
| P09172 | Dopamine beta-hydroxylase | DOPO | DBH | 97922,4 | 47526,4 | 76550,7 | 57023,5 | 66097,6 | 49074,1 | 103232,8 | 58160,0 | 32472,9 | 49633,1 | 60778,4 | 81511,9 | 59587,9 | 42563,2 | 45426,6 | 48923,9 | 50937,8 | 32654,9 |
| Q9BXR6 | Complement factor H-related protein 5 | FHR5 | CFHR5 | 52139,6 | 60680,2 | 27240,9 | 57156,7 | 52815,6 | 64261,6 | 36918,4 | 50416,3 | 76667,8 | 13246,1 | 41560,6 | 28333,0 | 54188,4 | 54197,2 | 50626,6 | 44951,2 | 31572,0 | 13198,6 |
| Q9UGM5 | Fetuin-B | FETUB | FETUB | 165151,5 | 104517,0 | 134080,9 | 135619,0 | 129688,8 | 96955,8 | 78746,6 | 88490,1 | 101417,6 | 89413,6 | 141607,4 | 63715,4 | 104852,4 | 102175,2 | 111454,3 | 184054,9 | 53950,8 | 184801,4 |
| P04278 | Sex hormone-binding globulin | SHBG | SHBG | 104174,3 | 141641,7 | 139596,4 | 136563,6 | 168602,7 | 167241,6 | 95379,8 | 145088,3 | 92263,5 | 101338,9 | 47163,4 | 79442,3 | 98258,8 | 45808,1 | 61898,1 | 170340,9 | 118190,0 | 319319,8 |
| P0C0L4 | Complement C4-A | CO4A | C4A | 560405,7 | 315021,7 | 267653,0 | 476220,4 | 520521,3 | 325968,6 | 368592,1 | 373712,8 | 719770,5 | 483217,0 | 1063230,3 | 263488,6 | 598214,7 | 1167197,0 | 439633,9 | 1439161,7 | 320097,0 | 282923,3 |
| Q02413 | Desmoglein-1 | DSG1 | DSG1 | 118393,3 | 80331,5 | 10351,7 | 29099,9 | 93740,2 | 42412,2 | 31557,2 | 74864,9 | 70041,3 | 16011,1 | 24015,7 | 22193,0 | 39673,5 | 12073,7 | 88541,6 | 75557,1 | 25888,8 | 78547,6 |
| O95445 | Apolipoprotein M | APOM | APOM | 275843,5 | 273988,9 | 169762,1 | 353795,6 | 308684,8 | 325534,2 | 92175,9 | 244672,0 | 274200,3 | 192224,9 | 139346,9 | 195785,8 | 132580,6 | 220487,9 | 253628,3 | 196432,5 | 199687,6 | 152767,1 |
| P02747 | Complement C1q subcomponent subunit C | C1QC | C1QC | 610099,3 | 699189,8 | 827782,1 | 990890,1 | 1079154,3 | 776585,5 | 769043,8 | 863753,2 | 647546,0 | 644373,7 | 502223,5 | 700643,3 | 1104499,2 | 824435,8 | 817086,6 | 726885,7 | 891800,5 | 691621,1 |
| O00533 | Neural cell adhesion molecule L1-like protein | NCHL1 | CHL1 | 27393,4 | 4006,3 | 16163,2 | 12970,3 | 4096,8 | 8918,4 | 1477,8 | 8584,4 | 1472,7 | 11848,8 | 6559,2 | 2793,5 | 4499,1 | 10699,4 | 11259,2 | 11111,3 | 12719,6 | 4768,0 |
| P22891 | Vitamin K-dependent protein Z | PROZ | PROZ | 44488,6 | 24566,3 | 63465,0 | 66880,1 | 19805,8 | 43152,7 | 38743,7 | 39444,5 | 19402,9 | 16282,1 | 22042,2 | 11219,2 | 37836,6 | 25272,4 | 26561,4 | 14419,1 | 66722,5 | 8459,2 |
| Q13822 | Ectonucleotide pyrophosphatase/phosphodiesterase family member 2 | ENPP2 | ENPP2 | 26471,6 | 24086,1 | 86764,5 | 39979,9 | 39435,2 | 21439,4 | 55968,6 | 81346,1 | 47921,7 | 32264,3 | 35516,4 | 23600,4 | 77854,3 | 33200,5 | 35318,7 | 32174,9 | 29730,8 | 28161,9 |
| Q08380 | Galectin-3-binding protein | LG3BP | LGALS3BP | 102011,8 | 102375,2 | 74390,0 | 109908,1 | 128507,8 | 61929,9 | 151341,1 | 75029,8 | 59958,3 | 54667,6 | 27646,0 | 68558,9 | 74336,3 | 59929,8 | 54951,7 | 64873,2 | 66110,0 | 88344,9 |
| P08571 | Monocyte differentiation antigen CD14 | CD14 | CD14 | 188555,5 | 112207,2 | 174028,4 | 339136,7 | 299936,4 | 262916,3 | 151300,6 | 153518,1 | 131857,4 | 164459,7 | 86094,2 | 150437,2 | 253099,2 | 243067,7 | 284292,2 | 95521,8 | 131406,4 | 150669,9 |
| Q9NQ79 | Cartilage acidic protein 1 | CRAC1 | CRTAC1 | 35723,6 | 103597,9 | 148535,7 | 75157,1 | 54088,2 | 71212,6 | 40795,3 | 68360,5 | 28806,4 | 53851,6 | 52145,4 | 107356,5 | 75465,7 | 72059,0 | 45691,9 | 57665,8 | 37154,6 | 62795,2 |
| P24821 | Tenascin | TENA | TNC | 6738,5 | 27028,6 | 13208,3 | 18293,0 | 16592,1 | 14677,4 | 59885,8 | 25779,3 | 16897,3 | 9970,1 | 17348,7 | 15024,2 | 18424,8 | 7894,7 | 19063,9 | 21431,2 | 11647,1 | 33777,2 |
| Q9NZP8 | Complement C1r subcomponent-like protein | C1RL | C1RL | 116715,2 | 112741,6 | 88319,1 | 158821,0 | 163764,8 | 177884,1 | 81794,1 | 82812,3 | 122999,2 | 120088,3 | 104988,9 | 57769,0 | 90280,2 | 193582,1 | 165606,1 | 108767,0 | 85829,7 | 107983,2 |
| O00187 | Mannan-binding lectin serine protease 2 | MASP2 | MASP2 | 62909,2 | 37900,2 | 51250,2 | 82509,6 | 63648,6 | 65150,1 | 46079,6 | 52991,5 | 43568,8 | 37814,4 | 89500,9 | 67076,6 | 55905,8 | 47975,6 | 33760,5 | 48102,3 | 64572,9 | 48243,7 |
| P17936 | Insulin-like growth factor-binding protein 3 | IBP3 | IGFBP3 | 35014,6 | 17937,0 | 17906,1 | 28642,5 | 18823,6 | 9055,6 | 161402,3 | 20547,6 | 30006,2 | 21436,3 | 9477,6 | 34347,1 | 39721,5 | 16367,3 | 25944,4 | 22568,6 | 23846,9 | 16895,1 |
| O75636 | Ficolin-3 | FCN3 | FCN3 | 322511,5 | 108581,3 | 56374,5 | 469424,0 | 104229,2 | 125232,0 | 203320,8 | 33205,0 | 138141,7 | 207031,0 | 172059,6 | 103350,7 | 238313,8 | 114455,9 | 184437,8 | 130230,2 | 269476,3 | 79518,4 |
| Q9UHG3 | Prenylcysteine oxidase 1 | PCYOX | PCYOX1 | 101333,3 | 82688,7 | 67487,1 | 78346,7 | 85604,9 | 93278,7 | 99095,6 | 86893,0 | 57910,4 | 72385,4 | 99308,6 | 119838,7 | 64996,5 | 78829,0 | 130137,7 | 89342,9 | 83151,9 | 132142,6 |
| P32119 | Peroxiredoxin-2 | PRDX2 | PRDX2 | 30359,6 | 25633,9 | 21151,4 | 25280,4 | 6554,5 | 11102,0 | 21368,7 | 10168,6 | 24365,1 | 7546,4 | 126649,8 | 12137,3 | 26931,7 | 18546,1 | 13733,1 | 14523,0 | 4716,8 | 17888,0 |
| P01859 | Immunoglobulin heavy constant gamma 2 | IGHG2 | IGHG2 | 1989,3 | 2200,1 | 2088,9 | 1494,8 | 3666,8 | 1639,4 | 2493,1 | 2112,1 | 4776,4 | 1566,1 | 1167,0 | 2689,3 | 1328,4 | 1802,2 | 809,7 | 1482,7 | 1986,5 | 635,5 |
| P02652 | Apolipoprotein A-II | APOA2 | APOA2 | 65101,9 | 128892,4 | 39525,7 | 63251,5 | 60438,3 | 84830,2 | 34726,2 | 100150,9 | 111373,7 | 78041,5 | 105061,0 | 37943,9 | 35901,0 | 76286,9 | 60640,9 | 136826,9 | 63086,8 | 84304,0 |
| P35443 | Thrombospondin-4 | TSP4 | THBS4 | 44244,6 | 19157,3 | 21281,3 | 22177,7 | 21924,2 | 21103,2 | 27067,7 | 51328,6 | 13784,8 | 35456,6 | 34921,8 | 31680,4 | 21176,9 | 17496,0 | 27632,0 | 16423,5 | 28670,6 | 40801,4 |
| Q5T749 | Keratinocyte proline-rich protein | KPRP | KPRP | 75833,9 | 45959,0 | 59348,1 | 81834,4 | 68348,1 | 43544,8 | 117573,2 | 65888,8 | 36848,2 | 33521,2 | 18392,9 | 33160,0 | 75428,4 | 52747,1 | 46637,5 | 45223,5 | 26166,6 | 60664,7 |
| P33908 | Mannosyl-oligosaccharide 1,2-alpha-mannosidase IA | MA1A1 | MAN1A1 | 22907,9 | 69632,9 | 72367,1 | 143589,9 | 110331,6 | 35443,0 | 37204,1 | 164576,6 | 71818,1 | 27546,5 | 48236,8 | 33096,4 | 48325,4 | 48313,4 | 54659,5 | 138626,9 | 129704,2 | 45899,5 |
| P00746 | Complement factor D | CFAD | CFD | 53774,4 | 45091,4 | 44251,8 | 40713,8 | 46728,0 | 68375,1 | 22632,1 | 23132,7 | 54001,7 | 70993,6 | 202250,9 | 31943,3 | 9562,5 | 290378,4 | 49227,0 | 529442,5 | 40484,0 | 42927,6 |
| P19013 | Keratin, type II cytoskeletal 4 | K2C4 | KRT4 | 47224,8 | 48664,9 | 56452,9 | 49885,6 | 39370,4 | 46385,5 | 28533,7 | 162164,4 | 39971,5 | 69597,3 | 43034,4 | 47178,1 | 96656,4 | 16520,8 | 12621,3 | 36837,4 | 55637,9 | 28042,9 |
| P27918 | Properdin | PROP | CFP | 125850,8 | 91108,0 | 138018,4 | 201829,0 | 96539,4 | 136247,6 | 126601,8 | 101192,0 | 86287,7 | 180321,8 | 182281,1 | 165032,2 | 110628,1 | 138061,2 | 164816,0 | 105381,1 | 161132,3 | 110896,6 |
| Q86VB7 | Scavenger receptor cysteine-rich type 1 protein M130 | C163A | CD163 | 15143,7 | 42521,3 | 63161,6 | 24567,2 | 44288,8 | 26257,8 | 24021,6 | 38195,0 | 17617,0 | 46482,4 | 25437,2 | 29692,9 | 45302,9 | 32676,6 | 35585,1 | 16868,0 | 23965,0 | 12690,1 |
| P02766 | Transthyretin | TTHY | TTR | 5712,3 | 1871,3 | 3265,3 | 4040,0 | 3810,6 | 8422,7 | 4995,5 | 15165,3 | 2842,8 | 4047,2 | 4649,2 | 4232,3 | 5258,4 | 2674,6 | 4029,7 | 6498,7 | 5933,0 | 3465,9 |
| O43866 | CD5 antigen-like | CD5L | CD5L | 91207,6 | 109282,8 | 108894,3 | 107421,0 | 126268,2 | 50685,7 | 62587,2 | 88693,3 | 116326,8 | 38360,9 | 20146,4 | 42128,6 | 65543,7 | 38429,1 | 38739,6 | 55395,5 | 79853,3 | 66804,2 |
| P02763 | Alpha-1-acid glycoprotein 1 | A1AG1 | ORM1 | 23563,9 | 23038,4 | 33679,9 | 47172,2 | 28941,7 | 34963,7 | 17538,5 | 24887,3 | 9239,5 | 13935,7 | 11852,3 | 11861,4 | 19663,4 | 24327,3 | 32872,2 | 11330,1 | 8857,5 | 17206,6 |
| P69905 | Hemoglobin subunit alpha | HBA | HBA1 | 11207,6 | 8859,6 | 37743,3 | 17433,2 | 12815,4 | 12133,7 | 22748,8 | 15493,9 | 11775,6 | 13167,3 | 4655,8 | 24823,8 | 9062,7 | 12197,5 | 21701,8 | 18914,6 | 28042,5 | 13614,6 |
| P02745 | Complement C1q subcomponent subunit A | C1QA | C1QA | 768902,4 | 745143,3 | 795535,5 | 1121951,0 | 1273909,0 | 1159319,7 | 691950,3 | 823888,7 | 881733,4 | 799426,6 | 567163,5 | 638840,8 | 974703,2 | 1089169,2 | 1283538,7 | 866815,1 | 573673,0 | 661819,5 |
| Q04695 | Keratin, type I cytoskeletal 17 | K1C17 | KRT17 | 274085,1 | 39855,2 | 29413,1 | 95775,6 | 51698,5 | 336313,1 | 78911,8 | 32989,2 | 76115,1 | 63964,1 | 45264,4 | 34532,7 | 85979,6 | 50494,2 | 72051,9 | 158267,9 | 39926,0 | 52563,5 |
| P69891 | Hemoglobin subunit gamma-1 | HBG1 | HBG1 | 3687,0 | 3455,6 | 2780,9 | 7336,8 | 649,1 | 5374,0 | 6959,1 | 12506,0 | 3381,1 | 7286,7 | 164,5 | 14039,7 | 4931,4 | 8579,1 | 8864,7 | 6239,5 | 7774,1 | 4596,5 |
| P11021 | Endoplasmic reticulum chaperone BiP | BIP | HSPA5 | 7234,0 | 4074,1 | 15907,7 | 6403,7 | 8269,1 | 13987,8 | 1473,1 | 15350,7 | 16269,0 | 7938,7 | 5139,5 | 30546,9 | 24430,8 | 2465,6 | 5522,2 | 1726,5 | 4043,1 | 2148,0 |
| P55058 | Phospholipid transfer protein | PLTP | PLTP | 18478,4 | 19863,8 | 35157,0 | 44437,0 | 21548,8 | 25326,6 | 15171,3 | 30110,8 | 43490,0 | 29689,8 | 25992,6 | 29598,0 | 25761,0 | 28554,4 | 26531,2 | 27582,5 | 26624,6 | 18996,8 |
| P49908 | Selenoprotein P | SEPP1 | SELENOP | 57656,1 | 62096,1 | 42649,8 | 72037,1 | 46229,0 | 48483,0 | 46896,8 | 40873,3 | 50858,1 | 51413,8 | 45565,0 | 75446,1 | 54316,7 | 39694,6 | 41585,6 | 49137,1 | 61821,7 | 52541,4 |
| P07996 | Thrombospondin-1 | TSP1 | THBS1 | 73995,7 | 27170,0 | 55773,1 | 180099,1 | 159715,1 | 44759,8 | 257621,4 | 43617,1 | 128845,1 | 47271,5 | 22370,9 | 61471,0 | 188224,8 | 41577,0 | 108555,8 | 99894,3 | 220040,9 | 229162,2 |
| Q07954 | Prolow-density lipoprotein receptor-related protein 1 | LRP1 | LRP1 | 1737,7 | 19069,2 | 9618,0 | 1418,2 | 1497,8 | 457,3 | 1422,7 | 3403,6 | 3294,4 | 1244,1 | 23260,7 | 1233,6 | 1517,1 | 726,5 | 9449,9 | 840,2 | 12113,4 | 7100,7 |
| P19320 | Vascular cell adhesion protein 1 | VCAM1 | VCAM1 | 18621,7 | 51876,3 | 9788,8 | 32704,3 | 21503,4 | 26685,3 | 17810,0 | 73063,3 | 17847,9 | 4998,4 | 12901,8 | 9920,5 | 11400,7 | 23760,1 | 20914,5 | 26801,2 | 11308,6 | 84499,5 |
| P36980 | Complement factor H-related protein 2 | FHR2 | CFHR2 | 71623,3 | 115036,0 | 169400,0 | 102429,7 | 151376,8 | 166925,5 | 137075,1 | 114705,0 | 166748,4 | 94665,7 | 103145,5 | 66947,5 | 106127,8 | 129686,9 | 88554,2 | 113306,9 | 78119,9 | 56186,1 |
| P36222 | Chitinase-3-like protein 1 | CH3L1 | CHI3L1 | 13852,4 | 61079,0 | 49643,0 | 42407,0 | 13367,1 | 7307,3 | 48204,3 | 14664,2 | 34781,2 | 8189,3 | 9009,0 | 47724,3 | 22342,4 | 16794,1 | 20728,1 | 38756,4 | 19268,8 | 45362,5 |
| P05090 | Apolipoprotein D | APOD | APOD | 62624,0 | 28834,8 | 74182,6 | 45149,5 | 66135,9 | 68878,3 | 57434,5 | 61597,9 | 91032,8 | 49861,1 | 27879,6 | 45894,1 | 61532,9 | 34708,9 | 50350,0 | 73772,9 | 35139,7 | 36354,5 |
| P13591 | Neural cell adhesion molecule 1 | NCAM1 | NCAM1 | 29541,3 | 29748,6 | 26362,5 | 33521,2 | 25575,7 | 15488,8 | 20512,6 | 30932,2 | 23055,1 | 27024,0 | 15075,9 | 60325,8 | 18400,3 | 76828,9 | 15974,7 | 16047,4 | 31877,7 | 30952,8 |
| P02655 | Apolipoprotein C-II | APOC2 | APOC2 | 98304,8 | 100538,0 | 119817,5 | 115947,1 | 132911,4 | 157580,3 | 60149,0 | 74903,0 | 172923,8 | 133560,5 | 148502,3 | 105262,3 | 72371,1 | 90413,4 | 143149,5 | 128636,6 | 188527,0 | 28743,5 |
| P33151 | Cadherin-5 | CADH5 | CDH5 | 39519,1 | 47704,5 | 48824,0 | 59658,5 | 46501,7 | 50932,8 | 318920,1 | 36055,5 | 42004,6 | 59660,0 | 86517,0 | 85527,4 | 40343,4 | 51793,2 | 55570,3 | 33714,3 | 39504,3 | 33640,8 |
| O14791 | Apolipoprotein L1 | APOL1 | APOL1 | 4262,7 | 5297,1 | 2340,6 | 5732,5 | 6921,0 | 8465,0 | 25073,9 | 6191,8 | 9526,7 | 2131,6 | 753,7 | 8794,5 | 6061,8 | 8572,3 | 3041,0 | 8328,6 | 4833,6 | 9957,5 |
| P07359 | Platelet glycoprotein Ib alpha chain | GP1BA | GP1BA | 16357,2 | 59118,1 | 41777,9 | 34520,8 | 76425,8 | 17270,3 | 9242,4 | 19508,8 | 59383,2 | 66863,5 | 22056,5 | 28525,2 | 33869,3 | 49691,4 | 15098,9 | 61914,4 | 77184,6 | 9692,4 |
| P04070 | Vitamin K-dependent protein C | PROC | PROC | 93164,6 | 86984,9 | 113383,3 | 93638,7 | 109124,9 | 99546,2 | 105678,6 | 115724,7 | 89149,7 | 72883,1 | 143259,1 | 104522,4 | 95476,1 | 120457,5 | 88032,9 | 80159,0 | 50738,5 | 96107,0 |
| Q6EMK4 | Vasorin | VASN | VASN | 9582,4 | 2434,9 | 3551,8 | 10268,6 | 5080,1 | 17718,2 | 4898,6 | 3217,6 | 5590,6 | 7012,8 | 5689,2 | 24649,4 | 8263,8 | 5237,5 | 3367,4 | 18612,3 | 9750,7 | 4627,3 |
| P13646 | Keratin, type I cytoskeletal 13 | K1C13 | KRT13 | 815047,2 | 651263,9 | 575969,8 | 870767,4 | 295199,9 | 342389,4 | 886982,5 | 444479,3 | 662293,0 | 634841,4 | 215157,6 | 503836,3 | 828056,2 | 614862,9 | 421796,8 | 309348,7 | 229804,6 | 310867,5 |
| P20851 | C4b-binding protein beta chain | C4BPB | C4BPB | 113525,7 | 115212,1 | 104360,3 | 134931,4 | 140043,7 | 179648,0 | 88406,2 | 78151,2 | 103362,2 | 106150,1 | 126225,7 | 78749,8 | 72448,8 | 129252,3 | 124236,8 | 34436,3 | 107630,3 | 40068,8 |
| Q9Y490 | Talin-1 | TLN1 | TLN1 | 1603,6 | 3548,0 | 851,6 | 1483,6 | 953,3 | 2164,7 | 1107,4 | 1742,2 | 914,6 | 1721,0 | 2306,5 | 1706,4 | 919,3 | 4560,7 | 402,1 | 618,6 | 5039,4 | 2400,2 |
| P54802 | Alpha-N-acetylglucosaminidase | ANAG | NAGLU | 99767,6 | 40959,7 | 57529,4 | 67387,9 | 22361,2 | 42243,3 | 52198,4 | 49524,2 | 62697,2 | 115065,6 | 18530,3 | 88083,9 | 53182,7 | 53621,8 | 121796,4 | 60860,1 | 74928,6 | 12495,5 |
| P80188 | Neutrophil gelatinase-associated lipocalin | NGAL | LCN2 | 8832,4 | 2305,3 | 2833,0 | 831,7 | 13552,5 | 9631,7 | 8218,2 | 6715,9 | 4303,9 | 2052,6 | 2817,6 | 2443,0 | 5348,5 | 1831,7 | 654,8 | 2368,6 | 3858,5 | 6698,6 |
| Q8NBP7 | Proprotein convertase subtilisin/kexin type 9 | PCSK9 | PCSK9 | 19594,9 | 13311,1 | 30292,2 | 25443,7 | 18545,4 | 15361,8 | 19219,7 | 61592,8 | 21604,2 | 22501,4 | 11214,9 | 17238,6 | 21314,0 | 16829,5 | 18454,9 | 15165,8 | 18074,7 | 29065,0 |
| P02656 | Apolipoprotein C-III | APOC3 | APOC3 | 166155,7 | 468077,9 | 236194,3 | 164574,0 | 144576,4 | 192583,4 | 114230,5 | 553901,6 | 164801,8 | 181889,7 | 475349,2 | 229192,8 | 409166,3 | 96270,2 | 172174,5 | 1010710,3 | 250464,4 | 442032,8 |
| P04180 | Phosphatidylcholine-sterol acyltransferase | LCAT | LCAT | 55162,6 | 65108,3 | 114391,2 | 91968,8 | 45278,2 | 105970,5 | 90820,7 | 94351,1 | 82510,4 | 85826,4 | 40671,8 | 55333,3 | 93428,9 | 46868,6 | 76235,5 | 82267,7 | 76950,2 | 85159,3 |
| P08709 | Coagulation factor VII | FA7 | F7 | 28182,0 | 28598,3 | 28246,5 | 46685,8 | 34379,2 | 18983,0 | 10419,5 | 39137,3 | 19192,4 | 19284,3 | 40071,9 | 35887,2 | 39040,2 | 39564,1 | 41683,9 | 53809,9 | 64545,4 | 47278,8 |
| P61626 | Lysozyme C | LYSC | LYZ | 55843,2 | 57441,2 | 37178,2 | 125303,6 | 173527,6 | 143481,2 | 82640,8 | 50894,9 | 25655,3 | 50313,1 | 37531,4 | 56252,0 | 112640,5 | 71460,7 | 71156,3 | 36641,8 | 33053,5 | 45199,0 |
| P31327 | Carbamoyl-phosphate synthase [ammonia], mitochondrial | CPSM | CPS1 | 35656,2 | 55333,5 | 38016,4 | 35609,7 | 1769,0 | 45900,1 | 36785,0 | 39649,0 | 46487,0 | 25335,7 | 26181,5 | 886,6 | 12535,6 | 27061,1 | 25448,9 | 1878,2 | 2383,2 | 3742,9 |
| P00915 | Carbonic anhydrase 1 | CAH1 | CA1 | 6045,9 | 14394,0 | 15178,2 | 4495,2 | 9760,6 | 11040,7 | 13491,4 | 8724,3 | 17564,9 | 15351,6 | 15317,1 | 15726,9 | 10909,4 | 28068,4 | 20569,8 | 15540,9 | 16354,3 | 10224,9 |
| P01880 | Immunoglobulin heavy constant delta | IGHD | IGHD | 94380,1 | 79754,4 | 77715,3 | 125317,0 | 82017,2 | 59665,1 | 81135,5 | 82876,7 | 50984,8 | 173518,2 | 90224,4 | 81219,0 | 214781,3 | 86449,3 | 116689,4 | 176435,2 | 102983,9 | 58301,7 |
| O95497 | Pantetheinase | VNN1 | VNN1 | 46233,8 | 1238,4 | 64829,7 | 11385,3 | 50758,5 | 7845,7 | 53099,1 | 26142,8 | 4075,4 | 26477,6 | 23432,4 | 12470,9 | 107344,8 | 20624,1 | 3407,2 | 28503,2 | 37124,9 | 30034,3 |
| P07355 | Annexin A2 | ANXA2 | ANXA2 | 26809,2 | 23261,6 | 29858,7 | 13554,2 | 36901,8 | 31705,4 | 50041,8 | 28079,0 | 26302,1 | 15886,8 | 17558,0 | 30515,8 | 45524,8 | 17375,3 | 18454,1 | 22856,4 | 14547,1 | 23501,3 |
| P04040 | Catalase | CATA | CAT | 15392,1 | 12612,9 | 8090,1 | 6794,5 | 8627,9 | 9149,6 | 8810,1 | 9467,6 | 11541,6 | 12330,8 | 7430,4 | 16491,8 | 6095,7 | 15605,9 | 13705,9 | 16295,3 | 7191,6 | 6797,3 |
| P05062 | Fructose-bisphosphate aldolase B | ALDOB | ALDOB | 5999,3 | 8241,2 | 19870,5 | 7364,9 | 15440,5 | 18387,0 | 9688,1 | 14101,1 | 3789,1 | 14730,5 | 18850,9 | 32400,2 | 42356,4 | 2113,7 | 14322,8 | 36089,8 | 50479,3 | 6747,7 |
| O95479 | GDH/6PGL endoplasmic bifunctional protein | G6PE | H6PD | 26477,7 | 17367,6 | 3279,1 | 5826,8 | 11528,5 | 19287,4 | 1286,1 | 21321,6 | 6982,5 | 25852,1 | 6756,4 | 9286,4 | 10076,5 | 21690,2 | 15283,8 | 11502,9 | 6371,6 | 31223,5 |
| P18206 | Vinculin | VINC | VCL | 16024,3 | 11274,4 | 12733,2 | 25207,2 | 24610,9 | 21366,9 | 12886,5 | 14034,6 | 7994,9 | 4851,6 | 107341,7 | 4605,7 | 7240,6 | 5968,2 | 15251,2 | 54249,8 | 13693,1 | 11279,2 |
| P02788 | Lactotransferrin | TRFL | LTF | 22351,6 | 23229,8 | 14916,4 | 34118,3 | 18359,5 | 47111,1 | 11395,3 | 17238,7 | 32647,2 | 8429,3 | 14759,5 | 15963,8 | 16023,3 | 6666,8 | 39027,1 | 19203,6 | 13894,7 | 39081,6 |
| Q16706 | Alpha-mannosidase 2 | MA2A1 | MAN2A1 | 2368,9 | 21624,0 | 8102,7 | 7504,0 | 5207,7 | 17906,4 | 3536,0 | 5470,8 | 4850,1 | 6956,2 | 10431,6 | 6353,8 | 6551,8 | 9094,2 | 30270,0 | 20766,9 | 8460,9 | 10963,9 |
| P01619 | Immunoglobulin kappa variable 3-20 | KV320 | IGKV3-20 | 534,0 | 702,8 | 697,5 | 381,4 | 628,5 | 923,8 | 559,8 | 775,0 | 564,8 | 1660,1 | 4541,2 | 1357,4 | 1634,8 | 913,0 | 667,8 | 373,7 | 1244,5 | 625,7 |
| Q8N1N4 | Keratin, type II cytoskeletal 78 | K2C78 | KRT78 | 803721,9 | 652011,8 | 63749,5 | 775078,7 | 365224,9 | 171619,3 | 785965,3 | 52375,2 | 242921,7 | 218902,6 | 181763,0 | 43040,3 | 872654,6 | 261142,2 | 29317,8 | 325080,1 | 97067,0 | 49651,5 |
| P11226 | Mannose-binding protein C | MBL2 | MBL2 | 3199,4 | 3783,1 | 14610,5 | 415,9 | 2184,3 | 24559,5 | 3299,4 | 1677,9 | 5335,9 | 19620,3 | 4735,9 | 6895,1 | 798,0 | 3349,7 | 24380,0 | 3985,6 | 1731,3 | 3157,1 |
| P14151 | L-selectin | LYAM1 | SELL | 19544,0 | 26162,3 | 28405,0 | 74538,3 | 69193,2 | 57059,9 | 25159,6 | 31978,1 | 16684,8 | 35568,2 | 23556,7 | 46486,7 | 64151,3 | 42361,6 | 43507,0 | 39078,5 | 22276,3 | 48477,2 |
| P54108 | Cysteine-rich secretory protein 3 | CRIS3 | CRISP3 | 5374,6 | 2815,6 | 6182,7 | 6930,3 | 4042,0 | 6380,2 | 11148,5 | 5851,7 | 5205,2 | 4599,5 | 1329,8 | 14634,1 | 5531,6 | 3800,1 | 2860,5 | 3902,7 | 3264,7 | 1348,3 |
| P35542 | Serum amyloid A-4 protein | SAA4 | SAA4 | 175594,1 | 96891,4 | 201711,6 | 177358,6 | 180868,3 | 181189,6 | 136918,7 | 136063,7 | 203343,7 | 149452,7 | 17076,2 | 113899,2 | 175489,1 | 204065,0 | 218696,4 | 158746,3 | 150879,3 | 92201,7 |
| P39060 | Collagen alpha-1(XVIII) chain | COIA1 | COL18A1 | 3437,2 | 1417,8 | 1628,6 | 7508,6 | 3950,2 | 2618,2 | 1681,0 | 1375,9 | 20025,8 | 4924,6 | 3576,6 | 9911,4 | 1344,8 | 4858,3 | 4150,3 | 24915,9 | 1381,0 | 3948,9 |
| Q5D862 | Filaggrin-2 | FILA2 | FLG2 | 60685,0 | 56383,0 | 8891,3 | 21390,2 | 6521,6 | 40017,2 | 36174,6 | 7619,9 | 7174,4 | 6642,2 | 13320,8 | 24535,9 | 58923,2 | 20339,6 | 7202,6 | 22791,8 | 13384,7 | 29409,2 |
| O14786 | Neuropilin-1 | NRP1 | NRP1 | 55407,4 | 32337,3 | 36116,8 | 38752,3 | 102916,9 | 27547,1 | 59836,5 | 36121,0 | 39816,8 | 78253,7 | 91709,3 | 38902,0 | 96541,0 | 40718,9 | 53181,6 | 38773,8 | 57223,3 | 44872,6 |
| P0DOY3 | Immunoglobulin lambda constant 3 | IGLC3 | IGLC3 | 5369079,0 | 9260450,7 | 9217976,0 | 5267203,9 | 8231428,5 | 8514060,8 | 4520414,3 | 6611227,4 | 10823459,7 | 5089646,7 | 1306033,9 | 5856070,5 | 2930394,6 | 4827744,1 | 6005053,8 | 4535464,6 | 5372301,3 | 5089901,9 |
| P61769 | Beta-2-microglobulin | B2MG | B2M | 415,9 | 506,7 | 260,4 | 250,5 | 193,3 | 427,5 | 929,7 | 883,0 | 1241,7 | 811,1 | 60,5 | 555,1 | 325,7 | 673,1 | 662,1 | 446,4 | 2101,5 | 1017,3 |
| P25787 | Proteasome subunit alpha type-2 | PSA2 | PSMA2 | 18074,5 | 16143,2 | 31539,6 | 23251,8 | 15428,5 | 9929,5 | 15325,1 | 3575,9 | 8442,0 | 6514,5 | 12247,3 | 13463,5 | 32091,9 | 13871,9 | 25789,5 | 26006,4 | 21342,9 | 3358,8 |
| Q12913 | Receptor-type tyrosine-protein phosphatase eta | PTPRJ | PTPRJ | 3453,7 | 4945,4 | 5834,2 | 1091,4 | 5432,0 | 8591,9 | 3218,4 | 1833,7 | 2567,8 | 2316,5 | 1332,7 | 8097,7 | 11819,0 | 2211,0 | 1862,7 | 2041,8 | 1297,2 | 3932,0 |
| Q12860 | Contactin-1 | CNTN1 | CNTN1 | 8740,7 | 116004,3 | 7561,6 | 123403,9 | 87294,4 | 52925,1 | 17265,8 | 9441,9 | 73695,0 | 132032,0 | 68921,8 | 183257,8 | 14855,6 | 82858,4 | 89547,1 | 13367,7 | 15068,3 | 8278,4 |
| Q13093 | Platelet-activating factor acetylhydrolase | PAFA | PLA2G7 | 18764,9 | 65944,1 | 9412,1 | 35453,4 | 42796,7 | 14134,8 | 32896,7 | 46264,7 | 19641,3 | 11069,7 | 7691,1 | 21438,8 | 29520,0 | 10036,1 | 8033,1 | 12486,6 | 17229,5 | 13444,8 |
| P05362 | Intercellular adhesion molecule 1 | ICAM1 | ICAM1 | 618,6 | 751,2 | 2688,6 | 6133,2 | 935,7 | 6594,2 | 159406,5 | 1454,0 | 2008,7 | 3019,7 | 3393,3 | 6827,9 | 2681,6 | 9057,6 | 10736,4 | 1268,1 | 14832,5 | 2915,0 |
| Q7Z794 | Keratin, type II cytoskeletal 1b | K2C1B | KRT77 | 126602,2 | 89956,7 | 71646,3 | 64927,4 | 31991,4 | 57892,2 | 156971,6 | 62517,4 | 29930,8 | 53880,6 | 24432,5 | 80703,9 | 115745,0 | 56703,5 | 61092,5 | 50315,6 | 64714,3 | 60471,9 |
| P43121 | Cell surface glycoprotein MUC18 | MUC18 | MCAM | 15038,9 | 23065,5 | 45867,9 | 25782,1 | 11037,0 | 38871,7 | 26011,7 | 18808,6 | 37760,8 | 51567,1 | 13854,2 | 66832,4 | 30106,8 | 10927,3 | 36081,5 | 50004,0 | 24460,9 | 10785,8 |
| P07333 | Macrophage colony-stimulating factor 1 receptor | CSF1R | CSF1R | 2617,5 | 3399,4 | 1518,5 | 2797,7 | 1678,3 | 7108,8 | 4304,5 | 3358,1 | 3555,1 | 2250,5 | 2619,9 | 4998,5 | 10224,6 | 1131,9 | 1453,9 | 4336,7 | 6657,9 | 3047,4 |
| P05109 | Protein S100-A8 | S10A8 | S100A8 | 19230,8 | 10648,1 | 18773,3 | 6747,0 | 10086,8 | 9259,1 | 12415,4 | 6836,2 | 13162,3 | 9021,8 | 7212,6 | 9482,7 | 4479,0 | 15644,8 | 13779,8 | 14196,5 | 11590,2 | 14170,7 |
| Q15113 | Procollagen C-endopeptidase enhancer 1 | PCOC1 | PCOLCE | 15924,8 | 28281,4 | 92066,7 | 37962,1 | 18825,0 | 14078,9 | 41815,9 | 20275,3 | 10971,9 | 15297,8 | 16962,1 | 17160,2 | 23520,1 | 28661,3 | 11649,3 | 30445,2 | 11436,5 | 21093,8 |
| Q9UNW1 | Multiple inositol polyphosphate phosphatase 1 | MINP1 | MINPP1 | 16951,3 | 16745,0 | 31367,2 | 17798,4 | 17057,5 | 23439,2 | 58020,2 | 140265,3 | 6162,4 | 15365,3 | 6009,1 | 29011,4 | 46670,9 | 40591,8 | 130528,8 | 26130,7 | 15562,1 | 8514,5 |
| P63104 | 14-3-3 protein zeta/delta | 1433Z | YWHAZ | 131513,7 | 2250,2 | 147581,4 | 77366,4 | 5452,0 | 4724,6 | 300926,7 | 156525,0 | 52815,2 | 95405,1 | 97872,4 | 269150,9 | 24377,8 | 57307,7 | 64812,2 | 160555,2 | 213323,5 | 184110,3 |
| P00739 | Haptoglobin-related protein | HPTR | HPR | 2509,5 | 3397,7 | 5253,0 | 2142,1 | 2541,2 | 2530,0 | 1929,1 | 1798,4 | 3551,3 | 2090,3 | 549,0 | 2183,1 | 2911,6 | 2255,7 | 3243,2 | 1598,0 | 5252,4 | 3499,6 |
| P12273 | Prolactin-inducible protein | PIP | PIP | 25557,7 | 21477,2 | 26006,7 | 83069,6 | 14129,9 | 42819,8 | 68714,6 | 33057,6 | 34513,4 | 53285,4 | 8260,7 | 22717,6 | 23063,0 | 62362,7 | 24346,3 | 28665,0 | 13135,2 | 33699,0 |
| Q92820 | Gamma-glutamyl hydrolase | GGH | GGH | 8067,8 | 7601,7 | 4636,0 | 3631,6 | 7906,3 | 6545,1 | 6989,8 | 5422,4 | 9454,0 | 12834,2 | 14737,2 | 3655,6 | 2713,5 | 2606,6 | 11842,0 | 10071,2 | 9335,3 | 35921,9 |
| P01034 | Cystatin-C | CYTC | CST3 | 28741,2 | 51010,7 | 25195,1 | 21034,2 | 18230,5 | 19648,8 | 41626,5 | 28025,7 | 45057,6 | 35211,0 | 21908,8 | 27725,1 | 49514,0 | 51229,1 | 64010,0 | 35755,4 | 42001,8 | 14621,9 |
| P0DJI8 | Serum amyloid A-1 protein | SAA1 | SAA1 | 12304,6 | 16898,2 | 37613,0 | 21353,4 | 11443,7 | 16817,4 | 25903,2 | 12760,3 | 6924,0 | 14306,6 | 18757,6 | 23834,9 | 13698,3 | 13068,0 | 59481,6 | 12424,6 | 17957,1 | 25763,9 |
| P04406 | Glyceraldehyde-3-phosphate dehydrogenase | G3P | GAPDH | 32343,7 | 27811,7 | 17026,6 | 9220,1 | 28489,3 | 17564,1 | 13274,1 | 32425,7 | 16365,0 | 49483,7 | 11777,9 | 34373,7 | 18540,5 | 23660,5 | 23277,2 | 8999,5 | 31153,3 | 7159,0 |
| P04259 | Keratin, type II cytoskeletal 6B | K2C6B | KRT6B | 135625,1 | 26185,7 | 14992,4 | 74785,9 | 6892,3 | 15500,7 | 80675,9 | 7178,5 | 8538,3 | 35278,7 | 14202,0 | 23645,9 | 39517,1 | 26440,6 | 67752,4 | 18912,8 | 26627,8 | 20923,8 |
| P02776 | Platelet factor 4 | PLF4 | PF4 | 767,5 | 1506,0 | 1529,7 | 408,2 | 1382,6 | 4379,4 | 1318,5 | 2895,7 | 549,4 | 921,4 | 1347,8 | 1414,5 | 2904,0 | 4056,5 | 4898,4 | 6238,7 | 5429,1 | 2301,4 |
| Q7Z7G0 | Target of Nesh-SH3 | TARSH | ABI3BP | 24513,4 | 25864,4 | 24521,7 | 25406,1 | 22462,3 | 4746,3 | 8072,1 | 11823,9 | 13532,2 | 11956,4 | 17478,3 | 12356,2 | 17679,8 | 10802,0 | 20852,7 | 35047,5 | 10115,5 | 28592,7 |
| Q14126 | Desmoglein-2 | DSG2 | DSG2 | 2587,2 | 25710,0 | 3912,6 | 6110,4 | 15540,4 | 15409,2 | 14315,4 | 19313,8 | 11661,0 | 10989,8 | 6425,5 | 68400,7 | 28760,1 | 7193,5 | 3815,6 | 20990,8 | 19522,9 | 3683,4 |
| Q13103 | Secreted phosphoprotein 24 | SPP24 | SPP2 | 28561,7 | 49259,8 | 50348,8 | 43482,6 | 41907,0 | 86961,6 | 49619,2 | 36998,5 | 27947,4 | 54428,6 | 52501,2 | 22647,5 | 39307,0 | 51830,0 | 20314,0 | 38636,4 | 38126,2 | 66273,2 |
| Q9H8L6 | Multimerin-2 | MMRN2 | MMRN2 | 31541,4 | 26193,5 | 312800,8 | 37026,6 | 33922,0 | 43495,7 | 66546,4 | 39965,4 | 36430,9 | 44037,2 | 24970,9 | 304088,2 | 36617,8 | 41418,1 | 26340,5 | 28153,8 | 41953,6 | 46101,6 |
| P14543 | Nidogen-1 | NID1 | NID1 | 883,6 | 2666,5 | 9277,6 | 2434,2 | 5224,3 | 9206,5 | 5997,9 | 1122,1 | 1806,6 | 954,5 | 5865,7 | 590,7 | 4131,9 | 1862,3 | 3983,4 | 3851,8 | 2916,0 | 3102,5 |
| P0DOX2 | Immunoglobulin alpha-2 heavy chain | IGA2 | 1 SV | 88428,7 | 261010,9 | 256400,8 | 84473,0 | 192991,7 | 403419,6 | 53529,2 | 146429,5 | 407882,8 | 70547,7 | 83625,7 | 57541,9 | 60406,0 | 79143,8 | 104708,0 | 70658,2 | 62508,5 | 82433,9 |
| P23141 | Liver carboxylesterase 1 | EST1 | CES1 | 786,5 | 2134,5 | 370,6 | 352,0 | 1007,9 | 1315,1 | 4221,1 | 637,6 | 1491,5 | 506,3 | 500,3 | 996,5 | 383,4 | 191,6 | 5051,4 | 107,9 | 2438,3 | 443,1 |
| P49913 | Cathelicidin antimicrobial peptide | CAMP | CAMP | 22171,6 | 11095,8 | 32888,4 | 14008,0 | 12117,4 | 25027,9 | 30687,3 | 6384,5 | 25950,5 | 20225,3 | 4975,1 | 3537,4 | 10197,3 | 10254,7 | 7117,6 | 13967,7 | 15159,5 | 9115,5 |
| Q8NI99 | Angiopoietin-related protein 6 | ANGL6 | ANGPTL6 | 15047,5 | 10666,8 | 20278,1 | 9863,9 | 5115,8 | 10758,9 | 15989,9 | 9402,0 | 17216,7 | 10479,0 | 2898,2 | 23153,8 | 21941,3 | 8569,2 | 11797,8 | 8922,2 | 3966,9 | 7870,6 |
| P07195 | L-lactate dehydrogenase B chain | LDHB | LDHB | 829,8 | 2471,6 | 1015,9 | 1487,0 | 2092,1 | 2523,0 | 10426,0 | 1759,2 | 1253,0 | 5340,9 | 2535,8 | 2685,2 | 2497,7 | 2834,0 | 3028,3 | 13368,5 | 1726,5 | 6016,1 |
| Q86U17 | Serpin A11 | SPA11 | SERPINA11 | 13464,7 | 22400,2 | 23692,8 | 3460,3 | 7878,1 | 21100,5 | 14467,2 | 4946,5 | 10440,4 | 16880,6 | 8431,1 | 8361,1 | 16472,1 | 13492,6 | 9100,4 | 14531,4 | 13198,1 | 52123,5 |
| P08253 | 72 kDa type IV collagenase | MMP2 | MMP2 | 6961,9 | 6681,9 | 1453,1 | 4013,8 | 8690,2 | 171,3 | 359,1 | 12884,0 | 293,8 | 510,9 | 1118,8 | 1116,7 | 10859,6 | 2039,7 | 1951,4 | 4308,3 | 3471,1 | 11502,8 |
| P04745 | Alpha-amylase 1 | AMY1 | AMY1A | 50230,1 | 25948,6 | 44310,2 | 52527,6 | 55338,2 | 74740,8 | 29496,4 | 56660,9 | 83166,3 | 54898,4 | 32545,6 | 50195,5 | 34792,4 | 49478,0 | 54300,1 | 41161,9 | 56219,5 | 70614,5 |
| P18065 | Insulin-like growth factor-binding protein 2 | IBP2 | IGFBP2 | 2345,1 | 12441,9 | 9097,8 | 12303,3 | 17861,7 | 9545,8 | 38217,9 | 10654,2 | 11822,6 | 6811,2 | 5910,3 | 8999,5 | 4621,7 | 8535,2 | 2818,4 | 8656,9 | 12108,1 | 10343,0 |
| P01860 | Immunoglobulin heavy constant gamma 3 | IGHG3 | IGHG3 | 4643395,1 | 3885542,0 | 4679148,4 | 5312884,5 | 5469498,9 | 7612076,3 | 3627163,5 | 3738651,7 | 6657849,6 | 4636043,5 | 2673567,7 | 3770337,2 | 3412273,0 | 6059886,7 | 7277455,2 | 5998816,5 | 5624945,1 | 4442720,8 |
| P09467 | Fructose-1,6-bisphosphatase 1 | F16P1 | FBP1 | 1145,0 | 1530,2 | 2940,3 | 1334,8 | 3881,3 | 624,4 | 371,8 | 375,1 | 1678,8 | 2214,3 | 387,2 | 2704,8 | 1459,5 | 707,9 | 5509,6 | 947,7 | 8823,3 | 4468,8 |
| P08294 | Extracellular superoxide dismutase [Cu-Zn] | SODE | SOD3 | 26525,9 | 14976,8 | 34000,6 | 9472,0 | 10186,6 | 9923,6 | 10720,6 | 24373,8 | 9054,5 | 24997,0 | 7640,5 | 26123,4 | 24992,4 | 5843,8 | 7510,5 | 9635,4 | 26332,3 | 25637,2 |
| P01591 | Immunoglobulin J chain | IGJ | JCHAIN | 1012,0 | 230,5 | 248,3 | 227,2 | 796,6 | 436,1 | 560,7 | 350,9 | 407,2 | 348,2 | 400,5 | 533,3 | 568,8 | 492,0 | 72,8 | 78,1 | 330,0 | 125,7 |
| P07737 | Profilin-1 | PROF1 | PFN1 | 15379,1 | 24046,2 | 57228,2 | 38515,4 | 35631,8 | 34813,6 | 55773,4 | 8950,3 | 18802,4 | 11972,6 | 10444,5 | 75516,4 | 18655,2 | 28476,4 | 18726,4 | 20868,6 | 25180,8 | 15256,2 |
| P07339 | Cathepsin D | CATD | CTSD | 28851,6 | 34606,5 | 22853,8 | 36542,1 | 7822,9 | 48341,1 | 46464,2 | 36081,1 | 21507,5 | 34200,8 | 28878,5 | 13165,6 | 43831,5 | 62099,2 | 31447,2 | 9363,5 | 17613,6 | 27575,7 |
| P78417 | Glutathione S-transferase omega-1 | GSTO1 | GSTO1 | 10090,6 | 6882,1 | 10412,6 | 23155,6 | 27954,6 | 12915,1 | 21852,1 | 25294,1 | 12878,5 | 18044,2 | 8689,7 | 16971,7 | 18673,4 | 12324,3 | 11515,0 | 26037,8 | 8762,6 | 12011,3 |
| P21333 | Filamin-A | FLNA | FLNA | 10340,4 | 11398,2 | 8771,7 | 27104,8 | 11678,6 | 16328,8 | 46085,9 | 14310,1 | 15944,5 | 3464,0 | 7740,1 | 13060,8 | 21010,4 | 20604,4 | 10821,2 | 12122,0 | 20736,2 | 20098,0 |
| P03950 | Angiogenin | ANGI | ANG | 14257,2 | 1460,5 | 7734,2 | 2424,6 | 24678,9 | 26115,4 | 4662,0 | 3406,4 | 16087,0 | 20965,0 | 4561,7 | 4132,8 | 2072,4 | 2652,1 | 21873,4 | 18112,7 | 1484,2 | 8411,2 |
| P34096 | Ribonuclease 4 | RNAS4 | RNASE4 | 1734,8 | 53055,5 | 27080,6 | 8032,3 | 59087,5 | 65203,4 | 35656,6 | 9700,6 | 5869,9 | 38871,7 | 4178,0 | 14217,9 | 47615,7 | 9304,0 | 33880,6 | 5580,3 | 13750,8 | 10566,5 |
| Q13740 | CD166 antigen | CD166 | ALCAM | 21030,3 | 21672,2 | 8862,7 | 25036,2 | 60234,2 | 13835,4 | 29817,5 | 6749,7 | 12793,5 | 31315,8 | 28160,8 | 11423,6 | 39855,4 | 10191,9 | 58812,8 | 28095,9 | 30523,7 | 98931,1 |
| P55056 | Apolipoprotein C-IV | APOC4 | APOC4 | 35462,0 | 16235,8 | 14465,5 | 15307,8 | 12907,1 | 16811,4 | 3635,0 | 15620,0 | 22126,6 | 17578,8 | 31478,7 | 4649,9 | 12269,7 | 1592,3 | 24151,5 | 13219,7 | 11607,4 | 9543,3 |
| P08319 | Alcohol dehydrogenase 4 | ADH4 | ADH4 | 3245,6 | 2474,3 | 1711,2 | 9849,7 | 1255,9 | 10633,6 | 2545,0 | 9823,1 | 4659,7 | 3465,8 | 12175,0 | 3013,0 | 7396,4 | 5805,9 | 5701,8 | 4378,9 | 6916,4 | 4983,8 |
| Q08554 | Desmocollin-1 | DSC1 | DSC1 | 30306,0 | 58406,8 | 26476,6 | 33791,1 | 14888,7 | 23355,9 | 61574,1 | 85643,7 | 33480,6 | 22498,6 | 16483,9 | 50079,9 | 38001,6 | 15935,8 | 12763,4 | 16508,7 | 26018,6 | 12633,8 |
| P16930 | Fumarylacetoacetase | FAAA | FAH | 5551,8 | 4811,2 | 6130,9 | 1196,4 | 3864,4 | 5136,8 | 1116,5 | 4619,6 | 8041,1 | 3198,5 | 1241,0 | 6651,3 | 3520,9 | 3357,8 | 4280,1 | 429,5 | 11293,1 | 4334,4 |
| P15151 | Poliovirus receptor | PVR | PVR | 3700,1 | 5409,5 | 2670,2 | 7456,5 | 6138,4 | 13918,4 | 3894,4 | 5281,9 | 7731,0 | 7885,5 | 7275,1 | 380,7 | 4006,6 | 3150,0 | 3120,2 | 1087,3 | 420,8 | 4972,5 |
| Q14515 | SPARC-like protein 1 | SPRL1 | SPARCL1 | 9624,4 | 4611,1 | 4044,0 | 10156,5 | 3309,7 | 6458,4 | 6044,2 | 3965,8 | 4567,4 | 11057,6 | 3370,5 | 19778,2 | 8547,9 | 2937,6 | 4737,6 | 12078,5 | 9638,1 | 8876,2 |
| P00352 | Retinal dehydrogenase 1 | AL1A1 | ALDH1A1 | 23591,3 | 29178,0 | 71363,1 | 8563,4 | 16356,2 | 22291,5 | 22218,3 | 14197,1 | 16617,6 | 7305,5 | 17722,0 | 33098,8 | 20422,5 | 6433,5 | 10134,5 | 7653,7 | 6111,8 | 18389,5 |
| P05451 | Lithostathine-1-alpha | REG1A | REG1A | 2731,1 | 3209,5 | 20161,9 | 7560,3 | 9226,1 | 7865,1 | 2364,6 | 11173,2 | 3438,1 | 2128,1 | 3042,7 | 3203,2 | 997,9 | 1599,0 | 10033,6 | 5934,5 | 6748,2 | 10392,8 |
| P16035 | Metalloproteinase inhibitor 2 | TIMP2 | TIMP2 | 1380,0 | 3516,1 | 11743,2 | 3470,1 | 20723,8 | 1638,1 | 633,4 | 29219,5 | 3628,4 | 15323,3 | 1841,0 | 8015,7 | 5025,9 | 4732,7 | 4868,3 | 13907,4 | 3979,0 | 4160,0 |
| Q01459 | Di-N-acetylchitobiase | DIAC | CTBS | 10485,9 | 14146,4 | 8575,1 | 16122,9 | 7015,0 | 9318,1 | 4725,7 | 6042,2 | 6390,2 | 5433,6 | 10811,0 | 7657,6 | 9849,9 | 6213,4 | 6277,4 | 7673,9 | 6381,7 | 6143,2 |
| P06733 | Alpha-enolase | ENOA | ENO1 | 15391,7 | 56487,9 | 15063,4 | 8134,5 | 19457,8 | 9984,9 | 36430,3 | 12426,5 | 18122,3 | 12982,8 | 15985,3 | 22249,6 | 51595,0 | 13183,6 | 4204,9 | 13575,9 | 5404,0 | 18592,0 |
| A0A0C4DH38 | Immunoglobulin heavy variable 5-51 | HV551 | IGHV5-51 | 2586,6 | 6594,8 | 3783,4 | 11278,9 | 15489,8 | 6286,9 | 2117,0 | 3728,5 | 7638,2 | 2548,6 | 8909,1 | 1199,8 | 737,1 | 11779,3 | 2180,0 | 10237,3 | 1972,4 | 2190,2 |
| P30046 | D-dopachrome decarboxylase | DOPD | DDT | 7891,6 | 10528,4 | 41362,3 | 4090,1 | 4753,9 | 8996,2 | 14236,7 | 6561,5 | 3162,8 | 15739,3 | 4647,2 | 30558,7 | 10543,7 | 2587,9 | 3791,3 | 29439,0 | 8486,2 | 6956,4 |
| P62805 | Histone H4 | H4 | HIST1H4A | 153492,5 | 161357,4 | 128283,8 | 180610,2 | 189959,7 | 226566,2 | 158649,2 | 176721,4 | 157398,0 | 147686,4 | 154719,3 | 90881,7 | 136341,8 | 166148,1 | 155252,5 | 210106,9 | 161888,9 | 186253,0 |
| P04083 | Annexin A1 | ANXA1 | ANXA1 | 13085,9 | 6598,6 | 3865,1 | 7634,6 | 6628,1 | 3182,4 | 14285,6 | 5835,7 | 4338,6 | 5081,0 | 8661,5 | 11812,2 | 13143,0 | 4773,6 | 2304,5 | 10809,1 | 7535,9 | 3418,7 |
| P39059 | Collagen alpha-1(XV) chain | COFA1 | COL15A1 | 7147,6 | 1252,7 | 14461,0 | 18475,1 | 6649,7 | 3222,7 | 3690,8 | 3927,3 | 3618,2 | 12102,4 | 6656,3 | 1520,6 | 2680,3 | 16898,9 | 7804,2 | 4347,8 | 10729,0 | 5986,1 |
| P14618 | Pyruvate kinase PKM | KPYM | PKM | 10279,1 | 25712,7 | 25022,9 | 54834,7 | 85798,6 | 52718,1 | 31090,4 | 10759,4 | 33396,1 | 11685,9 | 92394,8 | 14852,3 | 46947,3 | 48190,3 | 29874,5 | 12659,4 | 19143,0 | 12584,6 |
| P01599 | Immunoglobulin kappa variable 1-17 | KV117 | IGKV1-17 | 151,5 | 113,3 | 316,6 | 201,3 | 586,9 | 100,1 | 361,9 | 864,6 | 569,6 | 130,3 | 159,0 | 1263,7 | 1256,0 | 343,8 | 87,7 | 287,3 | 427,4 | 265,0 |
| P12035 | Keratin, type II cytoskeletal 3 | K2C3 | KRT3 | 36418,7 | 11815,9 | 19252,6 | 52385,2 | 37798,3 | 72448,3 | 28135,4 | 36764,3 | 64006,8 | 40794,3 | 30435,0 | 14699,4 | 39720,9 | 52904,7 | 42432,5 | 29448,6 | 32875,6 | 26477,8 |
| P02654 | Apolipoprotein C-I | APOC1 | APOC1 | 204681,6 | 164478,7 | 75025,5 | 147815,4 | 130876,9 | 122595,2 | 89342,7 | 144125,0 | 177981,6 | 218544,4 | 129111,9 | 15015,5 | 230449,1 | 125225,5 | 190075,2 | 151265,1 | 49472,0 | 106551,6 |
| P02775 | Platelet basic protein | CXCL7 | PPBP | 5285,7 | 7030,7 | 10786,2 | 4994,9 | 8252,5 | 3739,4 | 19659,2 | 9603,3 | 9240,8 | 3217,2 | 3388,5 | 9654,8 | 8918,1 | 4633,4 | 11523,7 | 3735,3 | 4677,6 | 2872,1 |
| P01861 | Immunoglobulin heavy constant gamma 4 | IGHG4 | IGHG4 | 1464,2 | 1585,4 | 1205,6 | 3478,4 | 3152,8 | 3429,1 | 1043,0 | 1526,1 | 1432,0 | 855,0 | 738,9 | 917,4 | 1779,9 | 1018,8 | 1440,4 | 4762,0 | 1958,0 | 3457,6 |
| Q7Z3Y8 | Keratin, type I cytoskeletal 27 | K1C27 | KRT27 | 28129,7 | 17690,4 | 17318,5 | 64899,1 | 12753,7 | 28606,8 | 33892,5 | 23189,0 | 37795,3 | 38375,9 | 25770,9 | 13623,4 | 7284,7 | 51832,8 | 14106,7 | 7780,3 | 9525,0 | 34405,9 |
| Q15848 | Adiponectin | ADIPO | ADIPOQ | 25083,0 | 31117,7 | 18806,1 | 48591,4 | 34418,7 | 12052,8 | 23187,8 | 40126,1 | 11826,8 | 14916,9 | 18472,2 | 54741,2 | 26669,9 | 38616,0 | 35801,3 | 46198,8 | 53839,5 | 18286,3 |
| Q93088 | Betaine--homocysteine S-methyltransferase 1 | BHMT1 | BHMT | 3103,1 | 8814,1 | 6716,7 | 5163,8 | 5477,1 | 5190,8 | 3989,6 | 83790,1 | 638,7 | 22903,9 | 2755,8 | 3623,7 | 1603,6 | 595,2 | 6269,4 | 4404,5 | 31903,7 | 4878,0 |
| P23470 | Receptor-type tyrosine-protein phosphatase gamma | PTPRG | PTPRG | 37291,6 | 62354,5 | 9524,4 | 16938,3 | 44080,6 | 18326,7 | 95891,7 | 32192,7 | 10590,6 | 27542,9 | 20344,5 | 11222,1 | 26236,6 | 20652,2 | 18698,1 | 34486,8 | 15528,5 | 39024,9 |
| A0A075B6K4 | Immunoglobulin lambda variable 3-10 | LV310 | IGLV3-10 | 609,7 | 648,9 | 692,3 | 901,1 | 731,2 | 743,2 | 929,9 | 611,9 | 791,9 | 478,3 | 203,1 | 1258,2 | 1131,7 | 320,9 | 440,2 | 1189,9 | 454,9 | 1031,2 |
| O15394 | Neural cell adhesion molecule 2 | NCAM2 | NCAM2 | 165258,9 | 181353,5 | 424512,0 | 139336,4 | 136117,1 | 177942,3 | 272925,5 | 553487,7 | 169314,0 | 637685,5 | 22144,5 | 817034,3 | 191489,4 | 19973,0 | 19958,8 | 14139,0 | 307065,2 | 192232,9 |
| P11717 | Cation-independent mannose-6-phosphate receptor | MPRI | IGF2R | 1843917,2 | 3435330,0 | 854793,2 | 2542919,0 | 2678756,2 | 3056096,0 | 1692956,2 | 2210459,2 | 2704292,8 | 1827794,0 | 1727422,7 | 32242,3 | 1754977,5 | 1625898,1 | 2595337,9 | 1854831,9 | 438755,4 | 1396657,5 |
| Q6KB66 | Keratin, type II cytoskeletal 80 | K2C80 | KRT80 | 5646,0 | 3058,8 | 11116,5 | 3060,3 | 3791,2 | 5921,6 | 10455,0 | 3878,8 | 6211,9 | 8722,9 | 2378,8 | 5012,4 | 10638,7 | 16292,2 | 13642,3 | 10886,9 | 10180,9 | 11893,0 |
| P62937 | Peptidyl-prolyl cis-trans isomerase A | PPIA | PPIA | 4095,5 | 5490,9 | 4417,9 | 5333,8 | 2292,0 | 4539,9 | 6178,4 | 4612,3 | 10403,5 | 3061,5 | 3554,4 | 7857,7 | 3288,2 | 2792,8 | 9944,9 | 11694,2 | 3191,0 | 3241,2 |
| Q08188 | Protein-glutamine gamma-glutamyltransferase E | TGM3 | TGM3 | 1920,5 | 22732,9 | 19590,7 | 27401,8 | 18858,5 | 19765,7 | 28088,6 | 16384,4 | 27959,2 | 20668,3 | 6264,2 | 15081,8 | 24174,0 | 9751,7 | 13127,9 | 6223,1 | 3105,2 | 11216,7 |
| P11597 | Cholesteryl ester transfer protein | CETP | CETP | 53521,9 | 47072,8 | 31523,8 | 41014,1 | 39282,7 | 26350,5 | 22614,8 | 35354,9 | 28082,4 | 34300,7 | 31775,2 | 44256,9 | 41309,2 | 22653,6 | 42001,8 | 58398,9 | 16018,4 | 58948,5 |
| Q01469 | Fatty acid-binding protein, epidermal | FABP5 | FABP5 | 2144,7 | 9605,3 | 5052,7 | 13960,9 | 2141,0 | 2336,7 | 2584,7 | 4304,9 | 18465,0 | 2445,9 | 2091,0 | 7334,7 | 13646,1 | 2914,2 | 3218,2 | 14774,2 | 914,1 | 3175,6 |
| O14818 | Proteasome subunit alpha type-7 | PSA7 | PSMA7 | 5887,7 | 3988,9 | 6393,7 | 11002,2 | 7981,1 | 13389,5 | 7810,0 | 13985,9 | 2883,1 | 8311,3 | 3565,2 | 3958,4 | 11746,5 | 1261,4 | 6408,8 | 3858,9 | 2597,1 | 279,8 |
| Q03591 | Complement factor H-related protein 1 | FHR1 | CFHR1 | 54704,2 | 33711,9 | 109041,6 | 70563,4 | 58097,9 | 105232,4 | 48437,4 | 52119,4 | 82737,3 | 1017,5 | 45794,7 | 4562,0 | 8935,4 | 60906,8 | 2231,9 | 10465,5 | 64906,5 | 7762,0 |
| P41222 | Prostaglandin-H2 D-isomerase | PTGDS | PTGDS | 31129,6 | 29157,8 | 64666,2 | 47401,7 | 21718,2 | 51642,9 | 63787,5 | 59892,9 | 23887,1 | 8334,0 | 24120,3 | 47922,6 | 35324,6 | 35371,6 | 32054,0 | 26484,1 | 89750,1 | 14428,0 |
| P31944 | Caspase-14 | CASPE | CASP14 | 25299,8 | 24355,7 | 23365,8 | 23680,2 | 18117,2 | 25077,1 | 5102,8 | 10558,3 | 5381,0 | 19848,6 | 15050,2 | 5754,0 | 11862,3 | 16926,2 | 18897,0 | 13175,9 | 18774,7 | 17156,7 |
| P60900 | Proteasome subunit alpha type-6 | PSA6 | PSMA6 | 24287,3 | 21664,2 | 115578,0 | 3653,6 | 24794,0 | 43528,2 | 31644,2 | 25422,0 | 22103,0 | 78213,3 | 14305,1 | 5845,5 | 22378,4 | 14958,2 | 67523,4 | 3653,6 | 28448,6 | 29140,4 |
| P02786 | Transferrin receptor protein 1 | TFR1 | TFRC | 1086,3 | 1356,2 | 642,3 | 756,7 | 637,8 | 1386,9 | 950,3 | 211,3 | 134,4 | 656,7 | 795,4 | 347,8 | 677,4 | 1168,7 | 607,3 | 471,4 | 1039,7 | 117,2 |
| P80748 | Immunoglobulin lambda variable 3-21 | LV321 | IGLV3-21 | 841,0 | 238,1 | 566,8 | 767,7 | 393,1 | 581,5 | 524,5 | 692,4 | 208,6 | 247,0 | 140,3 | 1555,0 | 655,2 | 372,8 | 652,2 | 492,1 | 557,0 | 935,9 |
| P02792 | Ferritin light chain | FRIL | FTL | 11742,9 | 2398,4 | 2645,8 | 12972,6 | 11338,3 | 5445,2 | 12743,4 | 2251,1 | 4405,1 | 4321,5 | 2523,8 | 15356,9 | 20957,8 | 5582,0 | 3788,1 | 3490,1 | 5223,4 | 5611,1 |
| P01033 | Metalloproteinase inhibitor 1 | TIMP1 | TIMP1 | 15260,9 | 19442,3 | 28509,3 | 16011,3 | 40749,7 | 10057,5 | 12181,2 | 6973,3 | 11708,4 | 4397,4 | 32297,5 | 11066,5 | 22530,4 | 22045,5 | 15910,6 | 13059,8 | 10995,0 | 8388,0 |
| P59666 | Neutrophil defensin 3 | DEF3 | DEFA3 | 16247,9 | 9554,2 | 3559,2 | 316737,5 | 362651,3 | 384654,0 | 2760,6 | 9007,3 | 19363,9 | 3881,4 | 5709,7 | 11453,8 | 214124,9 | 411798,4 | 182540,1 | 29223,2 | 76263,4 | 7860,6 |
| P07900 | Heat shock protein HSP 90-alpha | HS90A | HSP90AA1 | 859,1 | 2006,1 | 587,3 | 931,3 | 1672,6 | 918,8 | 1794,1 | 1401,8 | 638,2 | 1215,5 | 957,5 | 1135,9 | 302,5 | 947,4 | 1230,5 | 707,4 | 857,6 | 1441,0 |
| P81605 | Dermcidin | DCD | DCD | 25857,7 | 57891,5 | 36400,9 | 27109,3 | 24080,4 | 24081,1 | 59953,8 | 19778,9 | 14795,4 | 18580,4 | 20294,2 | 25170,0 | 17546,9 | 13855,5 | 15992,5 | 23910,3 | 26408,3 | 28730,8 |
| P01701 | Immunoglobulin lambda variable 1-51 | LV151 | IGLV1-51 | 1277,8 | 1959,4 | 617,6 | 506,1 | 1218,5 | 2122,2 | 2160,6 | 1129,5 | 202,1 | 1054,3 | 2436,1 | 876,5 | 1131,1 | 585,8 | 1162,3 | 1476,4 | 1046,0 | 311,6 |
| P06312 | Immunoglobulin kappa variable 4-1 | KV401 | IGKV4-1 | 3082,4 | 2733,3 | 3898,0 | 1239,8 | 3415,2 | 3719,4 | 30709,7 | 7951,5 | 2016,8 | 2701,0 | 956,5 | 6481,2 | 3296,3 | 2543,1 | 1794,8 | 3219,9 | 1418,6 | 3759,9 |
| P32754 | 4-hydroxyphenylpyruvate dioxygenase | HPPD | HPD | 26467,5 | 16442,0 | 14524,7 | 14907,2 | 15811,1 | 11075,2 | 21893,3 | 7250,9 | 6722,1 | 12952,2 | 7920,6 | 17039,0 | 7752,5 | 4823,0 | 11099,6 | 22559,3 | 18998,9 | 20316,5 |
| P04433 | Immunoglobulin kappa variable 3-11 | KV311 | IGKV3-11 | 5323,6 | 150,9 | 1053,3 | 950,6 | 2773,4 | 784,7 | 553,1 | 2881,3 | 2395,3 | 851,2 | 568,7 | 2144,2 | 2270,8 | 1581,7 | 294,4 | 506,9 | 2065,1 | 5345,2 |
| Q08257 | Quinone oxidoreductase | QOR | CRYZ | 4508,1 | 2190,0 | 12553,8 | 6314,6 | 3233,9 | 6717,2 | 5291,3 | 5324,2 | 3756,0 | 1616,0 | 5668,4 | 4131,3 | 2675,8 | 3648,0 | 3732,2 | 1540,6 | 2969,3 | 2697,8 |
| P52758 | 2-iminobutanoate/2-iminopropanoate deaminase | RIDA | RIDA | 5734,9 | 2456,8 | 9031,5 | 6342,5 | 841,4 | 1104,5 | 2590,3 | 4792,9 | 812,7 | 10808,4 | 4286,0 | 2404,8 | 5250,7 | 1797,4 | 1424,3 | 2949,1 | 8904,2 | 2789,5 |
| P06331 | Immunoglobulin heavy variable 4-34 | HV434 | IGHV4-34 | 25088,7 | 24260,3 | 8642,5 | 18152,7 | 7213,2 | 23029,0 | 2286,7 | 24178,7 | 7883,9 | 7762,4 | 5624,0 | 6182,7 | 8328,3 | 2718,7 | 22151,4 | 18465,9 | 5313,6 | 5874,3 |
| P01703 | Immunoglobulin lambda variable 1-40 | LV140 | IGLV1-40 | 897,5 | 2092,3 | 1134,1 | 696,9 | 1570,7 | 1123,1 | 1378,9 | 1389,3 | 3451,7 | 1096,8 | 897,3 | 1086,0 | 1273,6 | 615,0 | 540,9 | 400,5 | 1986,2 | 3183,1 |
| A0A0C4DH68 | Immunoglobulin kappa variable 2-24 | KV224 | IGKV2-24 | 2670,4 | 2166,7 | 2098,8 | 1483,6 | 2137,3 | 1510,5 | 778,0 | 3692,1 | 1493,3 | 3269,9 | 3478,4 | 1031,6 | 3272,0 | 2097,3 | 2588,7 | 1325,6 | 1736,3 | 1274,8 |
| P01700 | Immunoglobulin lambda variable 1-47 | LV147 | IGLV1-47 | 2027,5 | 159,0 | 906,6 | 5134,4 | 5317,7 | 239,4 | 1358,4 | 469,3 | 2805,1 | 641,4 | 3084,6 | 593,5 | 6412,9 | 719,1 | 6150,6 | 1704,6 | 3594,5 | 2175,3 |
| P01833 | Polymeric immunoglobulin receptor | PIGR | PIGR | 321,1 | 420,8 | 370,5 | 427,8 | 327,4 | 326,0 | 1730,2 | 293,9 | 366,1 | 1144,6 | 299,7 | 2889,5 | 2015,4 | 517,7 | 692,5 | 133,1 | 1252,3 | 637,6 |
| Q9NPY3 | Complement component C1q receptor | C1QR1 | CD93 | 22425,8 | 6119,3 | 22501,6 | 9013,1 | 13370,9 | 18989,6 | 6058,6 | 8023,4 | 18361,5 | 11852,9 | 21419,9 | 8427,4 | 3775,3 | 10971,7 | 15517,9 | 20918,1 | 16715,3 | 11942,7 |
| P22897 | Macrophage mannose receptor 1 | MRC1 | MRC1 | 34114,4 | 5650,4 | 5262,9 | 13495,6 | 9669,9 | 12755,4 | 1840,8 | 5198,3 | 12889,2 | 6018,1 | 4269,0 | 4265,2 | 1237,4 | 13163,9 | 3730,5 | 17825,4 | 7665,2 | 1615,4 |
| O75015 | Low affinity immunoglobulin gamma Fc region receptor III-B | FCG3B | FCGR3B | 773,4 | 24079,2 | 20463,0 | 949,0 | 2311,3 | 1112,0 | 1211,2 | 179,4 | 11521,0 | 2433,9 | 2976,8 | 7561,6 | 2035,5 | 3358,6 | 562,1 | 1619,0 | 15216,5 | 975,2 |
| P22735 | Protein-glutamine gamma-glutamyltransferase K | TGM1 | TGM1 | 1147,8 | 396,9 | 350,5 | 132,0 | 621,1 | 425,5 | 423,6 | 241,6 | 645,6 | 219,8 | 172,5 | 598,2 | 469,8 | 1250,9 | 89,2 | 895,6 | 805,9 | 352,1 |
| P29508 | Serpin B3 | SPB3 | SERPINB3 | 12604,1 | 13368,7 | 10137,6 | 25307,3 | 25100,3 | 14822,4 | 9139,5 | 25990,2 | 24137,0 | 16412,3 | 28393,4 | 15531,4 | 16166,7 | 21308,2 | 15212,6 | 16545,7 | 25503,7 | 16120,0 |
| P04424 | Argininosuccinate lyase | ARLY | ASL | 4671,0 | 2366,3 | 6263,9 | 1362,2 | 3837,1 | 1488,6 | 1741,6 | 2909,6 | 3996,5 | 1821,9 | 2879,6 | 2797,5 | 4897,5 | 1000,7 | 1057,4 | 957,3 | 2910,6 | 1486,8 |
| Q9H4G4 | Golgi-associated plant pathogenesis-related protein 1 | GAPR1 | GLIPR2 | 1309,9 | 8663,2 | 1965,1 | 2728,6 | 1942,0 | 6843,1 | 2949,5 | 3529,0 | 3602,5 | 4411,7 | 1613,6 | 7315,2 | 3888,6 | 1264,7 | 1954,1 | 2824,4 | 1923,7 | 1890,8 |
| P54289 | Voltage-dependent calcium channel subunit alpha-2/delta-1 | CA2D1 | CACNA2D1 | 1410,8 | 8549,9 | 35770,2 | 5148,8 | 3497,2 | 2893,7 | 6444,3 | 4278,2 | 5173,9 | 4793,3 | 6921,8 | 22015,1 | 2120,6 | 3800,1 | 16238,9 | 3254,2 | 7766,9 | 5385,4 |
| P00338 | L-lactate dehydrogenase A chain | LDHA | LDHA | 1336,7 | 12333,3 | 5732,4 | 3024,5 | 3385,4 | 9555,1 | 5043,3 | 18220,4 | 3389,9 | 2887,8 | 4139,5 | 5656,2 | 7688,7 | 5210,1 | 6400,8 | 3028,7 | 19279,0 | 6519,5 |
| Q13835 | Plakophilin-1 | PKP1 | PKP1 | 2975,1 | 2718,2 | 12394,3 | 7568,8 | 11654,3 | 14996,2 | 17698,2 | 4056,2 | 7978,5 | 2218,5 | 3659,3 | 4576,0 | 7604,9 | 3823,3 | 3965,1 | 3710,2 | 16259,9 | 5943,7 |
| P13727 | Bone marrow proteoglycan | PRG2 | PRG2 | 44736,0 | 6326,0 | 40949,9 | 23401,1 | 29241,2 | 18174,4 | 15819,7 | 12008,1 | 16845,2 | 65091,6 | 12096,6 | 13561,6 | 23011,1 | 12915,2 | 25291,2 | 9971,0 | 11782,8 | 15945,7 |
| P10586 | Receptor-type tyrosine-protein phosphatase F | PTPRF | PTPRF | 18033,3 | 4913,5 | 4212,1 | 31791,7 | 18285,9 | 24704,3 | 3817,5 | 6166,3 | 1877,8 | 10378,6 | 2813,4 | 14043,1 | 3465,1 | 10875,8 | 4972,4 | 32136,6 | 11824,2 | 18226,5 |
| P37802 | Transgelin-2 | TAGL2 | TAGLN2 | 2330,1 | 1160,1 | 22850,8 | 7512,2 | 2208,7 | 1928,6 | 2556,4 | 5194,5 | 5948,1 | 11710,4 | 6595,1 | 10829,6 | 39498,7 | 3820,8 | 1273,5 | 8570,8 | 7095,4 | 3259,0 |
| P02042 | Hemoglobin subunit delta | HBD | HBD | 748,2 | 661,1 | 370,5 | 517,1 | 759,5 | 121,4 | 541,0 | 340,7 | 1038,9 | 607,4 | 675,1 | 730,2 | 563,7 | 1620,1 | 448,0 | 314,3 | 622,5 | 1055,8 |
| P05089 | Arginase-1 | ARGI1 | ARG1 | 44035,4 | 38618,2 | 23166,6 | 32917,2 | 53630,2 | 29717,1 | 30066,0 | 18734,2 | 25378,7 | 14736,8 | 41525,0 | 25749,2 | 64046,5 | 24774,0 | 38735,5 | 22216,7 | 22056,3 | 19557,5 |
| O75223 | Gamma-glutamylcyclotransferase | GGCT | GGCT | 56540,2 | 56908,5 | 16304,4 | 20786,4 | 111751,8 | 42872,7 | 45974,7 | 65248,0 | 38808,6 | 33935,8 | 29234,2 | 59498,3 | 86291,7 | 49504,1 | 64808,1 | 45492,6 | 32623,0 | 18148,0 |
| P78385 | Keratin, type II cuticular Hb3 | KRT83 | KRT83 | 40266,8 | 100424,0 | 44813,5 | 47946,5 | 69006,3 | 64601,4 | 64991,4 | 24259,8 | 41055,8 | 35021,4 | 35335,1 | 46222,3 | 19997,6 | 20100,8 | 59104,8 | 80063,9 | 77359,7 | 40890,0 |
| P25774 | Cathepsin S | CATS | CTSS | 1532,1 | 11792,3 | 18268,5 | 28256,4 | 15243,0 | 1595,0 | 14404,9 | 2473,9 | 17802,9 | 7268,9 | 3989,0 | 4713,0 | 2759,4 | 4735,4 | 14657,6 | 17767,7 | 1652,7 | 12027,3 |
| P01714 | Immunoglobulin lambda variable 3-19 | LV319 | IGLV3-19 | 1177,0 | 309,8 | 1081,3 | 2552,5 | 1694,3 | 5474,8 | 1309,5 | 1178,6 | 1062,9 | 874,3 | 834,1 | 2059,6 | 1127,3 | 1009,4 | 1586,6 | 1436,6 | 4404,7 | 1439,7 |
| P0DML3 | Chorionic somatomammotropin hormone 2 | CSH2 | CSH2 | 29058,6 | 31962,6 | 48923,4 | 43560,3 | 22844,7 | 23657,7 | 32053,7 | 37750,3 | 23827,2 | 16012,6 | 5100,2 | 5953,1 | 15154,5 | 13708,9 | 19430,9 | 18493,4 | 19959,2 | 23581,3 |
| P02008 | Hemoglobin subunit zeta | HBAZ | HBZ | 614,6 | 3106,7 | 4331,4 | 4902,9 | 1003,6 | 3347,5 | 7428,6 | 2253,9 | 572,6 | 1300,1 | 555,9 | 1363,8 | 5792,1 | 652,2 | 164,6 | 3040,5 | 753,7 | 2152,4 |
| Q9BWD1 | Acetyl-CoA acetyltransferase, cytosolic | THIC | ACAT2 | 18040,1 | 4453,3 | 13031,9 | 2193,7 | 3815,5 | 11821,7 | 8462,0 | 14963,3 | 2977,5 | 4954,5 | 4327,6 | 5802,4 | 6295,6 | 4550,1 | 20499,9 | 8033,7 | 4053,4 | 6504,9 |
| Q9UNN8 | Endothelial protein C receptor | EPCR | PROCR | 18135,1 | 13658,3 | 21332,1 | 14998,4 | 13443,2 | 17314,1 | 11796,0 | 12188,1 | 13996,6 | 15129,0 | 13200,6 | 25629,6 | 16356,2 | 10762,4 | 27802,8 | 7974,8 | 10287,7 | 20287,8 |
| Q8N6C8 | Leukocyte immunoglobulin-like receptor subfamily A member 3 | LIRA3 | LILRA3 | 1768,4 | 11100,5 | 2407,7 | 2901,3 | 2200,3 | 3315,9 | 7212,2 | 3509,0 | 2837,3 | 10765,5 | 2323,3 | 825,2 | 1686,6 | 1466,2 | 1969,2 | 1500,3 | 1364,2 | 3156,5 |
| P20618 | Proteasome subunit beta type-1 | PSB1 | PSMB1 | 3415,2 | 2479,4 | 34631,5 | 6047,1 | 6270,6 | 3685,4 | 2398,8 | 2585,9 | 4097,7 | 2492,7 | 9798,4 | 11640,1 | 7963,4 | 5898,1 | 5790,2 | 5732,8 | 7833,8 | 4117,2 |
| Q96P63 | Serpin B12 | SPB12 | SERPINB12 | 36940,0 | 5758,6 | 3222,6 | 1807,9 | 17468,3 | 13377,9 | 14973,8 | 6355,7 | 4875,8 | 25317,2 | 11303,6 | 5895,7 | 14893,4 | 45540,5 | 13078,5 | 26246,6 | 6629,5 | 14029,2 |
| Q6P179 | Endoplasmic reticulum aminopeptidase 2 | ERAP2 | ERAP2 | 92830,9 | 8561,9 | 8411,3 | 9288,1 | 9869,6 | 19181,0 | 7204,3 | 113311,5 | 6308,3 | 10812,5 | 650,9 | 9628,6 | 8249,7 | 32802,4 | 5172,4 | 24276,9 | 11559,2 | 24249,6 |
| Q9Y646 | Carboxypeptidase Q | CBPQ | CPQ | 299140,5 | 333599,8 | 22076,0 | 393463,6 | 388960,5 | 199838,0 | 16735,7 | 238401,2 | 43149,9 | 353610,3 | 170893,5 | 136597,8 | 259015,9 | 229021,0 | 11819,3 | 447666,2 | 137492,9 | 175552,9 |
| P04066 | Tissue alpha-L-fucosidase | FUCO | FUCA1 | 4331,5 | 14476,6 | 5114,7 | 3456,9 | 5293,6 | 20157,5 | 10090,8 | 3612,5 | 4535,8 | 13384,7 | 9086,7 | 4814,1 | 4607,3 | 12709,5 | 14957,9 | 8059,1 | 3551,8 | 7564,8 |
| P12830 | Cadherin-1 | CADH1 | CDH1 | 2553,8 | 5246,4 | 25157,2 | 2458,2 | 7291,7 | 4486,2 | 4586,4 | 8091,8 | 4815,0 | 5514,9 | 3720,6 | 7030,4 | 5105,7 | 7722,4 | 1398,2 | 3953,5 | 9680,3 | 3848,0 |
| P01624 | Immunoglobulin kappa variable 3-15 | KV315 | IGKV3-15 | 161,2 | 105,9 | 716,5 | 373,1 | 286,5 | 247,3 | 534,5 | 473,5 | 192,6 | 688,6 | 137,7 | 591,5 | 239,1 | 155,5 | 970,5 | 842,7 | 2873,7 | 177,1 |
| P00491 | Purine nucleoside phosphorylase | PNPH | PNP | 1594,8 | 25001,7 | 35844,1 | 15218,0 | 3006,1 | 6910,4 | 12180,0 | 8211,6 | 28122,3 | 5359,9 | 3968,8 | 6801,1 | 1969,8 | 1402,8 | 7276,6 | 23738,5 | 4705,7 | 4814,9 |
| Q9BWP8 | Collectin-11 | COL11 | COLEC11 | 209508,2 | 23633,9 | 200284,2 | 20798,3 | 23121,3 | 15754,9 | 176311,2 | 27944,8 | 33166,7 | 12620,8 | 149935,8 | 165788,1 | 229519,4 | 141792,8 | 10543,4 | 22664,7 | 31623,7 | 17861,6 |
| P00918 | Carbonic anhydrase 2 | CAH2 | CA2 | 1662,1 | 4929,5 | 7167,5 | 3097,3 | 4284,6 | 1752,9 | 5579,6 | 9786,7 | 3189,6 | 3861,2 | 3289,3 | 2826,1 | 26425,2 | 3384,5 | 2945,5 | 5125,3 | 1748,3 | 3284,0 |
| Q13790 | Apolipoprotein F | APOF | APOF | 46144,2 | 52513,3 | 85630,2 | 40717,8 | 83769,8 | 17497,3 | 27737,0 | 50120,7 | 41393,0 | 28790,8 | 27233,8 | 108888,7 | 42612,7 | 17724,1 | 43939,9 | 40056,9 | 54942,0 | 58565,9 |
| Q10588 | ADP-ribosyl cyclase/cyclic ADP-ribose hydrolase 2 | BST1 | BST1 | 28774,2 | 10721,7 | 12697,1 | 18409,4 | 5057,0 | 12258,2 | 16001,5 | 13945,5 | 13551,0 | 16419,9 | 17372,6 | 6120,2 | 26886,6 | 22656,6 | 13806,8 | 26548,3 | 20918,0 | 15328,4 |
| P09972 | Fructose-bisphosphate aldolase C | ALDOC | ALDOC | 793,8 | 3258,3 | 3344,0 | 2306,1 | 492,6 | 793,4 | 957,4 | 1979,6 | 3097,2 | 231,3 | 171,2 | 582,5 | 1455,9 | 1969,8 | 478,0 | 600,0 | 3504,9 | 980,3 |
| O00299 | Chloride intracellular channel protein 1 | CLIC1 | CLIC1 | 2380,3 | 1648,4 | 1841,3 | 3291,4 | 823,8 | 2592,9 | 782,6 | 544,3 | 729,0 | 4415,6 | 1170,9 | 2083,5 | 1448,8 | 980,6 | 773,5 | 2481,3 | 3717,4 | 2167,8 |
| O43505 | Beta-1,4-glucuronyltransferase 1 | B4GA1 | B4GAT1 | 4996,6 | 21088,1 | 46391,5 | 19814,3 | 39566,4 | 87081,8 | 19270,3 | 29816,2 | 98530,4 | 21298,4 | 71432,8 | 28487,3 | 48220,1 | 22886,2 | 27282,1 | 20950,2 | 26927,6 | 15685,7 |
| Q00887 | Pregnancy-specific beta-1-glycoprotein 9 | PSG9 | PSG9 | 2075,4 | 4138,7 | 13266,9 | 5277,6 | 5149,5 | 3177,1 | 1495,0 | 3306,1 | 4258,3 | 1704,6 | 2653,4 | 2124,7 | 4148,2 | 3106,1 | 819,3 | 7728,8 | 3046,1 | 1364,1 |
| A0A0B4J1V0 | Immunoglobulin heavy variable 3-15 | HV315 | IGHV3-15 | 1343,9 | 928,9 | 740,6 | 645,8 | 225,9 | 1506,9 | 171,6 | 135,3 | 1601,1 | 922,3 | 68,8 | 858,0 | 238,4 | 630,0 | 95,0 | 1625,4 | 1396,9 | 546,9 |
| P20933 | N(4)-(beta-N-acetylglucosaminyl)-L-asparaginase | ASPG | AGA | 5760,2 | 84526,4 | 1811,0 | 1892,6 | 47327,7 | 1307,2 | 12756,8 | 65598,2 | 4321,5 | 31118,0 | 3796,9 | 5630,6 | 9493,9 | 39887,6 | 69281,1 | 36898,9 | 23342,0 | 10487,3 |
| P26038 | Moesin | MOES | MSN | 10711,2 | 6471,7 | 7043,4 | 4852,2 | 7683,2 | 18894,2 | 23781,2 | 9882,7 | 10091,5 | 3615,9 | 7415,9 | 6624,7 | 12375,0 | 6147,4 | 3513,9 | 4351,2 | 21291,6 | 11277,0 |
| P12814 | Alpha-actinin-1 | ACTN1 | ACTN1 | 827,5 | 3493,9 | 4814,8 | 5971,7 | 8232,4 | 1574,9 | 918,1 | 1574,8 | 6414,0 | 1325,3 | 3534,4 | 1496,6 | 2408,9 | 1055,0 | 2108,6 | 402,8 | 12281,6 | 4609,0 |
| P61916 | NPC intracellular cholesterol transporter 2 | NPC2 | NPC2 | 4371,2 | 6889,1 | 694,0 | 2087,6 | 9666,5 | 1313,2 | 1049,1 | 3877,4 | 1820,9 | 3398,1 | 2019,3 | 1315,9 | 1121,8 | 1615,8 | 1883,7 | 4590,6 | 3611,7 | 1177,1 |
| A0A075B6H9 | Immunoglobulin lambda variable 4-69 | LV469 | IGLV4-69 | 117657,8 | 189600,7 | 176069,4 | 103773,1 | 246903,8 | 83554,1 | 110474,6 | 142974,0 | 312134,6 | 68917,1 | 1654,4 | 71732,0 | 117915,6 | 64150,6 | 93531,8 | 154015,2 | 32433,3 | 77902,6 |
| Q96DA0 | Zymogen granule protein 16 homolog B | ZG16B | ZG16B | 12056,8 | 13861,8 | 14811,3 | 13008,1 | 11666,0 | 17842,8 | 30170,4 | 9885,9 | 5606,8 | 19188,9 | 6899,5 | 19351,3 | 11352,7 | 16656,8 | 4986,8 | 12620,0 | 21707,9 | 3242,7 |
| P00558 | Phosphoglycerate kinase 1 | PGK1 | PGK1 | 5753,9 | 11519,4 | 7755,7 | 9568,1 | 7308,8 | 8505,3 | 5418,0 | 5324,5 | 2252,6 | 3047,7 | 10616,1 | 12303,0 | 5370,0 | 3837,9 | 2602,7 | 5841,8 | 8850,0 | 6215,5 |
| Q9Y279 | V-set and immunoglobulin domain-containing protein 4 | VSIG4 | VSIG4 | 5028,0 | 1371,9 | 20599,4 | 32361,7 | 1862,8 | 31555,1 | 3144,3 | 21921,6 | 2030,0 | 21059,1 | 1512,5 | 3647,8 | 2368,2 | 2158,6 | 18398,8 | 23987,2 | 19542,2 | 17528,4 |
| P04792 | Heat shock protein beta-1 | HSPB1 | HSPB1 | 15754,4 | 7470,3 | 7536,2 | 24711,5 | 22246,9 | 8774,3 | 26567,0 | 5696,3 | 14564,3 | 15528,2 | 4303,3 | 7032,1 | 10542,5 | 4132,1 | 2291,9 | 21992,6 | 15931,9 | 7473,3 |
| Q06830 | Peroxiredoxin-1 | PRDX1 | PRDX1 | 3493,4 | 6769,1 | 7368,9 | 9173,0 | 8594,7 | 2445,0 | 2492,9 | 7370,4 | 2523,3 | 3888,7 | 2633,3 | 37034,2 | 8456,0 | 11561,4 | 3267,0 | 5115,7 | 1575,4 | 10053,4 |
| Q9NZ08 | Endoplasmic reticulum aminopeptidase 1 | ERAP1 | ERAP1 | 327,6 | 1447,4 | 2763,4 | 4867,5 | 1363,8 | 1482,6 | 3392,7 | 2483,4 | 1827,2 | 599,6 | 1277,9 | 7840,3 | 226,8 | 1717,4 | 122,6 | 2449,7 | 981,0 | 5504,7 |
| P01040 | Cystatin-A | CYTA | CSTA | 6193,6 | 6213,5 | 9146,0 | 8997,0 | 8487,9 | 2741,7 | 10405,1 | 13689,4 | 4960,2 | 9120,1 | 8511,5 | 19639,0 | 18445,1 | 1828,5 | 4038,3 | 3991,7 | 10371,1 | 13874,7 |
| P40189 | Interleukin-6 receptor subunit beta | IL6RB | IL6ST | 10884,5 | 7446,2 | 17350,9 | 26389,5 | 9430,9 | 10006,9 | 14082,0 | 13358,7 | 7751,6 | 26891,1 | 15153,7 | 16235,8 | 5044,8 | 11868,6 | 10381,5 | 8559,2 | 22901,9 | 25606,0 |
| Q00796 | Sorbitol dehydrogenase | DHSO | SORD | 10473,6 | 4692,3 | 1437,9 | 3725,5 | 1422,8 | 1542,0 | 2382,1 | 2357,2 | 1733,7 | 816,1 | 457,8 | 2163,9 | 1136,1 | 2065,3 | 848,7 | 769,5 | 1226,7 | 1832,9 |
| P01611 | Immunoglobulin kappa variable 1D-12 | KVD12 | IGKV1D-12 | 1161,1 | 813,7 | 1070,7 | 1054,6 | 1252,3 | 611,8 | 913,7 | 2348,0 | 595,4 | 2681,7 | 603,5 | 1176,6 | 400,9 | 546,4 | 1243,8 | 448,7 | 979,9 | 523,5 |
| P20930 | Filaggrin | FILA | FLG | 9542,2 | 8153,5 | 16424,6 | 470,1 | 2215,7 | 1561,0 | 1782,1 | 10086,0 | 3965,1 | 8147,7 | 980,3 | 12723,2 | 10418,3 | 5694,3 | 15990,0 | 3662,2 | 2898,6 | 7264,7 |
| P07911 | Uromodulin | UROM | UMOD | 2057,5 | 6866,4 | 15309,7 | 7260,5 | 24377,4 | 4825,0 | 14341,5 | 34067,3 | 3743,8 | 2191,8 | 2955,3 | 4156,8 | 4624,7 | 7490,5 | 2716,8 | 28629,2 | 2684,4 | 4221,5 |
| A0A0G2JS06 | Immunoglobulin lambda variable 5-39 | LV539 | IGLV5-39 | 3290,2 | 4252,0 | 494,0 | 4867,0 | 4196,3 | 14124,4 | 1899,0 | 3133,5 | 15241,8 | 3959,1 | 696,1 | 1252,2 | 1623,5 | 943,9 | 4252,4 | 3666,5 | 5446,0 | 3473,7 |
| P37837 | Transaldolase | TALDO | TALDO1 | 3169,0 | 4206,9 | 6089,7 | 9904,9 | 3235,1 | 6688,7 | 6220,7 | 3021,9 | 2690,7 | 2210,6 | 6383,6 | 8456,4 | 1757,2 | 3156,5 | 2385,3 | 2597,5 | 6530,8 | 4385,9 |
| P01344 | Insulin-like growth factor II | IGF2 | IGF2 | 27860,3 | 28566,9 | 39021,5 | 28637,6 | 27508,2 | 39003,9 | 47843,7 | 54985,9 | 47156,2 | 47178,6 | 49071,4 | 49984,3 | 26017,6 | 52959,5 | 34235,7 | 54588,0 | 65976,2 | 70298,7 |
| P28066 | Proteasome subunit alpha type-5 | PSA5 | PSMA5 | 1934,6 | 9377,0 | 7799,1 | 3373,1 | 4209,3 | 4103,3 | 4133,7 | 6679,5 | 4466,9 | 2946,3 | 10306,1 | 27419,2 | 2397,4 | 5887,4 | 3013,1 | 4083,2 | 8803,2 | 4516,3 |
| P55290 | Cadherin-13 | CAD13 | CDH13 | 3990,3 | 12886,4 | 3875,2 | 1458,6 | 6989,3 | 10280,3 | 9766,8 | 7528,3 | 8912,1 | 8134,2 | 6611,0 | 20650,2 | 23888,1 | 5447,7 | 9751,3 | 5191,8 | 4291,5 | 6585,9 |
| Q06278 | Aldehyde oxidase | AOXA | AOX1 | 9082,7 | 8107,7 | 11679,9 | 7038,4 | 4292,6 | 786,9 | 1592,7 | 1686,3 | 5391,6 | 2190,1 | 885,5 | 884,3 | 6620,1 | 1001,6 | 3846,1 | 267,8 | 4053,1 | 8032,4 |
| P20023 | Complement receptor type 2 | CR2 | CR2 | 3941,0 | 13432,1 | 3061,3 | 2088,1 | 2407,1 | 3005,1 | 4264,7 | 10992,5 | 3137,8 | 3638,7 | 2305,7 | 2815,8 | 708,9 | 3376,4 | 1135,4 | 5024,0 | 5542,7 | 2347,4 |
| P49720 | Proteasome subunit beta type-3 | PSB3 | PSMB3 | 670,1 | 1358,8 | 1651,7 | 1514,9 | 954,9 | 2486,2 | 1873,8 | 2394,8 | 274,1 | 1446,8 | 1306,4 | 1276,8 | 6641,5 | 2049,6 | 890,3 | 814,4 | 1432,3 | 767,5 |
| P01834 | Immunoglobulin kappa constant | IGKC | IGKC | 175,4 | 241,7 | 399,1 | 2283,9 | 1271,1 | 69,1 | 1809,3 | 506,6 | 1102,4 | 1563,0 | 584,8 | 533,8 | 874,6 | 1757,4 | 606,2 | 546,4 | 636,1 | 686,2 |
| A0M8Q6 | Immunoglobulin lambda constant 7 | IGLC7 | IGLC7 | 162675,5 | 446683,8 | 101075,1 | 160072,0 | 16684,3 | 40837,0 | 88793,6 | 212870,2 | 42280,5 | 126636,0 | 2417,8 | 54260,8 | 103445,0 | 14699,0 | 16850,7 | 207398,8 | 118757,2 | 126907,1 |
| P04430 | Immunoglobulin kappa variable 1-16 | KV116 | IGKV1-16 | 713,9 | 969,0 | 494,1 | 2217,1 | 3268,9 | 509,3 | 365,2 | 2703,5 | 168,7 | 309,6 | 77,4 | 755,8 | 723,8 | 792,9 | 1221,2 | 1257,4 | 597,9 | 1882,8 |
| P01743 | Immunoglobulin heavy variable 1-46 | HV146 | IGHV1-46 | 640,6 | 329,4 | 4170,4 | 777,2 | 6202,5 | 638,7 | 1550,4 | 1011,8 | 685,2 | 1063,6 | 215,0 | 319,1 | 385,8 | 209,3 | 685,5 | 750,9 | 1596,8 | 326,9 |
| P26022 | Pentraxin-related protein PTX3 | PTX3 | PTX3 | 4685,8 | 13025,4 | 1396,3 | 19200,1 | 2847,2 | 8216,7 | 2880,2 | 2785,1 | 3440,0 | 3590,9 | 3609,8 | 6868,8 | 1160,2 | 2913,2 | 10592,1 | 11553,3 | 8403,5 | 1516,2 |
| P29401 | Transketolase | TKT | TKT | 5314,4 | 13608,9 | 18158,4 | 17881,7 | 14494,9 | 6850,8 | 8955,9 | 5444,8 | 17341,4 | 11187,8 | 3758,1 | 4759,8 | 4935,9 | 8586,2 | 9777,8 | 2439,5 | 11135,0 | 8334,9 |
| P07954 | Fumarate hydratase, mitochondrial | FUMH | FH | 529,6 | 3610,6 | 1820,0 | 370,8 | 1332,2 | 1074,2 | 2291,6 | 2068,6 | 1458,8 | 156,7 | 887,9 | 744,8 | 1955,9 | 1682,4 | 2279,3 | 1730,3 | 1082,6 | 1138,7 |
| P12109 | Collagen alpha-1(VI) chain | CO6A1 | COL6A1 | 3201,5 | 3952,3 | 1278,4 | 1502,7 | 1010,5 | 1802,2 | 2244,0 | 5816,5 | 3974,4 | 4549,4 | 4439,0 | 4751,2 | 5595,8 | 4423,1 | 4443,4 | 4444,0 | 7692,0 | 563,8 |
| P10599 | Thioredoxin | THIO | TXN | 832,3 | 11875,2 | 6375,9 | 25384,2 | 9305,0 | 5247,6 | 26846,9 | 1289,9 | 3576,2 | 16603,8 | 157,3 | 6523,5 | 16323,9 | 7302,0 | 3991,8 | 3139,7 | 2533,8 | 8727,4 |
| Q9BTM1 | Histone H2A.J | H2AJ | H2AFJ | 12442,4 | 27892,5 | 7731,5 | 3538,8 | 9206,6 | 14596,5 | 9983,2 | 6518,7 | 15253,9 | 4039,9 | 7254,7 | 18103,0 | 17725,3 | 18230,8 | 17012,2 | 5491,0 | 19531,6 | 4219,1 |
| P31151 | Protein S100-A7 | S10A7 | S100A7 | 28906,7 | 13018,4 | 20731,9 | 44362,4 | 21655,7 | 25064,1 | 10929,2 | 20518,9 | 8461,2 | 10734,2 | 14790,5 | 159084,4 | 29988,5 | 12462,1 | 10459,6 | 32822,8 | 10417,6 | 11148,9 |
| P28072 | Proteasome subunit beta type-6 | PSB6 | PSMB6 | 109452,5 | 6906,8 | 70289,3 | 79254,3 | 9372,7 | 4899,2 | 5637,5 | 139584,6 | 7141,8 | 12020,1 | 13226,3 | 94010,4 | 14550,0 | 208218,9 | 7892,8 | 284426,7 | 109946,0 | 148953,8 |
| Q16270 | Insulin-like growth factor-binding protein 7 | IBP7 | IGFBP7 | 316,1 | 3628,7 | 1468,9 | 1711,8 | 1236,0 | 1014,5 | 1283,2 | 991,2 | 994,0 | 1844,6 | 476,0 | 3637,8 | 1508,2 | 217,7 | 5667,7 | 1361,6 | 616,2 | 4341,4 |
| Q13867 | Bleomycin hydrolase | BLMH | BLMH | 16767,4 | 10585,1 | 4728,7 | 7482,9 | 14745,0 | 9722,4 | 5683,4 | 11965,2 | 2330,2 | 8290,3 | 7272,9 | 1232,9 | 10674,1 | 5317,5 | 5135,1 | 2448,0 | 14151,1 | 11252,3 |
| Q8TER0 | Sushi, nidogen and EGF-like domain-containing protein 1 | SNED1 | SNED1 | 3441,2 | 17773,7 | 6896,0 | 13958,1 | 4017,5 | 9569,9 | 1829,3 | 4310,0 | 4515,6 | 4337,8 | 6046,9 | 20674,5 | 2162,3 | 7305,7 | 4247,5 | 12844,8 | 6155,2 | 9540,5 |
| P24752 | Acetyl-CoA acetyltransferase, mitochondrial | THIL | ACAT1 | 855,7 | 128,1 | 3384,8 | 1504,5 | 4612,7 | 1476,0 | 2189,0 | 1677,1 | 1203,9 | 2232,0 | 589,4 | 1283,7 | 2250,5 | 2329,6 | 333,2 | 2877,5 | 2160,3 | 1064,5 |
| A1L4H1 | Soluble scavenger receptor cysteine-rich domain-containing protein SSC5D | SRCRL | SSC5D | 4952,4 | 3338,7 | 2184,5 | 911,2 | 3529,0 | 2625,9 | 5756,0 | 3652,8 | 5174,2 | 2436,0 | 922,4 | 16004,0 | 2252,2 | 2427,4 | 8769,7 | 2999,0 | 2130,9 | 1028,8 |
| Q6UWP8 | Suprabasin | SBSN | SBSN | 4734,3 | 4508,5 | 12601,0 | 7317,5 | 4193,6 | 4483,9 | 9470,9 | 5207,8 | 3871,3 | 5591,9 | 2292,1 | 3031,2 | 3216,0 | 2176,7 | 6026,7 | 3072,9 | 704,1 | 2282,1 |
| Q6ZVX7 | F-box only protein 50 | FBX50 | NCCRP1 | 16890,2 | 8035,4 | 24030,0 | 22243,4 | 7720,8 | 24094,0 | 16544,7 | 4564,6 | 4517,8 | 12872,1 | 6227,0 | 3464,2 | 15402,9 | 13355,1 | 12450,8 | 2351,5 | 10339,6 | 14499,2 |
| Q9BYE9 | Cadherin-related family member 2 | CDHR2 | CDHR2 | 426537,4 | 563228,2 | 29571,5 | 56061,1 | 633127,3 | 306637,8 | 16982,6 | 509358,5 | 404530,0 | 346134,5 | 234274,1 | 506729,9 | 393130,6 | 378404,5 | 295601,9 | 470399,9 | 183239,8 | 255909,8 |
| Q9HDC9 | Adipocyte plasma membrane-associated protein | APMAP | APMAP | 18230,5 | 30319,4 | 8398,0 | 10918,1 | 39702,2 | 15482,2 | 29209,8 | 24934,4 | 13017,6 | 48800,5 | 16638,1 | 7326,6 | 15483,4 | 8733,4 | 7674,0 | 13127,5 | 29568,0 | 9763,1 |
| O43184 | Disintegrin and metalloproteinase domain-containing protein 12 | ADA12 | ADAM12 | 4664,8 | 1652,9 | 1668,5 | 38131,9 | 12862,8 | 13678,5 | 19893,2 | 4472,8 | 6642,3 | 5909,4 | 10817,7 | 2655,6 | 11357,7 | 5547,6 | 4546,7 | 19451,0 | 9378,9 | 2851,0 |
| P22528 | Cornifin-B | SPR1B | SPRR1B | 2529,7 | 2569,4 | 866,5 | 1600,3 | 5774,4 | 4164,4 | 279,2 | 1486,7 | 5029,2 | 6316,5 | 467,8 | 1719,7 | 1140,3 | 308,6 | 2110,2 | 1238,3 | 10095,6 | 1572,7 |
| P40925 | Malate dehydrogenase, cytoplasmic | MDHC | MDH1 | 2721,6 | 2896,4 | 3204,1 | 3259,7 | 1141,7 | 6848,7 | 3864,8 | 9023,7 | 1764,7 | 1626,6 | 2263,5 | 3564,5 | 6495,3 | 6253,9 | 912,5 | 1231,9 | 3194,8 | 1309,7 |
| Q96QA5 | Gasdermin-A | GSDMA | GSDMA | 8939,4 | 18492,6 | 10886,3 | 49683,4 | 13054,0 | 8574,1 | 29634,0 | 12904,1 | 37913,6 | 41968,1 | 5893,7 | 11357,5 | 10567,8 | 44750,3 | 2898,8 | 23460,1 | 23489,4 | 9911,2 |
| Q13046 | Putative pregnancy-specific beta-1-glycoprotein 7 | PSG7 | PSG7 | 4436,2 | 5569,5 | 12313,2 | 52232,1 | 9793,7 | 6988,2 | 6087,9 | 9266,2 | 2889,8 | 4272,4 | 9025,4 | 34342,5 | 4865,6 | 12404,1 | 7240,1 | 4319,5 | 15585,3 | 7891,3 |
| Q15517 | Corneodesmosin | CDSN | CDSN | 1632,4 | 145,8 | 618,0 | 738,2 | 2833,0 | 479,0 | 167,5 | 178,1 | 1046,6 | 280,3 | 463,7 | 760,6 | 1641,0 | 96,1 | 1038,5 | 131,0 | 235,3 | 1340,3 |
| A0A0B4J1X5 | Immunoglobulin heavy variable 3-74 | HV374 | IGHV3-74 | 151,8 | 274,5 | 370,5 | 1514,1 | 1687,2 | 1203,0 | 579,3 | 1002,3 | 3126,3 | 1119,9 | 68,2 | 624,9 | 347,3 | 1159,4 | 535,5 | 1130,4 | 3800,3 | 238,7 |
| A0A0C4DH31 | Immunoglobulin heavy variable 1-18 | HV118 | IGHV1-18 | 1449,2 | 4311,8 | 2542,5 | 3022,0 | 5386,8 | 2423,2 | 282,3 | 9544,2 | 5850,7 | 1932,3 | 7648,2 | 8603,5 | 182,3 | 16051,5 | 9796,9 | 3074,2 | 6169,1 | 678,0 |
| P24593 | Insulin-like growth factor-binding protein 5 | IBP5 | IGFBP5 | 10191,1 | 7480,5 | 3840,0 | 16216,7 | 7773,7 | 10224,4 | 12833,2 | 17213,6 | 5232,5 | 10046,3 | 4003,3 | 6614,1 | 3311,0 | 4248,8 | 8970,5 | 3287,9 | 8031,9 | 9291,9 |
| P09619 | Platelet-derived growth factor receptor beta | PGFRB | PDGFRB | 6126,0 | 2054,3 | 3266,3 | 24363,1 | 5257,4 | 6325,1 | 8601,5 | 7613,8 | 9143,4 | 9913,2 | 5491,4 | 2629,8 | 6170,9 | 13257,6 | 1522,8 | 7210,4 | 1575,3 | 2411,8 |
| O15335 | Chondroadherin | CHAD | CHAD | 12334,8 | 6908,9 | 32020,9 | 6635,2 | 105170,3 | 63484,7 | 49441,6 | 5158,5 | 60556,6 | 104493,8 | 5494,6 | 85715,2 | 127761,0 | 16885,1 | 96152,6 | 99762,4 | 2477,6 | 7861,0 |
| Q8WVV4 | Protein POF1B | POF1B | POF1B | 18318,1 | 5265,9 | 3072,9 | 26235,5 | 2064,0 | 14219,4 | 2246,6 | 2948,4 | 2730,0 | 2426,7 | 8586,2 | 13433,8 | 17576,9 | 18423,8 | 10519,6 | 17340,9 | 6560,0 | 2644,5 |
| P30086 | Phosphatidylethanolamine-binding protein 1 | PEBP1 | PEBP1 | 622,9 | 959,0 | 397,6 | 2499,6 | 3127,2 | 1876,8 | 1124,5 | 4612,3 | 784,1 | 1483,1 | 542,1 | 444,5 | 507,0 | 720,4 | 1190,4 | 513,6 | 1198,0 | 1223,2 |
| Q9Y6Z7 | Collectin-10 | COL10 | COLEC10 | 7781,7 | 17511,2 | 48141,2 | 6791,4 | 29498,4 | 8687,1 | 28846,1 | 33352,9 | 18761,7 | 21460,4 | 5477,9 | 61351,8 | 26864,4 | 18109,5 | 20138,2 | 17949,6 | 15073,8 | 12824,8 |
| A0A075B6J9 | Immunoglobulin lambda variable 2-18 | LV218 | IGLV2-18 | 427,7 | 737,0 | 431,3 | 1510,5 | 1147,9 | 966,0 | 317,0 | 481,1 | 141,8 | 391,0 | 1275,3 | 1185,8 | 135,6 | 77,0 | 549,3 | 707,1 | 441,5 | 630,7 |
| Q8IX21 | SMC5-SMC6 complex localization factor protein 2 | SLF2 | SLF2 | 83401,2 | 100983,4 | 86666,1 | 99061,4 | 166322,1 | 196805,6 | 71010,6 | 67287,1 | 12525,1 | 16533,3 | 16748,8 | 8568,9 | 90827,6 | 154033,0 | 94531,6 | 94441,4 | 53365,2 | 61907,8 |
| P55072 | Transitional endoplasmic reticulum ATPase | TERA | VCP | 854,1 | 2244,7 | 4652,3 | 2731,4 | 1428,5 | 6566,2 | 2959,0 | 2479,7 | 3229,3 | 3025,4 | 1694,0 | 6659,8 | 2522,6 | 5027,5 | 2546,2 | 3367,6 | 7825,1 | 1617,2 |
| P0DP09 | Immunoglobulin kappa variable 1-13 | KV113 | IGKV1-13 | 198,5 | 656,8 | 502,5 | 393,6 | 2129,2 | 1293,3 | 625,3 | 205,4 | 307,7 | 532,2 | 2863,7 | 592,7 | 243,3 | 469,7 | 122,6 | 239,2 | 3271,8 | 455,2 |
| P01594 | Immunoglobulin kappa variable 1-33 | KV133 | IGKV1-33 | 560,2 | 537,9 | 1109,1 | 519,5 | 458,2 | 592,3 | 878,0 | 1846,6 | 1089,8 | 271,1 | 854,9 | 1047,0 | 776,4 | 1579,0 | 1725,9 | 1362,6 | 1204,4 | 502,4 |
| Q8NE71 | ATP-binding cassette sub-family F member 1 | ABCF1 | ABCF1 | 112854,7 | 149599,7 | 83857,3 | 68968,6 | 84276,9 | 26062,7 | 42888,3 | 41231,8 | 42411,1 | 133635,2 | 22421,2 | 112610,2 | 38180,7 | 108140,5 | 119693,1 | 171237,5 | 158897,9 | 129636,0 |
| Q96PC5 | Melanoma inhibitory activity protein 2 | MIA2 | MIA2 | 409,7 | 494,3 | 0,0 | 429,1 | 622,5 | 633,4 | 571,1 | 121,5 | 1446,5 | 136,7 | 2713,2 | 409,9 | 461,1 | 712,2 | 377,2 | 709,2 | 838,5 | 0,0 |
| Q9P225 | Dynein heavy chain 2, axonemal | DYH2 | DNAH2 | 9217,4 | 1047,2 | 1716,0 | 561,0 | 674,0 | 1162,6 | 1516,6 | 2328,4 | 647,5 | 1883,8 | 906,7 | 757,7 | 1142,5 | 568,5 | 6158,6 | 2916,7 | 939,9 | 828,4 |
| Q8NFC6 | Biorientation of chromosomes in cell division protein 1-like 1 | BD1L1 | BOD1L1 | 592130,6 | 387500,5 | 593336,1 | 12633,4 | 502793,1 | 638797,8 | 703239,6 | 7402,1 | 549953,9 | 558924,1 | 633767,3 | 450417,9 | 548802,7 | 643241,6 | 9589,4 | 8732,0 | 666425,8 | 523018,7 |
| Q99784 | Noelin | NOE1 | OLFM1 | 26244,1 | 14235,9 | 3241,0 | 22459,5 | 13896,3 | 5337,3 | 38540,5 | 35748,0 | 10853,2 | 12751,9 | 15993,0 | 18761,1 | 4715,1 | 2013,9 | 21148,2 | 6867,1 | 52406,9 | 39848,7 |
| Q15858 | Sodium channel protein type 9 subunit alpha | SCN9A | SCN9A | 4544,2 | 19489,6 | 1448,6 | 4508,4 | 20372,1 | 27363,0 | 27107,8 | 2735,2 | 18099,4 | 6533,2 | 3592,6 | 4112,2 | 4102,6 | 10765,3 | 19569,0 | 4009,1 | 4836,0 | 3355,3 |
| P11362 | Fibroblast growth factor receptor 1 | FGFR1 | FGFR1 | 11022,1 | 37471,8 | 10268,4 | 1502,1 | 2877,0 | 1194,1 | 4857,6 | 2284,7 | 9456,1 | 6511,4 | 649,8 | 24392,5 | 407,7 | 1527,3 | 654,3 | 1787,3 | 3261,5 | 2068,9 |
| P00966 | Argininosuccinate synthase | ASSY | ASS1 | 1610,4 | 897,2 | 3036,2 | 3657,0 | 3814,9 | 6158,9 | 7206,6 | 3178,4 | 6386,3 | 1818,5 | 454,1 | 930,7 | 879,4 | 2765,8 | 2129,5 | 3287,3 | 5952,7 | 2221,0 |
| Q8WWP7 | GTPase IMAP family member 1 | GIMA1 | GIMAP1 | 1749,8 | 319,8 | 1426,4 | 4011,8 | 1081,2 | 1602,0 | 2302,4 | 2406,7 | 2411,3 | 2609,0 | 1413,4 | 2603,0 | 1042,1 | 3036,0 | 1178,9 | 3751,5 | 595,3 | 1418,9 |
| P01706 | Immunoglobulin lambda variable 2-11 | LV211 | IGLV2-11 | 796,5 | 700,3 | 2360,0 | 3290,4 | 3001,6 | 4149,2 | 1665,4 | 3413,1 | 2154,5 | 1344,8 | 417,5 | 7596,2 | 1668,3 | 3129,0 | 4598,9 | 2050,7 | 1164,6 | 4170,6 |
| A0A0C4DH24 | Immunoglobulin kappa variable 6-21 | KV621 | IGKV6-21 | 106,7 | 1159,9 | 741,4 | 3118,9 | 711,0 | 2251,4 | 583,1 | 183,5 | 1012,4 | 547,1 | 69,5 | 968,8 | 112,3 | 294,2 | 470,1 | 881,9 | 920,5 | 371,3 |
