# Supplementary material for: Systemic changes induced by autologous stem cell ovarian transplant in plasma proteome of women with impaired ovarian reserves
Source: Aging (Albany NY). 2023 Dec 26;15(24):14553–73. doi: 10.18632/aging.205400 (PMC10781467; doi:10.18632/aging.205400)
Supplement: Supplementary Tables 2-5 and 7-10 [file aging-15-205400-s004.pdf]

SUPPLEMENTARY TABLES

Supplementary Table 2. Differentially expressed proteins between PRE and APHERESIS samples in women with poor ovarian response (POR).

Protein ID	Protein name	Protein abbreviation name	Gene name	Estimate
**P02750**	Leucine-rich alpha-2-glycoprotein	A2GL	LRG1	1,771
**P01042**	Kininogen-1	KNG1	KNG1	1,638
**P02746**	Complement C1q subcomponent subunit B	C1QB	C1QB	1,562
**P10909**	Clusterin	CLUS	CLU	1,299
**P02748**	Complement component C9	CO9	C9	1,053
**P08185**	Corticosteroid-binding globulin	CBG	SERPINA6	0,983
**P06727**	Apolipoprotein A-IV	APOA4	APOA4	0,953
**P0C0L4**	Complement C4-A	CO4A	C4A	0,727
**P00748**	Coagulation factor XII	FA12	F12	0,664
**P02768**	Serum albumin	ALBU	ALB	–0,414
**P13473**	Lysosome-associated membrane glycoprotein 2	LAMP2	LAMP2	–1,362

Supplementary Table 3. Differentially expressed proteins between POST and APHERESIS samples in women with poor ovarian response (POR).

Protein ID	Protein name	Protein abbreviation name	Gene name	Estimate
**Q8TER0**	Sushi, nidogen and EGF-like domain-containing protein 1	SNED1	SNED1	2,335
**P13796**	Plastin-2	PLSL	LCP1	2,164
**P19320**	Vascular cell adhesion protein 1	VCAM1	VCAM1	2,133
**P11597**	Cholesteryl ester transfer protein	CETP	CETP	1,863
**P18428**	Lipopolysaccharide-binding protein	LBP	LBP	1,788
**P14543**	Nidogen-1	NID1	NID1	1,709
**P08571**	Monocyte differentiation antigen CD14	CD14	CD14	1,647
**P02679**	Fibrinogen gamma chain	FIBG	FGG	1,624
**P0DOX8**	Immunoglobulin lambda-1 light chain	IGL1	IGL1	1,624
**O00533**	Neural cell adhesion molecule L1-like protein	NCHL1	CHL1	1,623
**P00751**	Complement factor B	CFAB	CFB	1,615
**P19827**	Inter-alpha-trypsin inhibitor heavy chain H1	ITIH1	ITIH1	1,551
**P04275**	von Willebrand factor	VWF	VWF	1,505
**P07360**	Complement component C8 gamma chain	CO8G	C8G	1,455
**P10643**	Complement component C7	CO7	C7	1,439
**P00488**	Coagulation factor XIII A chain	F13A	F13A1	1,437
**P02746**	Complement C1q subcomponent subunit B	C1QB	C1QB	1,404
**P12259**	Coagulation factor V	FA5	F5	1,390
**P19823**	Inter-alpha-trypsin inhibitor heavy chain H2	ITIH2	ITIH2	1,379
**P05546**	Heparin cofactor 2	HEP2	SERPIND1	1,376
**P01011**	Alpha-1-antichymotrypsin	AACT	SERPINA3	1,293
**P00748**	Coagulation factor XII	FA12	F12	1,243
**P29622**	Kallistatin	KAIN	SERPINA4	1,234
**P06396**	Gelsolin	GELS	GSN	1,231

**P09871**	Complement C1s subcomponent	C1S	C1S	1,217
**Q12805**	EGF-containing fibulin-like extracellular matrix protein 1	FBLN3	EFEMP1	1,207
**P0C0L4**	Complement C4-A	CO4A	C4A	1,198
**P02748**	Complement component C9	CO9	C9	1,186
**P05156**	Complement factor I	CFAI	CFI	1,145
**P23142**	Fibulin-1	FBLN1	FBLN1	1,114
**P07225**	Vitamin K-dependent protein S	PROS	PROS1	1,106
**P09211**	Glutathione S-transferase P	GSTP1	GSTP1	1,104
**P00740**	Coagulation factor IX	FA9	F9	1,098
**P15169**	Carboxypeptidase N catalytic chain	CBPN	CPN1	1,058
**P14151**	L-selectin	LYAM1	SELL	1,051
**P80748**	Immunoglobulin lambda variable 3-21	LV321	IGLV3-21	1,015
**P02675**	Fibrinogen beta chain	FIBB	FGB	1,002
**P05543**	Thyroxine-binding globulin	THBG	SERPINA7	0,990
**Q06033**	Inter-alpha-trypsin inhibitor heavy chain H3	ITIH3	ITIH3	0,971
**P36955**	Pigment epithelium-derived factor	PEDF	SERPINF1	0,944
**Q9BXR6**	Complement factor H-related protein 5	FHR5	CFHR5	0,934
**P01042**	Kininogen-1	KNG1	KNG1	0,930
**P43251**	Biotinidase	BTD	BTD	0,903
**P55058**	Phospholipid transfer protein	PLTP	PLTP	0,873
**P08185**	Corticosteroid-binding globulin	CBG	SERPINA6	0,835
**Q9UK55**	Protein Z-dependent protease inhibitor	ZPI	SERPINA10	0,815
**P22792**	Carboxypeptidase N subunit 2	CPN2	CPN2	0,794
**Q96KN2**	Beta-Ala-His dipeptidase	CNDP1	CNDP1	0,785
**Q14624**	Inter-alpha-trypsin inhibitor heavy chain H4	ITIH4	ITIH4	0,784
**Q9P225**	Dynein heavy chain 2, axonemal	DYH2	DNAH2	0,755
**P05155**	Plasma protease C1 inhibitor	IC1	SERPING1	0,751
**P01703**	Immunoglobulin lambda variable 1-40	LV140	IGLV1-40	0,720
**A0A0C4DH38**	Immunoglobulin heavy variable 5-51	HV551	IGHV5-51	0,719
**P01009**	Alpha-1-antitrypsin	A1AT	SERPINA1	0,716
**P06276**	Cholinesterase	CHLE	BCHE	0,712
**P01008**	Antithrombin-III	ANT3	SERPINC1	0,623
**O14791**	Apolipoprotein L1	APOL1	APOL1	0,616
**P08697**	Alpha-2-antiplasmin	A2AP	SERPINF2	0,602
**Q92820**	Gamma-glutamyl hydrolase	GGH	GGH	0,584
**Q9Y6R7**	IgGFc-binding protein	FCGBP	FCGBP	0,534
**P01624**	Immunoglobulin kappa variable 3-15	KV315	IGKV3-15	0,415
**P12035**	Keratin, type II cytoskeletal 3	K2C3	KRT3	–0,383
**P02768**	Serum albumin	ALBU	ALB	–0,543
**P43652**	Afamin	AFAM	AFM	–0,613
**P02656**	Apolipoprotein C-III	APOC3	APOC3	–0,884
**P02774**	Vitamin D-binding protein	VTDB	GC	–0,932
**Q13103**	Secreted phosphoprotein 24	SPP24	SPP2	–0,948
**P13473**	Lysosome-associated membrane glycoprotein 2	LAMP2	LAMP2	–1,163
**P32119**	Peroxiredoxin-2	PRDX2	PRDX2	–2,298
**P69905**	Hemoglobin subunit alpha	HBA	HBA1	–3,616

The most prominent changes after ASCOT, considering the estimated fold change, are highlighted in yellow (upregulations) or blue (downregulations).

Supplementary Table 4. Ovarian reserve biomarkers of women with premature ovarian insufficiency (POI) before autologous stem cell ovarian transplantation (ASCOT) and three months after this reactivation technique.

Parameter	All (*n* = 6)		ASCOT arm (*n* = 3)		Mobilization arm (*n* = 3)	
	PRE	POST	PRE	POST	PRE	POST
**AMH (pmol/L)**	0.1 ± 0.1	0.3 ± 0.3*	0.1 ± 0.0	0.4 ± 0.3	0.1 ± 0.1	0.2 ± 0.1
**AFC (n)**	1.5 ± 1.2	2.5 ± 2.6	1.3 ± 0.9	2.0 ± 2.0	1.7 ± 1.5	3.0 ± 2.8
**FSH (IU/mL)**	99.9 ± 23.9	54.9 ± 35.9*	100.3 ± 17.3	60.2 ± 38.2*	99.5 ± 31.2	49.6 ± 33.6*

Wilcoxon nonparametric paired test was applied to compare PRE and POST ovarian reserve biomarkers for each patient, indicating the asterisk a *p*-value < 0.05. Abbreviations: AMH: anti-müllerian hormone; AFC: antral follicular count; FSH: follicle-stimulating hormone.

Supplementary Table 5. Protocols of controlled ovarian stimulation (COS) performed in patients with premature ovarian insufficiency (POI) after autologous stem cell ovarian transplantation (ASCOT) technique.

Parameter	All (*n* = 6)	ASCOT arm (*n* = 3)	Mobilization arm (*n* = 3)
**COS cycles (*n* total)**	11	5	6
**FSH (IU/mL)**	39.7 ± 16.8	38.1 ± 13.4	41.0 ± 20.4
**AMH (pM)**	0.4 ± 0.6	0.7 ± 0.8	0.1 ± 0.1
**Days of stimulation**	8.6 ± 5.8	5.4 ± 5.9	11.3 ± 4.6
**E2 hCG day**	189.2 ± 182.5	276.5 ± 222.2	116.3 ± 114.7
**AFC total**	1.6 ± 0.9	1.8 ± 1.1	1.5 ± 0.8
**Punctured follicles**	0.8 ± 0.4	1.0 ± 0.0	0.7 ± 0.5
**MII oocytes**	0.3 ± 0.5	0.4 ± 0.6	0.2 ± 0.4
**Embryos**	0.3 ± 0.5	0.3 ± 0.5	0.3 ± 0.5
**Cancellation**	2/11 (18.2%)	0/5 (0%)	2/6 (33.3%)

Supplementary Table 7. Differentially expressed proteins between all PRE and APHERESIS samples in women with premature ovarian insufficiency (POI).

Protein ID	Protein name	Protein abbreviation name	Gene name	Estimate
**P02745**	Complement C1q subcomponent subunit A	C1QA	C1QA	2,75
**P00736**	Complement C1r subcomponent	C1R	C1R	2,70
**P14151**	L-selectin	LYAM1	SELL	1,45
**P59666**	Neutrophil defensin 3	DEF3	DEFA3	0,81
**P00740**	Coagulation factor IX	FA9	F9	0,65
**P08571**	Monocyte differentiation antigen CD14	CD14	CD14	0,51
**P02748**	Complement component C9	CO9	C9	0,51
**P61626**	Lysozyme C	LYSC	LYZ	0,43
**P02747**	Complement C1q subcomponent subunit C	C1QC	C1QC	0,35
**P21333**	Filamin-A	FLNA	FLNA	0,25
**P0DOX5**	Immunoglobulin gamma-1 heavy chain	IGG1	1 SV	0,22
**P02741**	C-reactive protein	CRP	CRP	–0,21
**P32754**	4-hydroxyphenylpyruvate dioxygenase	HPPD	HPD	–0,28
**P11362**	Fibroblast growth factor receptor 1	FGFR1	FGFR1	–0,43

Supplementary Table 8. Differentially expressed proteins between PRE and POST samples in the mobilization arm.

Protein ID	Protein name	Protein abbreviation name	Gene name	Estimate
**P01344**	Insulin-like growth factor II	IGF2	IGF2	0,66
**P05109**	Protein S100-A8	S10A8	S100A8	0,51
**Q15582**	Transforming growth factor-beta-induced protein ig-h3	BGH3	TGFBI	0,45
**P05451**	Lithostathine-1-alpha	REG1A	REG1A	0,30
**Q8IX21**	SMC5-SMC6 complex localization factor protein 2	SLF2	SLF2	0,27
**P02776**	Platelet factor 4	PLF4	PF4	0,25
**P15924**	Desmoplakin	DESP	DSP	0,25
**P01861**	Immunoglobulin heavy constant gamma 4	IGHG4	IGHG4	0,24
**O43866**	CD5 antigen-like	CD5L	CD5L	0,15
**P07996**	Thrombospondin-1	TSP1	THBS1	0,14
**P08253**	72 kDa type IV collagenase	MMP2	MMP2	0,12
**P33908**	Mannosyl-oligosaccharide 1,2-alpha-mannosidase IA	MA1A1	MAN1A1	0,12
**P00966**	Argininosuccinate synthase	ASSY	ASS1	0,11
**P02671**	Fibrinogen alpha chain	FIBA	FGA	0,10
**P08709**	Coagulation factor VII	FA7	F7	0,06
**P0DML3**	Chorionic somatomammotropin hormone 2	CSH2	CSH2	0,05
**P00450**	Ceruloplasmin	CERU	CP	0,04
**P09972**	Fructose-bisphosphate aldolase C	ALDOC	ALDOC	0,02
**P11226**	Mannose-binding protein C	MBL2	MBL2	–0,01
**P11021**	Endoplasmic reticulum chaperone BiP	BIP	HSPA5	–0,01
**P04003**	C4b-binding protein alpha chain	C4BPA	C4BPA	–0,08
**Q12860**	Contactin-1	CNTN1	CNTN1	–0,20
**O75882**	Attractin	ATRN	ATRN	–0,24
**P33151**	Cadherin-5	CADH5	CDH5	–0,54

Supplementary Table 9. Differentially expressed proteins between PRE and POST samples in the ASCOT arm. Table supplied in excel format.

Protein ID	Protein name	Protein abbreviation name	Gene name	Estimate
**P61769**	Beta-2-microglobulin	B2MG	B2M	0,35
**P01344**	Insulin-like growth factor II	IGF2	IGF2	0,23
**Q01459**	Di-N-acetylchitobiase	DIAC	CTBS	–0,02
**P31944**	Caspase-14	CASPE	CASP14	–0,33
**Q7Z7G0**	Target of Nesh-SH3	TARSH	ABI3BP	–0,35
**Q8NE71**	ATP-binding cassette sub-family F member 1	ABCF1	ABCF1	–0,43
**P06396**	Gelsolin	GELS	GSN	–0,49
**P43251**	Biotinidase	BTD	BTD	–0,50
**O00299**	Chloride intracellular channel protein 1	CLIC1	CLIC1	–0,52
**P05160**	Coagulation factor XIII B chain	F13B	F13B	–0,61
**P01031**	Complement C5	CO5	C5	–2,97

Supplementary Table 10. Differentially expressed proteins between POST samples of both arms of the study (mobilization and ASCOT). Table supplied in excel format.

Protein ID	Protein name	Protein abbreviation name	Gene name	Estimate
**P06727**	Apolipoprotein A-IV	APOA4	APOA4	0,64
**P08697**	Alpha-2-antiplasmin	A2AP	SERPINF2	0,56
**Q16706**	Alpha-mannosidase 2	MA2A1	MAN2A1	0,49
**P01861**	Immunoglobulin heavy constant gamma 4	IGHG4	IGHG4	0,22
**P23142**	Fibulin-1	FBLN1	FBLN1	0,00
**Q13093**	Platelet-activating factor acetylhydrolase	PAFA	PLA2G7	–0,39
**Q06033**	Inter-alpha-trypsin inhibitor heavy chain H3	ITIH3	ITIH3	–0,48
**P0DOX5**	Immunoglobulin gamma-1 heavy chain	IGG1	1 SV	–0,65
**P02775**	Platelet basic protein	CXCL7	PPBP	–0,66
**P13646**	Keratin, type I cytoskeletal 13	K1C13	KRT13	–0,88
**P02746**	Complement C1q subcomponent subunit B	C1QB	C1QB	–1,04
**Q13822**	Ectonucleotide pyrophosphatase/phosphodiesterase family member 2	ENPP2	ENPP2	–1,69
**P07225**	Vitamin K-dependent protein S	PROS	PROS1	–1,95
**P01023**	Alpha-2-macroglobulin	A2MG	A2M	–2,11
**P80108**	Phosphatidylinositol-glycan-specific phospholipase D	PHLD	GPLD1	–2,60
**P48740**	Mannan-binding lectin serine protease 1	MASP1	MASP1	–3,84
